# Design, Synthesis, and Antitubercular Activity of Thiolutin–Cycloserine Hybrids: Reducing Cytotoxicity

**DOI:** 10.3390/microorganisms14081670

**Published:** 2026-07-30

**Authors:** Zhibin Sun, Xiaolong Chen, Shanfeng Shi, Yongqi Mu, Chuanxing Wan, Guoguo He

**Affiliations:** Key Laboratory of Conservation and Utilization of Biological Resources in the Tarim Basin, College of Life Science and Technology, Tarim University, Alaer 843300, China

**Keywords:** antitubercular hybrids, thiolutin, cycloserine, selectivity index

## Abstract

The development of dual-acting hybrid antibiotics is a promising strategy to combat the ongoing spread of drug-resistant tuberculosis. Inspired by the structure of the natural dithiolopyrrolone hybrid antibiotic thiomarinol and based on the synergistic effects confirmed by checkerboard assays, we employed a molecular hybridization strategy. The dithiolopyrrolone natural product thiolutin was covalently linked to the pharmacophores of four clinical antitubercular drugs—cycloserine, linezolid, isoniazid, and pyrazinamide—through alkyl linkers of 7–10 carbon atoms via amide condensation, leading to the design and synthesis of 15 novel hybrids. In vitro antitubercular activity evaluation revealed that the cycloserine series exhibited the best activity, with MIC values as low as 1 μg/mL, followed by the isoniazid series. Cytotoxicity assays showed that all cycloserine hybrids had IC_50_ values > 40 μg/mL against RAW 264.7 mouse macrophages, markedly lower than that of thiolutin alone. Among them, T_1_-CS and T_4_-CS displayed the best selectivity indices, achieving an effective reduction in cytotoxicity. This study successfully constructed a class of thiolutin–cycloserine hybrids with low cytotoxicity and high selectivity, providing a valuable molecular template for the discovery of novel antitubercular lead compounds.

## 1. Introduction

Tuberculosis (TB), caused by *Mycobacterium tuberculosis* (MTB), continues to pose a serious global public health challenge [1]. First- and second-line anti-TB drugs are currently the mainstay of clinical treatment. However, their long treatment courses and severe toxicities commonly result in poor patient adherence, which accelerates the emergence of drug-resistant MTB strains [2]. Accordingly, it is urgent to develop new therapeutic strategies against tuberculosis [3].

To overcome the inherent limitations of conventional small-molecule drugs, researchers are currently carrying out active explorations along two major directions. One direction focuses on innovations in drug delivery and physical bactericidal approaches [4], leading to the successive development of novel technologies such as nanomaterial-based therapeutic agents [5,6] and biomimetic nanostructured interfaces [7]. These strategies can both disrupt bacterial cell structures through physical actions and achieve targeted delivery of antimicrobial agents. Leveraging the unique physicochemical properties and mechanical bactericidal mechanisms at the nanoscale, these non-chemical sterilization approaches are reliably effective, can circumvent classical bacterial resistance pathways, and offer sustainable solutions for the subsequent application of antimicrobial drugs.

The other direction focuses on the structural remodeling of drug molecules themselves, among which dual-acting hybrid antibiotics represent a highly promising research avenue for combating drug-resistant tuberculosis and other bacterial infections [8]. Such molecules covalently link two pharmacophores with different mechanisms via a metabolically stable linker, presenting a single entity with fixed pharmacokinetic properties and enabling multi-target synergy that enhances antibacterial activity and reduces resistance development [9,10]. Among various hybrid antibiotics, natural products featuring a dithiolopyrrolone (DTP) core have drawn considerable attention due to their unique two-component covalent linkage, offering valuable inspiration for the design of artificial hybrid molecules [11].

Currently, the class of DTP natural products encompasses around 30 known compounds, with thiolutin, holomycin, and aureothricin being among the most representative [12,13]. These members display broad-spectrum bioactivity, ranging from antifungal and anti-tumor effects to antibacterial activity against Gram-positive and Gram-negative bacteria [13,14]. However, DTPs like thiolutin also display cytotoxicity towards eukaryotic cells, which severely limits their direct druggability. Reducing cytotoxicity while preserving the unique antibacterial mechanism of these natural products remains a key challenge in their development. Notably, nature provides a hybrid antibiotic composed of a DTP unit—thiomarinol—which serves as an important example for overcoming the above difficulty.

**Figure 1:**
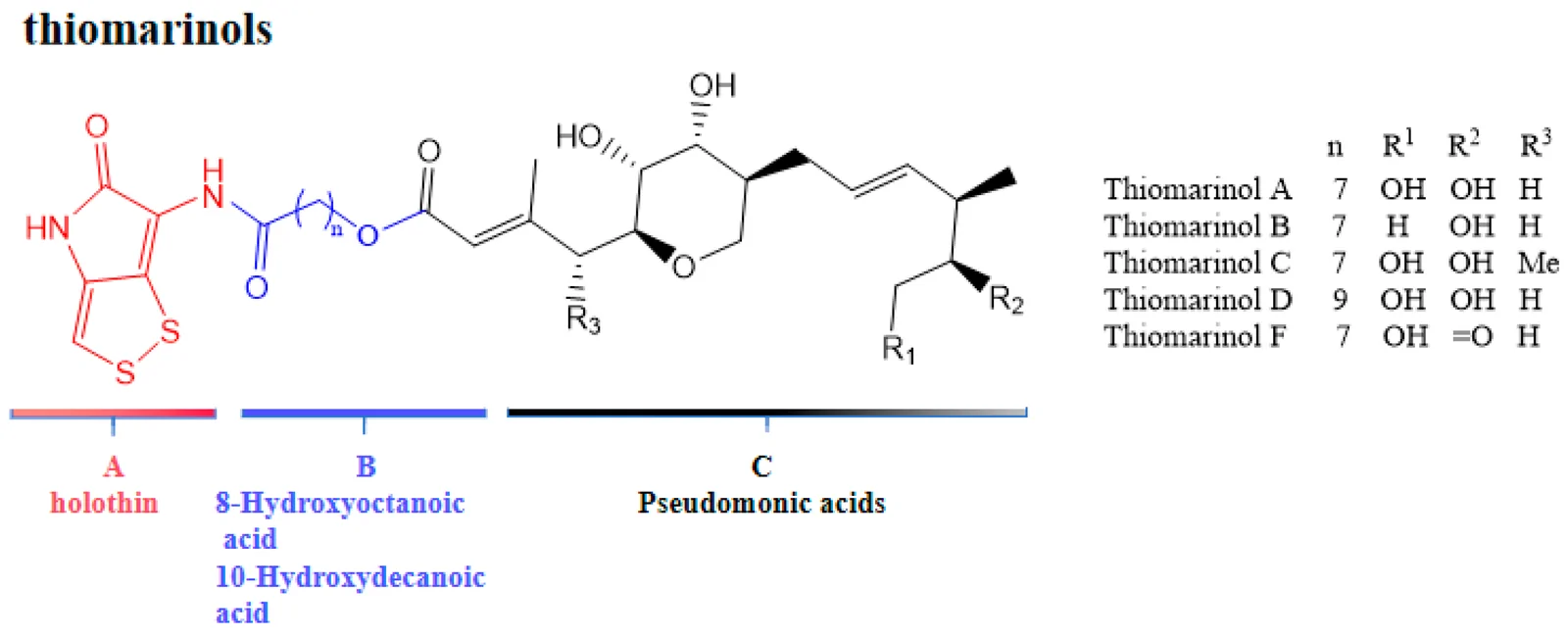
Chemical structure of thiomarinol. (**A**) Holothin, the DTP unit; (**B**) fatty acyl amide bridge of 8 or 10 carbon atoms; (**C**) pseudomonic acid analogue (marinolic acid).

Thiomarinol contains a DTP moiety (holothin, Figure 1A) and a pseudomonic acid analogue (Figure 1C) [15], joined covalently by an 8- or 10-carbon fatty acyl amide bridge (Figure 1B). Although the mechanism of DTP against MTB remains poorly investigated, studies have shown that the DTP unit acts as a “privileged structure”, not only helping the hybrid molecule achieve high intracellular accumulation in bacteria, penetrate the outer membrane of Gram-negative bacteria, and evade efflux pumps, but also extending the antibacterial spectrum of pseudomonic acid from Gram-positive to Gram-negative bacteria [11]. More importantly, thiomarinol retains activity against mupirocin-resistant MRSA, and the rate of resistance development is substantially lower than that for mupirocin. This advantage originates from its dual-action mode: the DTP unit disrupts intracellular metal homeostasis through broad-spectrum chelation of divalent metal ions, while the pseudomonic acid unit targets isoleucyl-tRNA synthetase (IleRS) [16,17,18]. In contrast, single-target antibiotics are prone to resistance via point mutations in the target-encoding gene and often fail to penetrate the complex defense systems of multidrug-resistant bacteria [19].

Inspired by the above, we first confirmed the synergistic antibacterial effect of thiolutin in combination with isoniazid (INH), cycloserine (CS), apyrazinamide (PZA), linezolid (LZD) against *M. tuberculosis* H37Ra using checkerboard assays, providing an experimental basis for hybrid design. Using 7–10 carbon alkyl linkers, we then covalently conjugated the DTP scaffold of thiolutin to the pharmacophores of INH, CS, PZA and LZD via amide bonds, generating 15 novel hybrid molecules. Considering biosafety, the attenuated strain H37Ra was employed as the primary model for profiling antimycobacterial activity and mammalian cytotoxicity, with the aim of identifying promising lead compounds and delineating preliminary structure–activity relationships.

## 2. Materials and Methods

### 2.1. Chemical Synthesis

The carboxylic acid intermediates T_1_–T_4_ (1.0 eq) were dissolved in anhydrous acetonitrile, and DIPEA (3.0 eq) was added as an organic base. The reaction mixture was cooled to 0 °C and stirred for 5 min, then HATU (1.2 eq) was added as a coupling reagent to activate the carboxylic acid group. Stirring was continued at 0 °C for an additional 10 min to ensure complete conversion of the carboxylic acid functionality into the activated ester intermediate.

Subsequently, the corresponding amine coupling partner (cycloserine, linezolid, isoniazid, or 2-aminopyridine, 1.2 eq) was added dropwise to the pre-activated reaction mixture. After the addition was complete, the reaction was allowed to warm to room temperature and stirred continuously for 6–8 h. Upon completion of the reaction, the organic solvent was removed under reduced pressure to afford a crude residue. The crude product was purified by silica gel column chromatography using gradient elution with a dichloromethane–methanol solvent system (100:1 to 5:1), with the specific eluent composition adjusted according to the polarity of each target compound. This general synthetic procedure was applied to all four carboxylic acid intermediates (*n* = 5, 6, 7, 8); each intermediate was coupled with each of the four amine partners (CS, LZD, INH, and 2AP), ultimately affording a library of 15 target hybrid compounds.

#### 2.1.1. Cycloserine (CS) Series

(*R*)-N^1^-(4-methyl-5-oxo-4,5-dihydro-[1,2]dithiolo[4,3-b]pyrrol-6-yl)-N^7^-(3-oxoisoxazolidin-4-yl)heptanediamide (T_1_-CS). Yellow solid (37%); Rf = 0.35 (CHCl_3_/MeOH = 3:1, *v*/*v*). ^1^H NMR (500 MHz, DMSO-*d*6) δ 1.24 (t, *J* = 9.3, 6.1 Hz, 2H), 1.50 (m, 4H), 2.12 (t, *J* = 7.3 Hz, 2H), 2.33 (t, *J* = 7.4 Hz, 2H), 3.25 (s, 3H), 3.88 (dd, *J* = 9.8, 8.4 Hz, 1H), 4.49 (t, *J* = 8.4 Hz, 1H), 4.73 (d, *J* = 8.9 Hz, 1H), 7.33 (s, 1H), 8.38 (d, *J* = 7.8 Hz, 1H), 9.95 (s, 1H), 11.49 (s, 1H). ^13^C NMR (125 MHz, DMSO-*d*_6_) δ 172.57, 171.80, 166.20, 135.98, 132.39, 114.78, 110.90, 72.51, 53.92, 34.88, 34.52, 28.13, 27.54, 24.87, 24.84. The HRMS (ESI) spectrum of C_16_H_21_N_4_O_5_S_2_ exhibited a peak at *m*/*z* 413.0887 for [M + H]^+^ (calcd. 413.0953). Evaluation by HPLC gave a purity of 97.2% and a retention time of 11.113 min, using a mobile phase consisting of 5% CH_3_CN in 95% H_2_O. The NMR and HRMS data for the other CS conjugates T_2_-CS, T_3_-CS, and T_4_-CS were nearly identical to those of T_1_-CS, showing only incremental methylene signals in the aliphatic region.

#### 2.1.2. Linezolid (LZD) Series

2-butyl-N^1^-(((*R*)-3-(3-fluoro-4-morpholinophenyl)-2-oxooxazolidin-5-yl)methyl)-N^3^-(4-methyl-5-oxo-4,5-dihydro-[1,2]dithiolo[4,3-*b*]pyrrol-6-yl)malonamide (T_1_-LZD). Yellow solid (64%); Rf = 0.5 (CHCl_3_/MeOH = 20:1, *v*/*v*). ^1^H NMR (500 MHz, DMSO-*d*6) δ 1.17 (m, 2H), 1.48–1.36 (m, 4H), 2.08 (t, *J* = 8.5 Hz, 2H), 2.27 (t, *J* = 7.4 Hz, 2H), 2.93 (t, *J* = 4.6 Hz, 4H), 3.24 (s, 3H), 3.38 (t, *J* = 5.1 Hz, 1H), 3.45 (dt, *J* = 14.4, 5.8 Hz, 1H), 3.70 (m, 5H), 4.07 (t, *J* = 9.0 Hz, 1H), 4.71 (m, 1H), 7.04 (t, *J* = 9.3 Hz, 1H), 7.15 (dd, *J* = 8.7, 2.6 Hz, 1H), 7.32 (s, 1H), 7.48 (dd, *J* = 15.0, 2.5 Hz, 1H), 8.21 (t, *J* = 6.0 Hz, 1H), 9.92 (s, 1H). ^13^C NMR (125 MHz, DMSO-*d*6) δ 173.08, 171.78, 166.18, 154.06, 153.62, 135.97, 135.44, 133.40, 132.35, 119.26, 114.78, 113.95, 110.88, 106.60, 106.39, 71.57, 66.16, 50.71, 50.69, 47.20, 41.26, 35.15, 34.50, 28.17, 27.52, 25.10, 24.83. The HRMS (ESI) spectrum of C_27_H_33_FN_5_O_6_S_2_ exhibited a peak at *m*/*z* 606.1856 for [M + H]^+^ (calcd. 606.1763). Evaluation by HPLC gave a purity of 98.4% and a retention time of 18.052 min, using a mobile phase consisting of 5% CH_3_CN in 95% H_2_O.The longer-chain LZD analogs T_2_-LZD, T_3_-LZD, and T_4_-LZD gave very similar NMR profiles.

#### 2.1.3. Isoniazid (INH) Series

*N*-(4-Methyl-5-oxo-4,5-dihydro-[1,2]dithiolo[4,3-*b*]pyrrol-6-yl)-2-(2-isonicotinoylhydrazine-1-carbonyl)hexanamide (T_1_-INH). Yellow solid (58%); Rf = 0.48 (CHCl_3_/MeOH = 10:1, *v*/*v*). ^1^H NMR (500 MHz, DMSO-*d*6) δ 1.38–1.27 (m, 2H), 1.60–1.50 (m, 4H), 2.19 (t, *J* = 7.4 Hz, 2H), 2.36 (t, *J* = 7.4 Hz, 2H), 3.25 (s, 3H), 7.34 (s, 1H), 7.83–7.74 (m, 2H), 8.84–8.71 (m, 2H), 9.97 (d, *J* = 2.3 Hz, 2H), 10.62 (s, 1H). ^13^C NMR (125 MHz, DMSO-*d*6) δ 171.80, 171.49, 166.21, 163.93, 150.43, 139.51, 135.99, 132.42, 121.33, 114.80, 110.94, 34.56, 33.16, 28.15, 27.55, 24.84. The HRMS (ESI) spectrum of C_19_H_22_N_5_O_4_S_2_ exhibited a peak at *m*/*z* 448.1113 for [M + H]^+^ (calcd. 448.1058). Evaluation by HPLC gave a purity of 95.8% and a retention time of 12.651 min, using a mobile phase consisting of 5% CH_3_CN in 95% H_2_O. The remaining INH hybrids T_2_-INH, T_3_-INH and T_4_-INH exhibit similar ^1^H and ^13^C NMR spectral features.

#### 2.1.4. 2-Aminopyrazine (2AP) Series

N^1^-(4-Methyl-5-oxo-4,5-dihydro-[1,2]dithiolo[4,3-*b*]pyrrol-6-yl)-2-pentyl-N^3^-(pyrazin-2-ylmethyl)malonamide (T_2_-2AP). Yellow solid (77%); Rf = 0.66 (CHCl_3_/MeOH = 30:1, *v*/*v*). ^1^H NMR (500 MHz, DMSO-*d*_6_) δ 1.25 (m, 4H), 1.51 (m, 4H), 2.15 (t, *J* = 7.3 Hz, 2H), 2.33 (t, *J* = 7.3 Hz, 2H), 3.25 (s, 3H), 4.39 (d, *J* = 5.7 Hz, 2H), 7.33 (s, 1H), 8.48 (t, *J* = 5.9 Hz, 1H), 8.60–8.51 (m, 3H), 9.94 (s, 1H). ^13^C NMR (125 MHz, DMSO-*d*6) δ 172.55, 171.83, 166.21, 154.46, 143.90, 143.25, 143.12, 135.98, 132.42, 114.78, 110.90, 42.23, 35.19, 34.65, 28.41, 28.36, 27.54, 25.11, 25.00. The HRMS (ESI) spectrum of C_19_H_24_N_5_O_3_S_2_ exhibited a peak at *m*/*z* 434.1321 for [M + H]^+^ (calcd. 434.1325). Evaluation by HPLC gave a purity of 97.2% and a retention time of 14.821 min, using a mobile phase consisting of 5% CH_3_CN in 95% H_2_O. The other 2AP hybrids T_3_-2AP and T_4_-2AP showed analogous spectroscopic features, with full data available in the Supporting Information.

Full characterization information, including NMR and HRMS spectra for each target compound, is provided in the Supporting Materials for reference.

### 2.2. Biological Experiments

#### 2.2.1. Culture of *M. tuberculosis* H37Ra and RAW 264.7 Cells

RAW 264.7 cells (Servicebio, Wuhan, China, STCC20020G-1) were maintained in RPMI 1640 medium containing 10% FBS under 37 °C and 5% CO_2_. For the mycobacterial strain, MTB H37Ra was propagated in Middlebrook 7H9 liquid medium (BD Difco, Sparks, MD, USA, #271310) at the same temperature. The growth medium was fortified with 0.5% glycerol, 0.05% Tween-80, and OADC enrichment. To evaluate MIC, a resazurin-based microplate assay was employed. In detail, a suspension of H37Ra cells in the logarithmic growth phase was prepared using Middlebrook 7H9 broth supplemented as described above (BD BBL, Sparks, MD, USA, #212351).

#### 2.2.2. Microbroth Alamar Blue Assay

MTB H37Ra was cultured in Middlebrook 7H9 broth at 37 °C with static incubation until reaching mid-log phase (OD_600_ = 0.5), then diluted 500-fold with fresh 7H9 broth to approximately 2 × 10^5^ CFU/mL. Test compounds were dissolved in DMSO to prepare stock solutions and subsequently serially diluted two-fold to generate working solutions. The assay was performed in sterile 96-well flat-bottom microplates, with each well receiving 100 μL of bacterial suspension and 100 μL of compound working solution (final volume 200 μL; final DMSO concentration ≤ 1% *v*/*v*). Each plate included a growth control and a sterility control. The plates were sealed and incubated statically at 37 °C for 7 days. Following incubation, 10 μL of 1 mg/mL resazurin solution was added to each well, followed by incubation at 37 °C in the dark for 24 h. Metabolically active bacteria reduce blue resazurin to pink resorufin. The MIC was defined as the lowest compound concentration that retained a blue color upon visual inspection. Each compound concentration was tested in triplicate [20].

#### 2.2.3. Checkerboard Assay for Thiolutin in Combination with Antitubercular Drugs

MTB H37Ra was cultured to the logarithmic growth phase and the bacterial suspension was adjusted to 2 × 10^5^ CFU/mL using Middlebrook 7H9 medium. Thiolutin was dissolved in DMSO and then serially diluted with Middlebrook 7H9 medium to a maximum working concentration of 4 μg/mL. CS, INH, LZD, and 2AP were similarly serially diluted with the medium, with maximum working concentrations of 256 μg/mL, 1 μg/mL, 4 μg/mL, and 1024 μg/mL, respectively. In a 96-well plate, 50 μL of serially diluted thiolutin was added vertically down each column, and 50 μL of serially diluted antitubercular drug was added horizontally across each row. Then 100 μL of the bacterial suspension was added to each well, resulting in final concentrations of each drug at 1/4 of their respective mother liquors, and a total reaction volume of 200 μL. After static incubation at 37 °C for 7 days, 10 μL of 0.1% (*w*/*v*) resazurin solution was added to each well, and the plate was incubated at 37 °C for another 24–48 h. Bacterial growth was determined by color change. The fractional inhibitory concentration index (FICI) was calculated as follows [21]:

$$\mathrm{FICI}\;=\;\frac{\mathrm{MIC}\;\mathrm{of}\;\mathrm{thiolutin}\;\mathrm{in}\;\mathrm{combination}}{\mathrm{MIC}\;\mathrm{of}\;\mathrm{thiolutin}\;\mathrm{alone}}+\;\frac{\mathrm{MIC}\;\mathrm{of}\;\mathrm{the}\;\mathrm{partner}\;\mathrm{drug}\;\mathrm{in}\;\mathrm{combination}}{\mathrm{MIC}\;\mathrm{of}\;\mathrm{the}\;\mathrm{partner}\;\mathrm{drug}\;\mathrm{alone}},\;\mathrm{Interactions}\;\mathrm{were}\;\mathrm{classified}\;\mathrm{as}\;\mathrm{synergistic}\;(\leq 0.5),\;\mathrm{additive}\;(>0.5–1.0),\;\mathrm{indifferent}\;(>1.0–4.0),\;\mathrm{or}\;\mathrm{antagonistic}\;(>4.0).$$

#### 2.2.4. In Vitro Cytotoxicity Assay

Before compound exposure, cells were plated in 96-well plates at 1 × 10^4^ cells/well and cultured overnight in an incubator. Each compound was first dissolved in DMSO to prepare a 10 μg/μL stock solution, which was then serially diluted 2-fold with complete medium to yield 10 working concentrations (maximum: 512 μg/mL). Cells were treated with these dilutions for 24 h, with solvent and blank controls included. Following the 24 h treatment, 10 μL of CCK-8 reagent (Solarbio, Beijing, China, Cat. No. CA1210) was added to each well, and the plates were incubated at 37 °C for 1 h. Absorbance at 450 nm was measured to determine cell viability. GraphPad 8.0 (GraphPad Software, San Diego, CA, USA) was used to calculate the IC_50_ for each compound.

## 3. Results and Discussion

### 3.1. Checkerboard Synergy Assay

Through checkerboard assays, we identified that thiolutin acts synergistically with INH, CS, and LZD against MTB H37Ra, as evidenced by FICI values of 0.406, 0.188, and 0.344, respectively (Table 1 and Figure S1). Conversely, no synergistic effect was observed for the combination of thiolutin and PZA (FICI = 1.063).

Upon entering bacterial cells, the disulfide bond of thiolutin is reduced to a dithiol, which chelates divalent metal ions, thereby inhibiting multiple metalloenzymes and disrupting bacterial metal homeostasis. INH inhibits mycolic acid synthesis, CS inhibits peptidoglycan synthesis, and LZD inhibits protein translation [22,23]. The antibacterial activity of these three drugs may be indirectly affected by intracellular metal homeostasis. The metal-chelating activity of thiolutin may reduce bacterial efflux pump activity and interfere with DNA repair or oxidative stress responses [24], thereby enhancing the efficacy of the above drugs against their respective targets, resulting in synergistic effects.

The lack of synergy with PZA may be attributable to the experimental conditions. PZA requires acidic conditions for pyrazinamidase-mediated bioactivation into its antimicrobial metabolite, pyrazinoic acid [25], yet the Middlebrook 7H9 medium used in this study is neutral. The lowest FICI value was observed for the thiolutin-CS combination (0.188), suggesting strong mechanistic complementarity between the two drugs. This result provides a direct rationale for the subsequent design of hybrid molecules in this study.

### 3.2. Design and Synthesis of Hybrid Molecules

Based on the synergistic effects confirmed by the checkerboard assay (Table 1), we covalently linked thiolutin to antitubercular pharmacophores via alkyl linkers to construct hybrid molecules. Inspired by the natural hybrid antibiotic thiomarinol (Figure 1), the present study employed alkyl linkers of 7–10 carbon atoms to covalently connect the DTP scaffold of thiolutin to the pharmacophores of CS, LZD, INH, and 2AP via amide bonds.

**Scheme 1:**
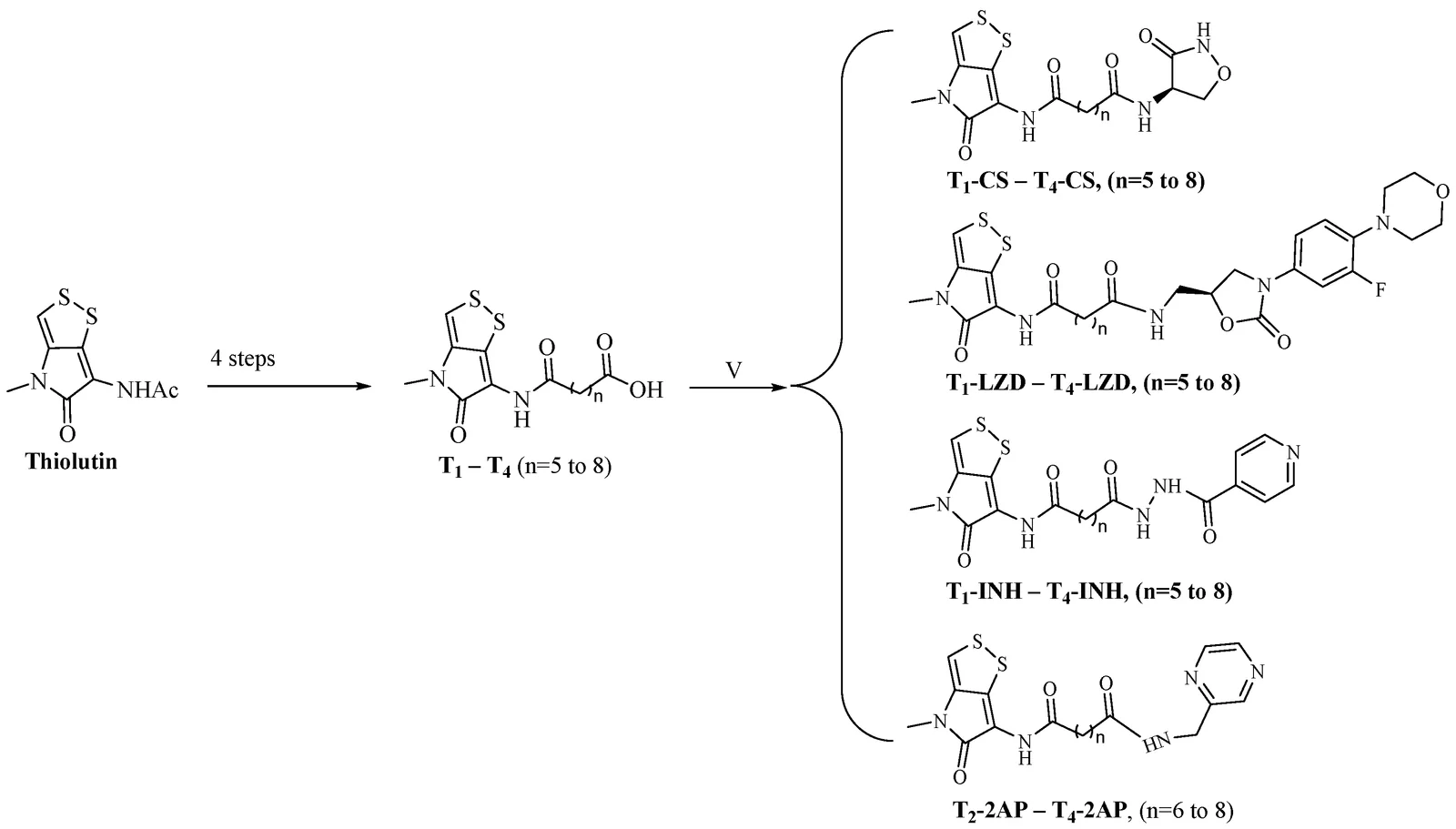
Preparation of thiolutin–antitubercular hybrids. Steps (I)–(IV): see ref. [26] for the four-step conversion of thiolutin into T_1_–T_4_ (*n* = 5–8). Step (V): CH_3_CN, HATU, DIPEA, R, RT, with R = cycloserine (CS), linezolid (LZD), isoniazid (INH), 2-aminopyrazine (2AP). The combination of four n values and four R groups gives 15 compounds (one combination failed to yield the desired product).

As shown in the synthetic route (Scheme 1), because the DTP unit is unstable under alkaline conditions, we adopted a previously established method [26]. Subsequently, the tert-butyl ester protecting group was deprotected via acid-mediated cleavage, furnishing the free carboxylic acid derivatives T_1_–T_4_. Finally, under HATU/DIPEA conditions, each intermediate was coupled with four amine fragments, yielding a total of 15 target hybrid molecules.

### 3.3. Antitubercular Activity, Cytotoxicity, and Structure–Activity Relationship

The antitubercular activity exhibited clear pharmacophore-dependent and linker-length-dependent patterns (Table 2). Regarding the pharmacophore, the activity order was CS series > INH series > 2AP series > LZD series. Among them, the CS hybrids achieved the strongest activity, with MIC values of 2 µg/mL (T_1_-CS) and 1 µg/mL (T_4_-CS) representing the lowest observed values. Although the CS and 2AP hybrids exhibited improved activity over their parent pharmacophores, none of the hybrid compounds showed substantially enhanced antitubercular activity relative to thiolutin (MIC = 1 μg/mL). In contrast, the LZD hybrids showed substantially weaker activity, with MIC values ranging from 64 µg/mL to as high as 256 µg/mL, which may be attributed to their molecular weights exceeding 500, thus violating the “rule of five”.

The influence of linker length varied with the pharmacophore. For the CS series, the best antitubercular activity was observed when the alkyl linker contained 7 carbon atoms or 10 carbon atoms; activity decreased with linker lengths of 8 or 9 carbon atoms. For the INH series, optimal activity was achieved with linkers of 8 or 10 carbon atoms, whereas the LZD series showed a progressive decline in activity as the linker length increased.

All hybrids exhibited IC_50_ values against RAW 264.7 mouse macrophages ranging from 1.02 to 205.9 μg/mL (Table 2). The CS series displayed the lowest overall cytotoxicity, with all IC_50_ values > 40 μg/mL; notably, T_1_-CS had an IC_50_ of 205.9 μg/mL and T_4_-CS had an IC_50_ of 103.2 μg/mL. The selectivity index (SI) was calculated as follows: T_1_-CS gave an SI of 103.0, T_4_-CS gave an SI of 103.2, whereas the parent compound thiolutin had an SI of only 1.4. In other words, T_1_-CS and T_4_-CS maintained comparable antibacterial activity than the parent compound while increasing the selectivity index by approximately 70-fold, thus achieving markedly reduced cytotoxicity. It has been established that both the antibacterial activity and the cytotoxicity of thiolutin originate from the intracellular reductive activation of its disulfide bond—the molecule enters cells as a prodrug and is reduced to a dithiol that chelates divalent metal ions [27]. It is noteworthy that hybridization of thiolutin with D-cycloserine retained the antimycobacterial activity while substantially reducing cytotoxicity. Dithiolopyrrolones such as thiolutin are recognized as prodrugs that are reduced in cells to generate a potent dithiol-containing zinc chelator [27,28]; the reduced form of thiolutin has been shown to inhibit JAMM domain-containing metalloproteases by chelating the catalytic Zn^2+^ ion [29]. On this basis, we hypothesize that different pharmacophore structures may influence the cellular uptake efficiency, subcellular distribution, or the rate of disulfide reduction in the hybrid molecules, thereby reducing eukaryotic cytotoxicity while retaining antibacterial activity. The planar, hydrophobic structure of the DTP core may promote membrane permeation, yet the appended isoxazolidinone ring of the cycloserine pharmacophore may limit non-specific accumulation of the hybrids in eukaryotic cells or decrease the efficiency of disulfide reduction in the mammalian reducing environment [28], whereas the unique cell wall structure and intracellular reducing environment of mycobacteria [30,31]—characterized by reductants such as mycothiol and thioredoxins—may allow more efficient activation of the hybrids. Additionally, the cycloserine moiety—known to inhibit alanine racemase (Alr) and D-alanine:D-alanine ligase (Ddl) in the mycobacterial cell wall biosynthesis pathway [32]—may contribute to dual-targeting or facilitate selective accumulation in mycobacteria. However, these mechanistic hypotheses still require further experimental validation, including studies on cellular uptake, reduction potential measurements, and direct target engagement assays.

After comprehensively assessing biological activity and cytotoxicity, T_1_-CS and T_4_-CS stand out as the most potential anti-TB hybrid molecules identified herein. The hybrid strategy successfully integrates the broad-spectrum metal-chelating action of thiolutin with the targeted cell-wall synthesis inhibition of CS, achieving multi-target synergistic attack at the single-molecule level while markedly reducing the eukaryotic cytotoxicity of the parent compound through structural modification.

## 4. Conclusions

Inspired by the natural hybrid antibiotic thiomarinol and based on synergistic effects, this study designed and synthesized 15 novel thiolutin-antitubercular drug hybrids. Among them, the CS hybrids T_1_-CS and T_4_-CS maintained excellent antitubercular activity while increasing the selectivity index from 1.4 to approximately 103, achieving markedly reduced cytotoxicity. This hybrid strategy provides a valuable molecular template for the development of novel antitubercular agents with multi-target mechanisms and low cytotoxicity. Both T_1_-CS and T_4_-CS can serve as lead compounds warranting further in vivo pharmacodynamic and pharmacokinetic evaluation.

In summary, this study has preliminarily constructed a class of low-toxicity, highly selective thioclase-cycloserine hybrid molecules. However, it should be noted that all antibacterial activity data are from the attenuated strain H37Ra, and its activity against highly virulent strains and clinically resistant strains remains unknown. Furthermore, this study did not investigate the bactericidal effect of the compound in macrophages, and the proposed mechanism hypothesis requires experimental confirmation. Despite these limitations, we observed that while conjugating thioclase with cycloserine did not further enhance its already high antibacterial activity, it did markedly reduce its toxicity to mammalian cells, increasing the selectivity index by approximately 70-fold. Future research will focus on evaluating its activity against resistant strains, validating its intracellular bactericidal ability, and elucidating its mechanism of action.

## Figures and Tables

**Table 1:** In vitro antitubercular synergistic effects of thiolutin combined with INH, CS, LZD, or PZA against *M. tuberculosis* H37Ra (MIC in μg/mL).

Combination	MIC Alone (μg/mL)		MIC in Combination (μg/mL)		FICI ^a^	Interaction
	A	B	A	B		
Thiolutin + INH	1	0.25	0.25 (0.25–0.5)	0.031 (0.031–0.063)	0.406 ± 0.063	Synergistic
Thiolutin + CS	1	64	0.125 (0.063–0.125)	4 (4–8)	0.188 ± 0.051	Synergistic
Thiolutin + LZD	1	1	0.125 (0.125–0.25)	0.25 (0.125–0.25)	0.344 ± 0.063	Synergistic
Thiolutin +PZA	1	256	0.5 (0.5–1)	128 (64–128)	1.063 ± 0.239	Indifferent

^a^ FICI values are expressed as the mean ± standard deviation of no fewer than four independent experiments.

**Table 2:** MIC of hybrid derivatives against *M. tuberculosis* H37Ra and IC_50_ against RAW 264.7 macrophages.

Compounds	*n*	R	H37Ra (MIC, μg/mL) ^a^	RAW 264.7 (IC_50_, μg/mL) ^b^	Selectivity Index (SI) ^c^
T_1_-CS	5	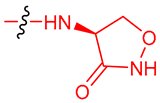	2	205.9 ± 10.05	102.95
T_2_-CS	6	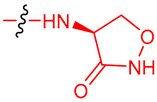	8	45.89 ± 1.875	5.74
T_3_-CS	7	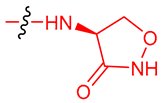	4	56.51 ± 5.64	14.13
T_4_-CS	8	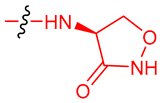	1	103.2 ± 7.85	103.2
CS	/		64	>512	/
T_1_-LZD	5	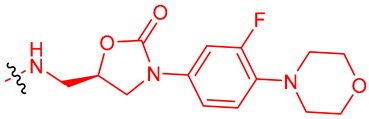	64	12.38 ± 0.635	0.19
T_2_-LZD	6	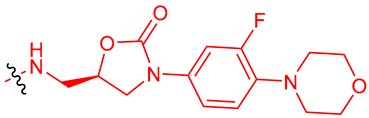	128	5.508 ± 0.88	0.04
T_3_-LZD	7	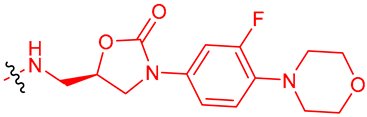	128	5.6 ± 0.18	0.04
T_4_-LZD	8	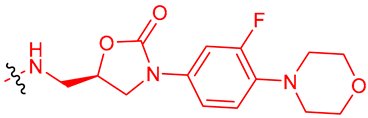	256	3.31 ± 0.56	0.01
LZD	/		1	ND	/
T_1_-INH	5	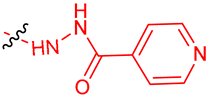	8	11.38 ± 0.38	1.42
T_2_-INH	6	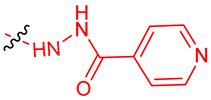	4	11.24 ± 0.69	2.81
T_3_-INH	7	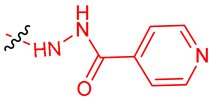	8	5.03 ± 0.19	0.63
T_4_-INH	8	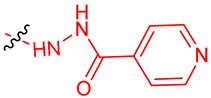	4	5.04 ± 0.22	1.26
INH	/		0.25	ND	/
T_2_-2AP	6	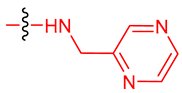	32	1.846 ± 0.345	0.06
T_3_-2AP	7	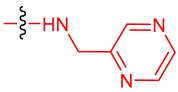	16	3.674 ± 0.289	0.23
T_4_-2AP	8	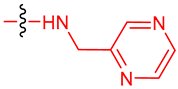	64	1.024 ± 0.054	0.02
PZA	/		256	ND	/
Thiolutin	/	/	1	1.37 ± 0.241	1.37

^a^ MIC values were determined from at least three independent experiments. ^b^ The IC_50_ is presented as the mean ± standard deviation of at least three independent assays. ^c^ SI: IC_50_/MIC for H37Ra. ND: Not Determined.

## Data Availability

The original contributions presented in this study are included in the article/Supplementary Material. Further inquiries can be directed to the corresponding authors.

## Supplementary Materials

The following supporting information can be downloaded at https://www.mdpi.com/article/10.3390/microorganisms14081670/s1, All target compounds’ ^1^H NMR, ^13^C NMR and high-resolution mass spectra, along with full characterization data of T_2_-CS, T_3_-CS, T_4_-CS, T_2_-LZD, T_3_-LZD, T_4_-LZD, T_2_-INH, T_3_-INH, T_4_-INH, T_3_-2AP and T_4_-2AP. Figure S1. Checkerboard synergy assay of thiolutin combined with INH, CS, LZD, and PZA against *M. tuberculosis* H37Ra.

## Abbreviations

The following abbreviations are used in this manuscript:

TB	Tuberculosis
DTP	Dithiolopyrrolone
MTB	*Mycobacterium tuberculosis*
IleRS	Isoleucyl-tRNA synthetase
INH	Isoniazid
CS	Cycloserine
LZD	Linezolid
PZA	Pyrazinamide
FICI	Fractional inhibitory concentration index
2AP	2-aminopyrazine
