# Supplementary material for: Design, Synthesis, and Antitubercular Activity of Thiolutin–Cycloserine Hybrids: Reducing Cytotoxicity
Source: Microorganisms. 2026 Jul 30;14(8):1670. doi: 10.3390/microorganisms14081670 (PMC13515660; doi:10.3390/microorganisms14081670)
Supplement: Supplementary file 1 [file microorganisms-14-01670-s001.zip › microorganisms-4371938-supplementary.pdf]

Design, Synthesis, and Antitubercular Activity of Thiolutin–Cycloserine Hybrids: Reducing Cytotoxicity

Supporting information

1. NMR data of the hybrid compounds 2
Figure S1. Checkerboard synergy assay of thiolutin combined with INH, CS, LZD, and PZA against *M tuberculosis* H37Ra. 5
2. Spectral data (Copies of the NMR and HRMS for Compounds) 6
3. ESI mass spectra and HPLC chromatograms of all compounds 22

1. NMR data of the hybrid compounds

1.1

(*R*)-N[1]-(4-methyl-5-oxo-4,5-dihydro-[1,2]dithiolo[4,3-*b*]pyrrol-6-yl)-N[8]-(3-oxoisoxazolidin-4-yl) heptanediamide **(T_2-CS)**. As yellow solid (43%). ^{1}H NMR (500 MHz, DMSO-*d6*) δ 11.49 (s, 1H), 9.94 (s, 1H), 8.37 (d, *J* = 7.5 Hz, 1H), 7.33 (s, 1H), 4.73 (s, 1H), 4.50 (t, *J* = 8.3 Hz, 1H), 3.88 (t, *J* = 9.1 Hz, 1H), 3.25 (s, 3H), 2.33 (t, *J* = 7.3 Hz, 2H), 2.11 (t, *J* = 7.3 Hz, 2H), 1.56 – 1.44 (m, 4H), 1.25 (m, 4H). ^{13}C NMR (125 MHz, DMSO-*d6*) δ 172.60, 171.84, 166.21, 135.98, 132.42, 114.78, 110.90, 72.46, 51.28, 35.01, 34.64, 28.36, 28.31, 27.54, 25.01, 24.98. Based on HRMS (ESI) for $C_{17}H_{23}N_4O_5S_2$ $[M + H]^+$, the calculated mass was 427.1110, and the found mass was 427.1043. Evaluation by HPLC gave a purity of 98.0% and a retention time of 12.44 min, using a mobile phase consisting of 5% CH_3CN in 95% H_2O.

1.2

(*R*)-N[1]-(4-methyl-5-oxo-4,5-dihydro-[1,2]dithiolo[4,3-*b*]pyrrol-6-yl)-N[9]-(3-oxoisoxazolidin-4-yl) heptanediamide **(T_3-CS)**. As yellow solid (41%). ^{1}H NMR (500 MHz, DMSO-*d6*) δ 11.48 (s, 1H), 9.94 (s, 1H), 8.36 (d, *J* = 7.6 Hz, 1H), 7.33 (s, 1H), 4.86 – 4.63 (m, 1H), 4.49 (t, *J* = 8.4 Hz, 1H), 3.88 (t, *J* = 9.1 Hz, 1H), 3.25 (s, 3H), 2.33 (t, *J* = 7.3 Hz, 2H), 2.11 (t, *J* = 7.2 Hz, 2H), 1.54 – 1.43 (m, 4H), 1.25 (m, 6H). ^{13}C NMR (125 MHz, DMSO-*d6*) δ 172.63, 171.87, 166.21, 135.98, 132.43, 114.78, 110.91, 72.47, 52.99, 35.02, 34.65, 28.50, 28.46, 27.54, 25.11, 25.06. Based on HRMS (ESI) for $C_{18}H_{25}N_4O_5S_2$ $[M + H]^+$, the calculated mass was 441.1266, and the found mass was 441.1194. Evaluation by HPLC gave a purity of 95.8% and a retention time of 13.838 min, using a mobile phase consisting of 5% CH_3CN in 95% H_2O.

1.3

(*R*)-N[1]-(4-methyl-5-oxo-4,5-dihydro-[1,2]dithiolo[4,3-*b*]pyrrol-6-yl)-N[10]-(3-oxoisoxazolidin-4-yl)heptanediamide **(T_4-CS)**. As yellow solid (50%). ^{1}H NMR (500 MHz, DMSO-*d6*) δ 11.49 (s, 1H), 9.94 (s, 1H), 8.36 (d, *J* = 7.8 Hz, 1H), 7.34 (s, 1H), 4.74 (m, 1H), 4.50 (t, *J* = 8.3 Hz, 1H), 3.88 (dd, *J* = 9.8, 8.4 Hz, 1H), 3.25 (s, 3H), 2.34 (t, *J* = 7.4 Hz, 2H), 2.17 – 2.08 (t, *J* = 7.3 Hz, 2H), 1.50 (m, 4H), 1.25 (m, 8H). ^{13}C NMR (125 MHz, DMSO-*d6*) δ 172.63, 171.87, 166.21, 135.98, 132.42, 114.78, 110.90, 72.05, 50.84, 35.02, 34.67, 28.68, 28.66, 28.57, 27.54, 25.12, 25.08. Based on HRMS (ESI) for $C_{19}H_{27}N_4O_5S_2$ $[M + H]^+$, the calculated mass was 455.1423, and the found mass was 455.1417. Evaluation by HPLC gave a purity of 96.6% and a retention time of 15.378 min, using a mobile phase consisting of 5% CH_3CN in 95% H_2O.

1.4

N[1]-(((*R*)-3-(3-fluoro-4-morpholinophenyl)-2-oxooxazolidin-5-yl)methyl)-N[3]-(4-methyl-5-oxo-4,5-dihydro-[1,2]dithiolo[4,3-*b*]pyrrol-6-yl)-2-pentylmalonamide **(T_2-LZD)**. As yellow solid (65%). ^{1}H NMR (500 MHz, DMSO-*d6*) δ 9.92 (s, 1H), 8.19 (t, *J* = 5.6 Hz, 1H), 7.51 – 7.45 (m, 1H), 7.33 (s, 1H), 7.15 (d, *J* = 8.1 Hz, 1H), 7.04 (t, *J* = 9.3 Hz, 1H), 4.72 (dd, *J* = 8.3, 4.5 Hz, 1H), 4.07 (t, *J* = 9.0 Hz, 1H), 3.69 (d, *J* = 17.1 Hz, 5H), 3.45 (dt, *J* = 11.4, 5.6 Hz, 2H), 3.24 (s, 3H), 2.94 (m, 4H), 2.30 (t, *J* = 7.3 Hz, 2H), 2.12 (t, *J* = 8.5 Hz, 2H), 1.47 – 1.38 (m, 4H), 1.17 (m, 4H). ^{13}C NMR (125 MHz, DMSO-*d6*) δ 173.12, 171.81, 166.20, 155.08, 154.08, 153.34, 148.74, 135.98, 135.94, 135.48,

135.44, 133.49, 133.40, 132.40, 122.50, 119.23, 114.79, 113.96, 111.15, 110.89, 106.63, 106.42, 71.58, 66.17, 50.72, 47.18, 41.22, 35.26, 34.63, 28.36, 28.31, 27.53, 25.22, 24.89.Based on HRMS (ESI) for $C_{28}H_{35}FN_5O_6S_2$ $[M + H]^+$, the calculated mass was 620.2013, and the found mass was 620.1905. Evaluation by HPLC gave a purity of 96.3% and a retention time of 19.373 min, using a mobile phase consisting of 5% CH_3CN in 95% H_2O.

1.5

N[1]-(((*R*)-3-(3-fluoro-4-morpholinophenyl)-2-oxooxazolidin-5-yl)methyl)-2-hexyl-N[3]-(4-methyl-5-oxo-4,5-dihydro-[1,2]dithiolo[4,3-*b*]pyrrol-6-yl)malonamide **(T_3-LZD)**. As yellow solid (77％).^{1}H NMR (500 MHz, DMSO-*d6*) δ 9.93 (s, 1H), 8.20 (t, *J* = 5.7 Hz, 1H), 7.50 – 7.44 (m, 1H), 7.33 (s, 1H), 7.15 (d, *J* = 8.6 Hz, 1H), 7.05 (t, *J* = 9.3 Hz, 1H), 4.72 (dd, *J* = 8.5, 4.5 Hz, 1H), 4.07 (t, *J* = 9.0 Hz, 1H), 3.75 – 3.70 (m, 4H), 3.70 – 3.66 (m, 1H), 3.46 (dt, *J* = 11.6, 6.0 Hz, 2H), 3.24 (s, 3H), 3.01 – 2.92 (m, 4H), 2.31 (t, *J* = 7.3 Hz, 2H), 2.12 – 2.03 (m, 2H), 1.43 (m, 4H), 1.16 (m, 6H).^{13}C NMR (125 MHz, DMSO-*d6*) δ 173.19, 171.86, 166.21, 154.08, 135.98, 135.59, 135.44, 133.50, 133.47, 132.41, 119.25, 119.22, 114.79, 113.92, 110.90, 106.60, 106.39, 71.61, 66.18, 50.74, 50.72, 47.14, 41.19, 35.30, 34.65, 28.47, 28.42, 28.39, 27.54, 25.35, 25.04.Based on HRMS (ESI) for $C_{29}H_{37}FN_5O_6S_2$ $[M + H]^+$, the calculated mass was 634.2169, and the found mass was 634.2180. Evaluation by HPLC gave a purity of 95.1% and a retention time of 20.566 min, using a mobile phase consisting of 5% CH_3CN in 95% H_2O.

1.6

N[1]-(((*R*)-3-(3-fluoro-4-morpholinophenyl)-2-oxooxazolidin-5-yl)methyl)-2-heptyl-N[3]-(4-methyl-5-oxo-4,5-dihydro-[1,2]dithiolo[4,3-*b*]pyrrol-6-yl)malonamide **(T_4-LZD)**. As yellow solid (82％).^{1}H NMR (500 MHz, DMSO-*d6*) δ 9.93 (s, 1H), 8.19 (t, *J* = 5.8 Hz, 1H), 7.48 (dd, *J* = 15.0, 2.3 Hz, 1H), 7.33 (s, 1H), 7.18 – 7.12 (m, 1H), 7.04 (t, *J* = 9.4 Hz, 1H), 4.72 (dd, *J* = 8.6, 4.5 Hz, 1H), 4.07 (t, *J* = 9.0 Hz, 1H), 3.70 (dt, *J* = 18.1, 5.2 Hz, 5H), 3.50 – 3.43 (m, 1H), 3.36 (s, 2H), 3.24 (s, 3H), 2.98 – 2.92 (m, 4H), 2.32 (t, *J* = 7.3 Hz, 2H), 2.06 (t, *J* = 7.1 Hz, 2H), 1.51 – 1.37 (m, 4H), 1.14 (m, 8H).^{13}C NMR (125 MHz, DMSO-*d6*) δ 173.18, 171.85, 166.20, 155.55, 154.07, 153.62, 135.98, 135.49, 135.42, 133.49, 133.41, 132.39, 119.23, 119.19, 114.79, 113.91, 110.88, 106.59, 106.38, 71.61, 66.16, 50.73, 50.71, 47.11, 41.14, 35.28, 34.67, 28.65, 28.58, 28.55, 27.53, 25.34, 25.05.Based on HRMS (ESI) for $C_{30}H_{39}FN_5O_6S_2$ $[M + H]^+$, the calculated mass was 648.2326, and the found mass was 648.2218. Evaluation by HPLC gave a purity of 96.1% and a retention time of 21.873 min, using a mobile phase consisting of 5% CH_3CN in 95% H_2O.

1.7

2-(2-isonicotinoylhydrazine-1-carbonyl)-*N*-(4-methyl-5-oxo-4,5-dihydro-[1,2]dithiolo[4,3-*b*]pyrrol-6-yl)heptanamide **(T_2-INH)**. As yellow solid (50％).^{1}H NMR (500 MHz, DMSO-*d6*) δ 10.65 (s, 1H), 9.91 (d, *J* = 2.3 Hz, 2H), 8.76 (m, 2H), 7.77 (m, 2H), 7.34 (s, 1H), 3.25 (s, 3H), 2.35 (t, *J* = 7.4 Hz, 2H), 2.18 (t, *J* = 7.3 Hz, 2H), 1.59 – 1.48 (m, 4H), 1.30 (m, 4H).^{13}C NMR (125 MHz, DMSO-*d6*) δ 171.84, 171.52, 166.21, 163.94, 150.42, 139.51, 135.98, 132.44, 121.33, 114.78, 110.92, 34.66, 33.25, 28.36, 28.30, 27.54, 25.00, 24.97.Based on HRMS (ESI) for $C_{20}H_{24}N_5O_4S_2$ $[M + H]^+$, the calculated mass was 462.1270, and the found mass was 462.1179. Evaluation by HPLC gave a purity of 95.7% and a retention time of 14.024 min, using a mobile phase consisting of 5% CH_3CN in 95% H_2O.

1.8

2-(2-isonicotinoylhydrazine-1-carbonyl)-*N*-(4-methyl-5-oxo-4,5-dihydro-[1,2]dithiolo[4,3-*b*] pyrrol-6-yl)octanamide **(T_3-INH)**. As yellow solid (43%).^{1}H NMR (500 MHz, DMSO-*d6*) δ 10.60 (s, 1H), 9.95 (d, *J* = 7.7 Hz, 2H), 8.75 (d, *J* = 5.3 Hz, 2H), 7.76 (d, *J* = 5.8 Hz, 2H), 7.33 (s, 1H), 3.25 (s, 3H), 2.35 (t, *J* = 7.3 Hz, 2H), 2.19 (t, *J* = 7.3 Hz, 2H), 1.53 (m, 4H), 1.29 (m, 6H).^{13}C NMR (125 MHz, DMSO-*d6*) ^{13}C NMR δ 171.88, 171.54, 166.21, 163.93, 150.42, 139.51, 135.98, 132.44, 121.33, 114.78, 110.93, 34.66, 33.26, 28.52, 28.50, 28.42, 27.54, 25.07.Based on HRMS (ESI) for $C_{21}H_{26}N_5O_4S_2$ $[M + H]^+$, the calculated mass was 476.1426, and the found mass was 476.1356. Evaluation by HPLC gave a purity of 96.1% and a retention time of 15.426 min, using a mobile phase consisting of 5% CH_3CN in 95% H_2O.

1.9

2-(2-isonicotinoylhydrazine-1-carbonyl)-*N*-(4-methyl-5-oxo-4,5-dihydro-[1,2]dithiolo[4,3-*b*] pyrrol-6-yl)nonanamide **(T_4-INH)**. As yellow solid (59%).^{1}H NMR (500 MHz, DMSO-*d6*) δ 10.60 (s, 1H), 9.95 (d, *J* = 6.3 Hz, 2H), 8.75 (d, *J* = 4.9 Hz, 2H), 7.76 (d, *J* = 5.0 Hz, 2H), 7.33 (s, 1H), 3.25 (s, 3H), 2.34 (t, *J* = 7.3 Hz, 2H), 2.18 (t, *J* = 7.3 Hz, 2H), 1.53 (m, 4H), 1.27 (m, 8H).^{13}C NMR (125 MHz, DMSO-*d6*) δ 171.87, 171.53, 166.21, 163.93, 150.42, 139.51, 135.98, 132.42, 121.33, 114.78, 110.91, 34.68, 33.26, 28.70, 28.59, 28.55, 27.54, 25.08. Based on HRMS (ESI) for $C_{22}H_{28}N_5O_4S_2$ $[M + H]^+$, the calculated mass was 490.1582, and the found mass was 490.1521. Evaluation by HPLC gave a purity of 95.3% and a retention time of 16.975 min, using a mobile phase consisting of 5% CH_3CN in 95% H_2O.

1.10

2-hexyl-N^1-(4-methyl-5-oxo-4,5-dihydro-[1,2]dithiolo[4,3-*b*]pyrrol-6-yl)-N^3-(p yrazin-2-ylmethyl)malonamide **(T3-PZA)**. As yellow solid (75%).^{1}H NMR (500 MHz, DMSO-*d6*) δ 9.94 (s, 1H), 8.59 – 8.50 (m, 3H), 8.47 (t, *J* = 5.4 Hz, 1H), 7.33 (s, 1H), 4.39 (d, *J* = 5.8 Hz, 2H), 3.25 (s, 3H), 2.33 (t, *J* = 7.3 Hz, 2H), 2.15 (t, *J* = 7.3 Hz, 2H), 1.50 (m, 4H), 1.25 (m, 6H). ^{13}C NMR (125 MHz, DMSO-*d6*) δ 172.58, 171.86, 166.21, 154.46, 143.90, 143.27, 143.11, 135.98, 132.43, 114.78, 110.92, 42.22, 35.19, 34.65, 28.52, 28.49, 27.54, 25.21, 25.05.Based on HRMS (ESI) for $C_{20}H_{26}N_5O_3S_2$ $[M + H]^+$, the calculated mass was 448.1477, and the found mass was 448.1461. Evaluation by HPLC gave a purity of 97.4% and a retention time of 16.326 min, using a mobile phase consisting of 5% CH_3CN in 95% H_2O.

1.11

2-heptyl-N^1-(4-methyl-5-oxo-4,5-dihydro-[1,2]dithiolo[4,3-*b*]pyrrol-6-yl)-N^3-(pyrazin-2-ylmethyl)malonamidee **(T4-PZA)**. As yellow solid (66%).^{1}H NMR (500 MHz, DMSO-*d6*) δ 9.94 (s, 1H), 8.59 – 8.50 (m, 3H), 8.47 (t, *J* = 6.0 Hz, 1H), 7.33 (s, 1H), 4.38 (d, *J* = 5.9 Hz, 2H), 3.25 (s, 3H), 2.33 (t, *J* = 7.4 Hz, 2H), 2.15 (t, *J* = 7.4 Hz, 2H), 1.51 (m, 4H), 1.24 (m, 8H). ^{13}C NMR (125 MHz, DMSO-*d6*) δ 172.56, 171.85, 166.19, 154.45, 143.88, 143.26, 143.11, 135.97, 132.40,

114.77, 110.89, 42.22, 35.19, 34.67, 28.68, 28.65, 28.63, 28.56, 27.53, 25.20, 25.08. Based on HRMS (ESI) for $C_{21}H_{28}N_5O_3S_2$ $[M + H]^+$, the calculated mass was 462.1634, and the found mass was 462.1636. Evaluation by HPLC gave a purity of 98.0% and a retention time of 17.943 min, using a mobile phase consisting of 5% CH_3CN in 95% H_2O.

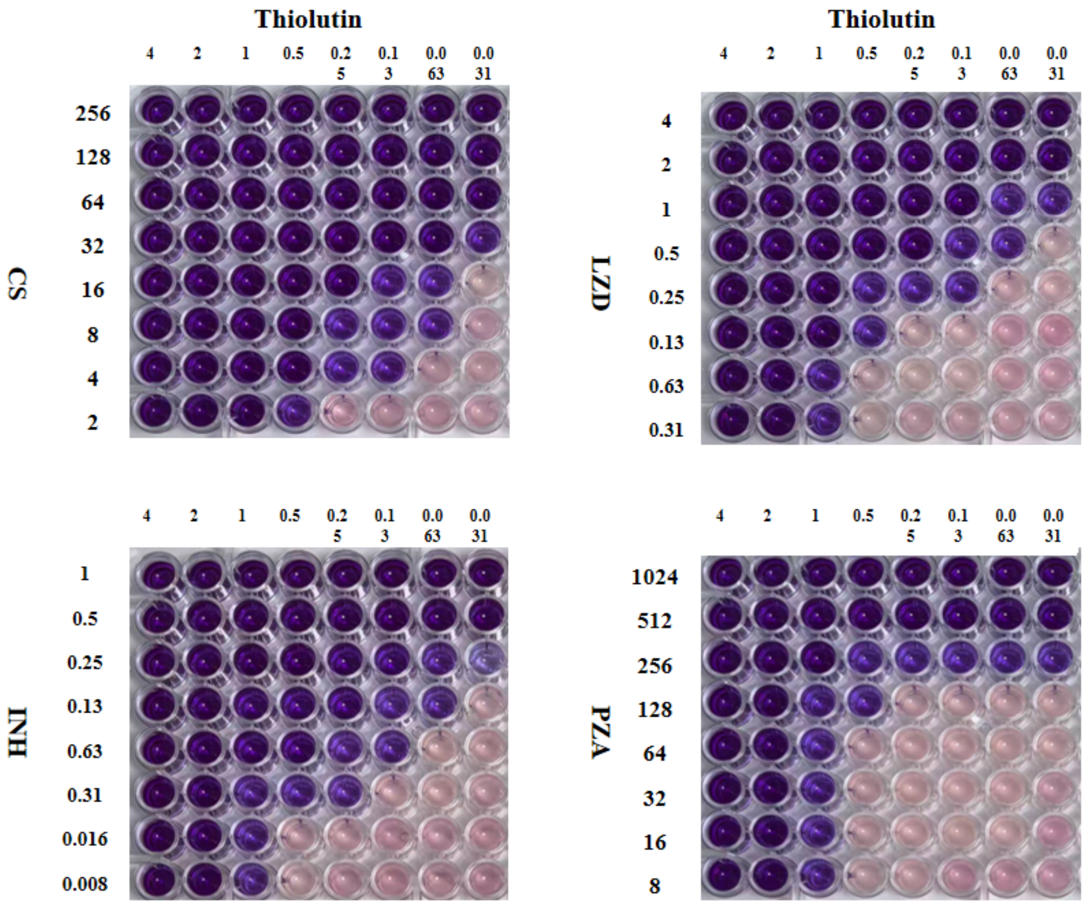

Figure S1. Checkerboard synergy assay of thiolutin combined with INH, CS, LZD, and PZA against *M tuberculosis* H37Ra.

2. Spectral data (Copies of the NMR and HRMS for Compounds)

2.1Compound **T_1-CS**

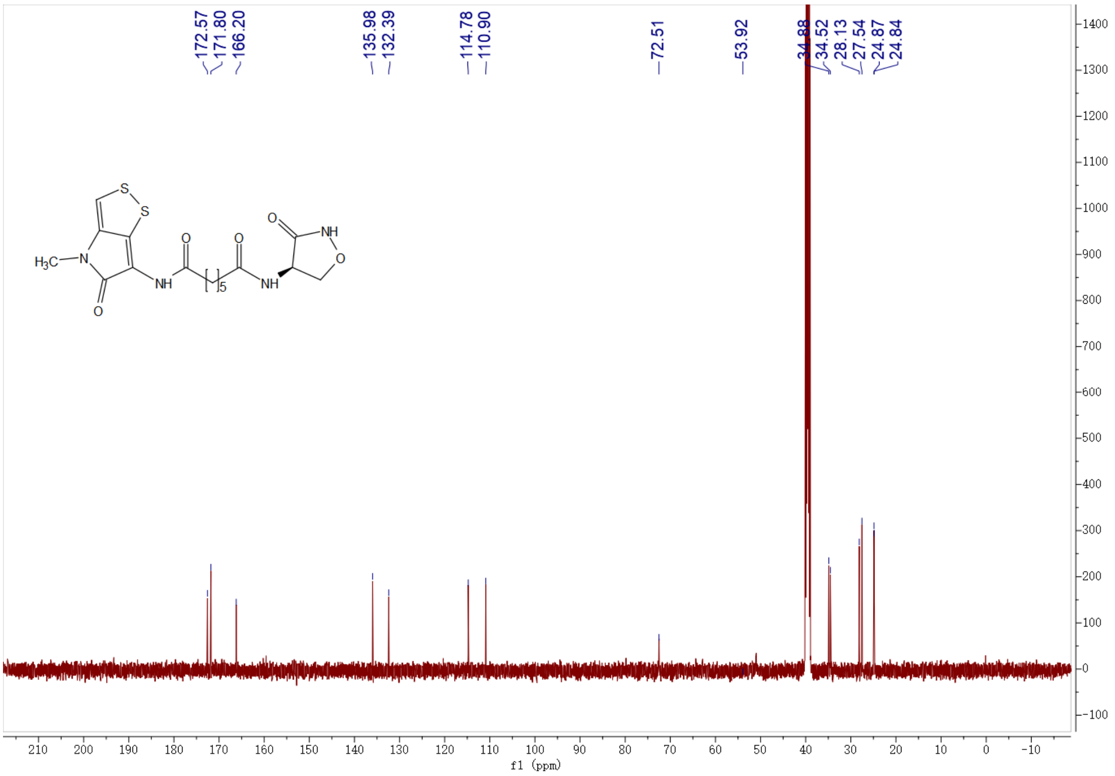

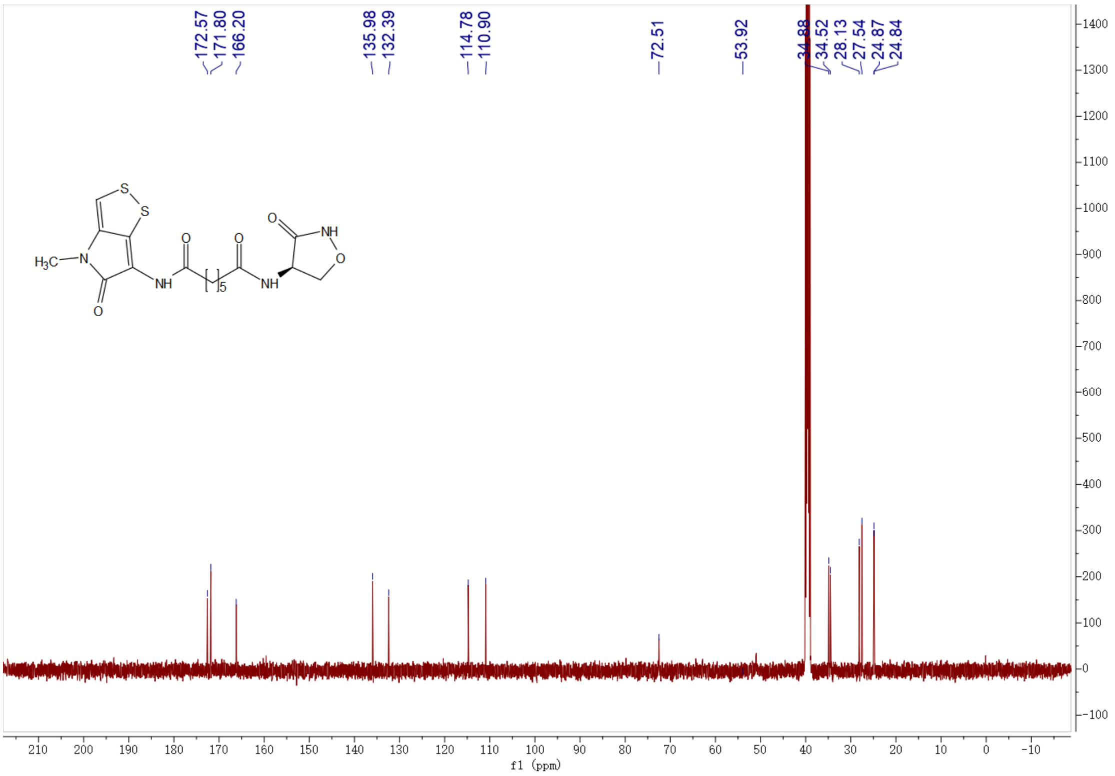

2.2 Compound $\mathbf{T_2}$-CS

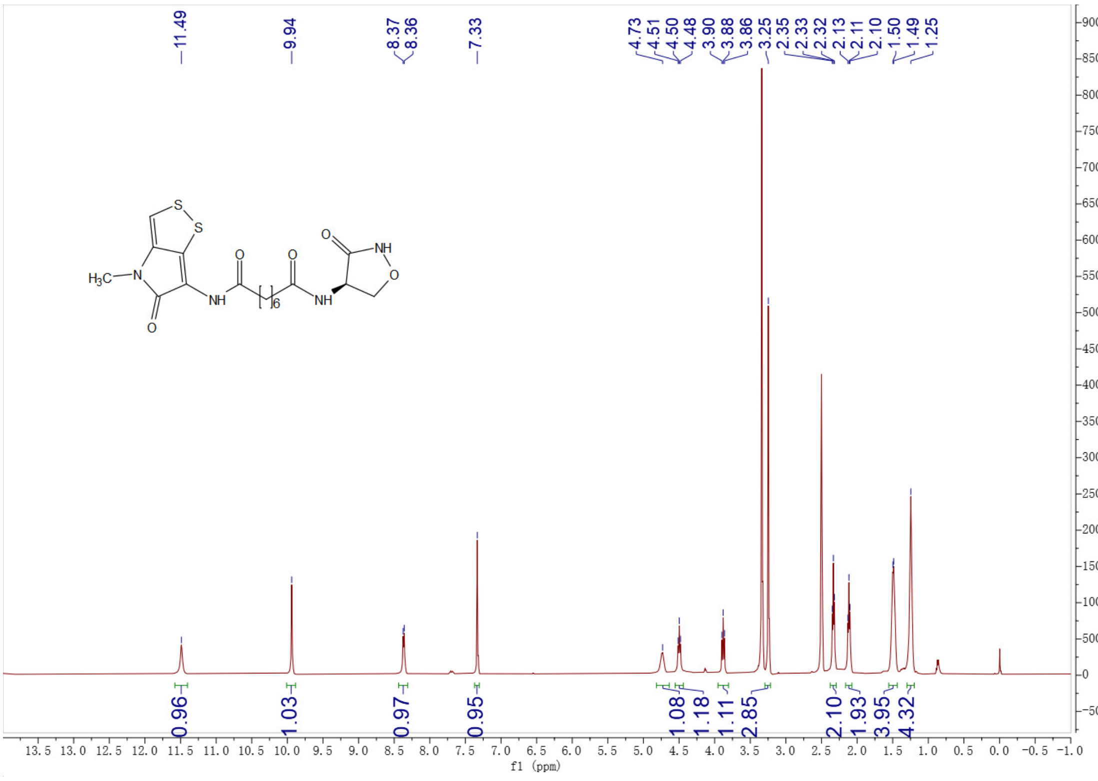

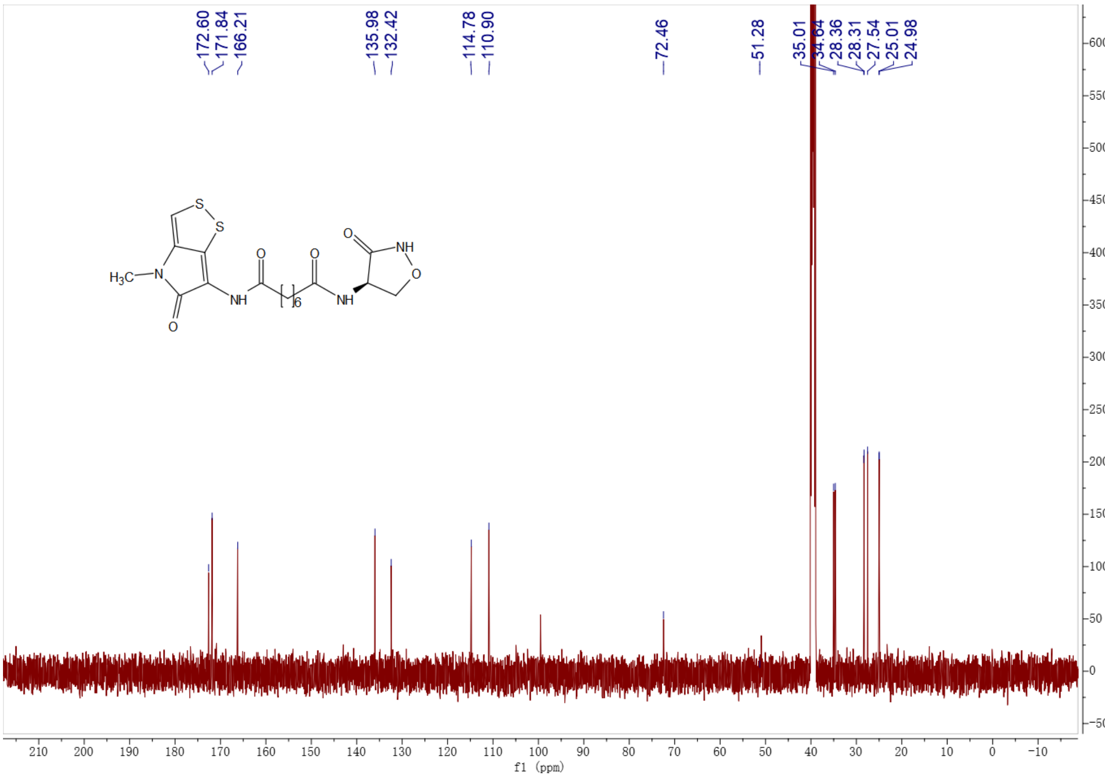

2.3 Compound $\mathbf{T_3}$-**CS**

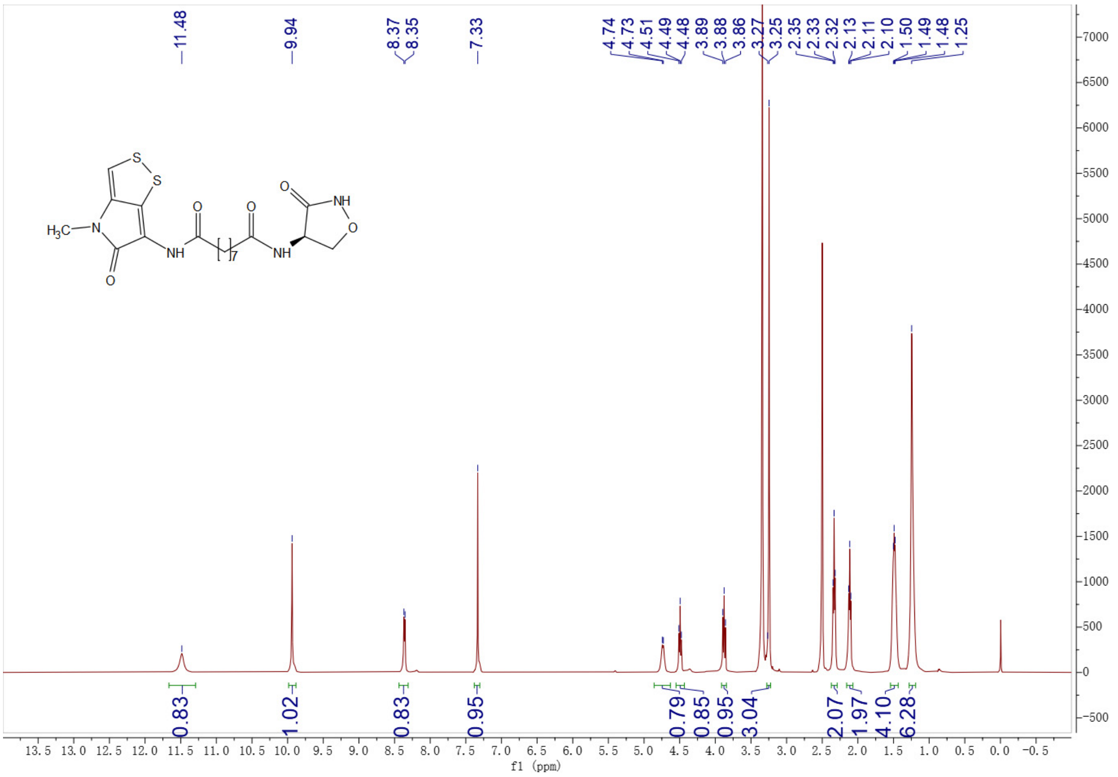

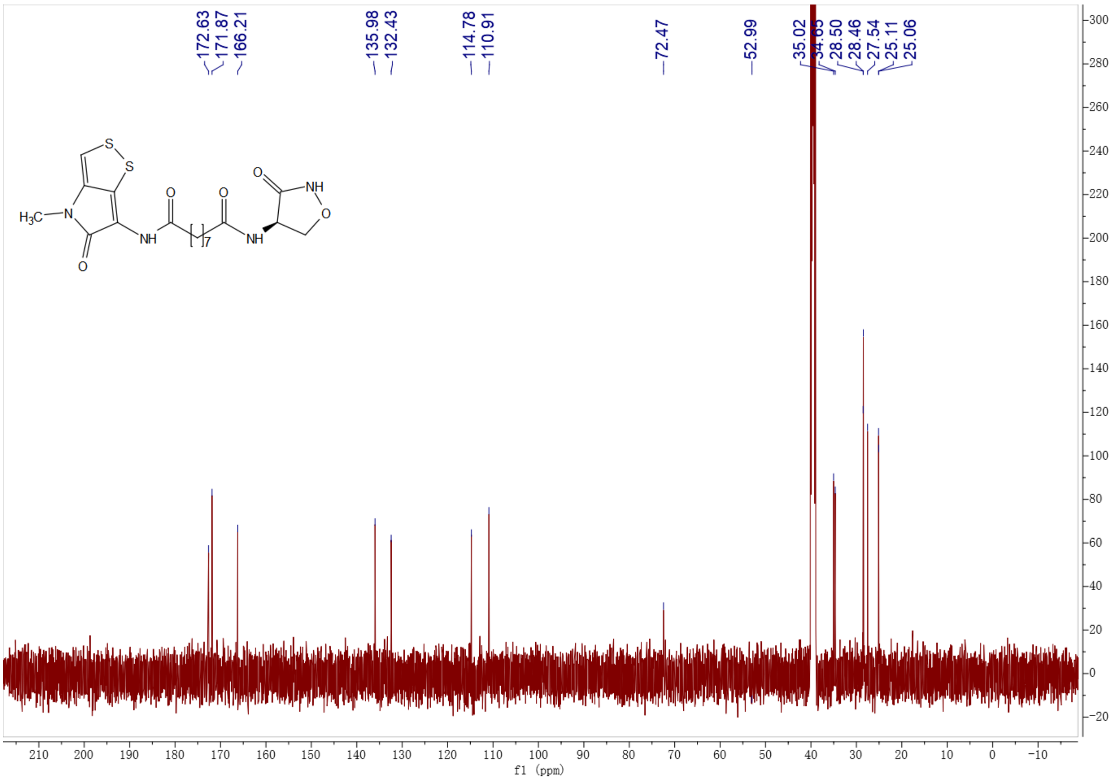

2.4 Compound **T_4-CS**

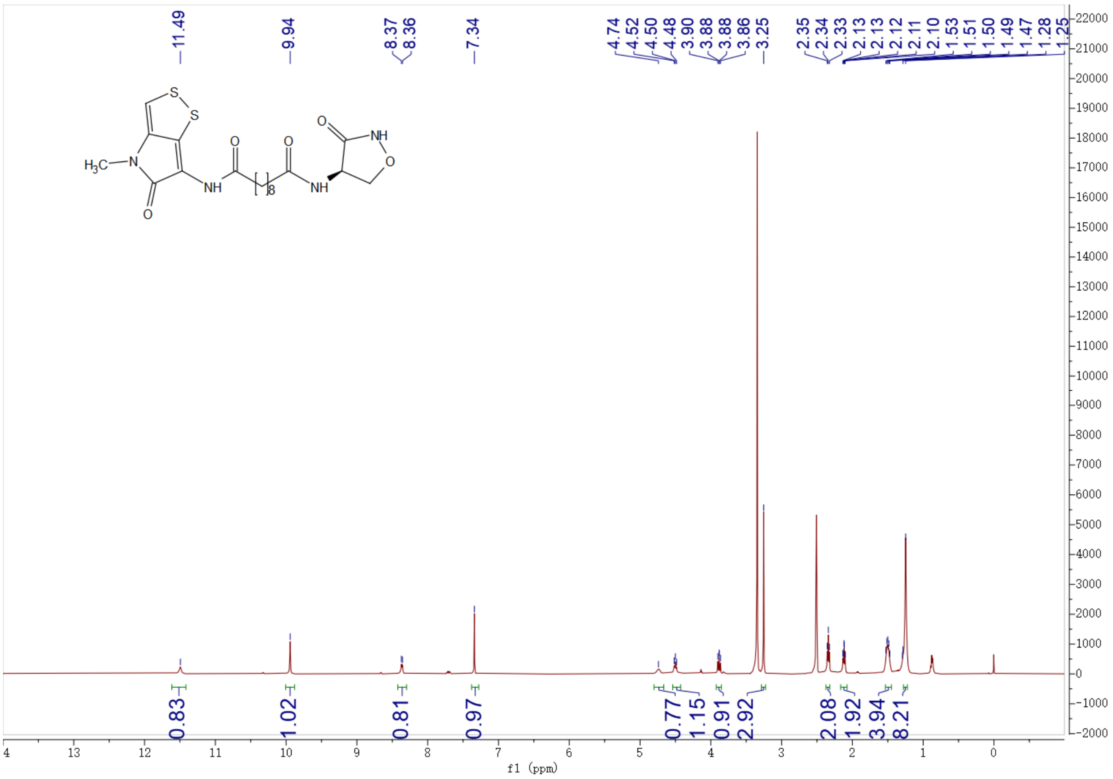

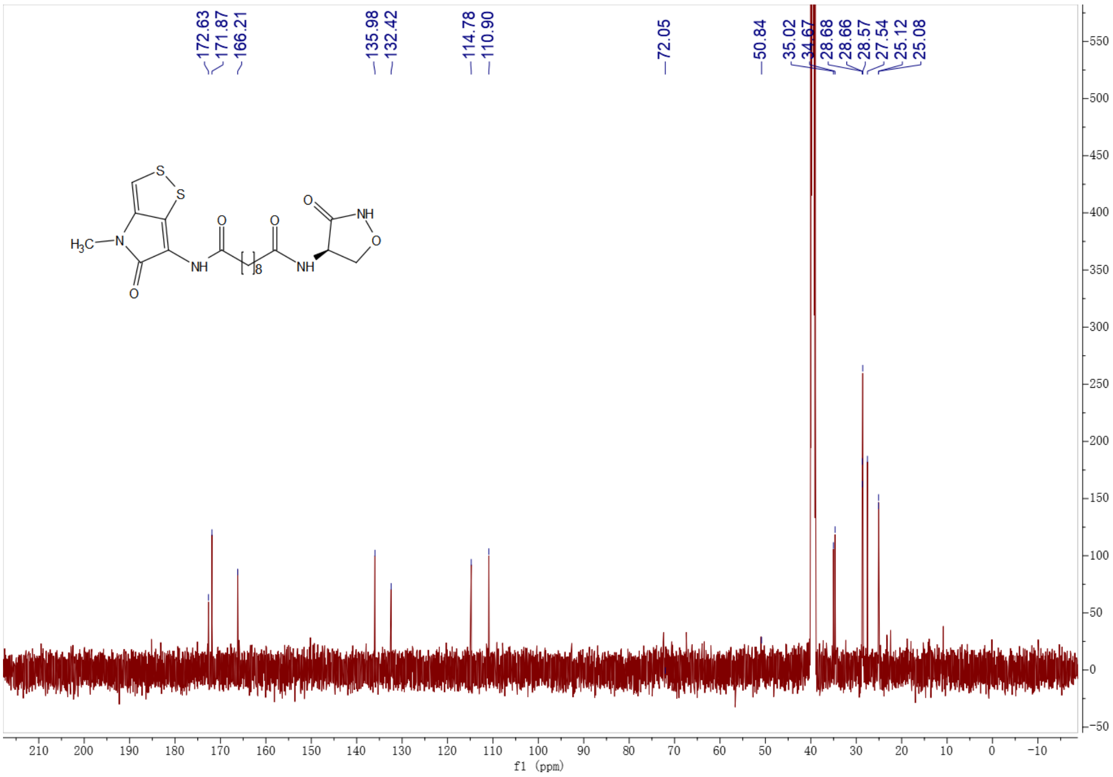

2.5 Compound **T₁-LZD**

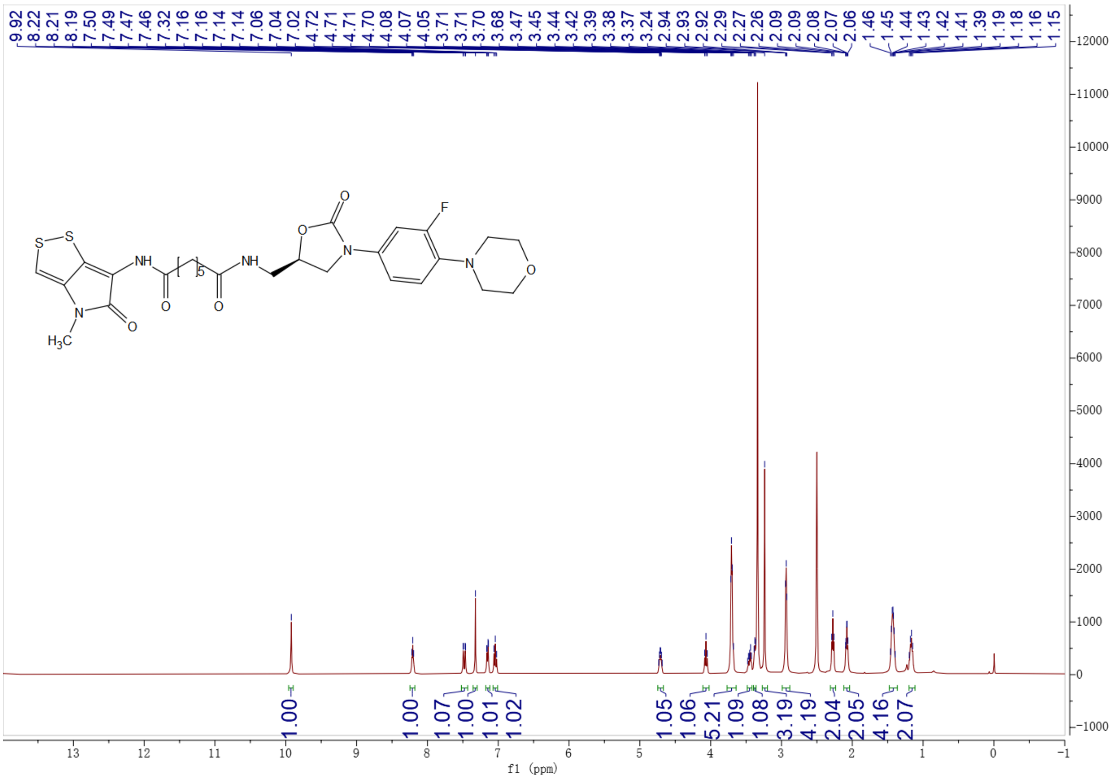

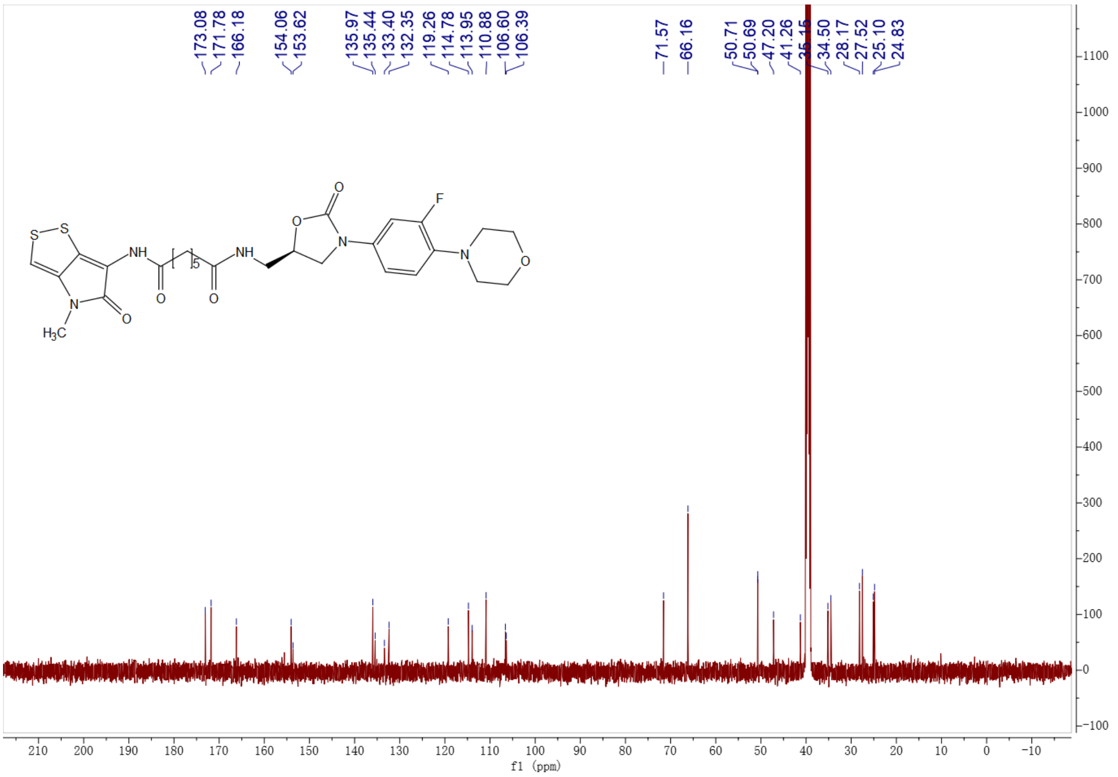

2.6 Compound T_2-LZD

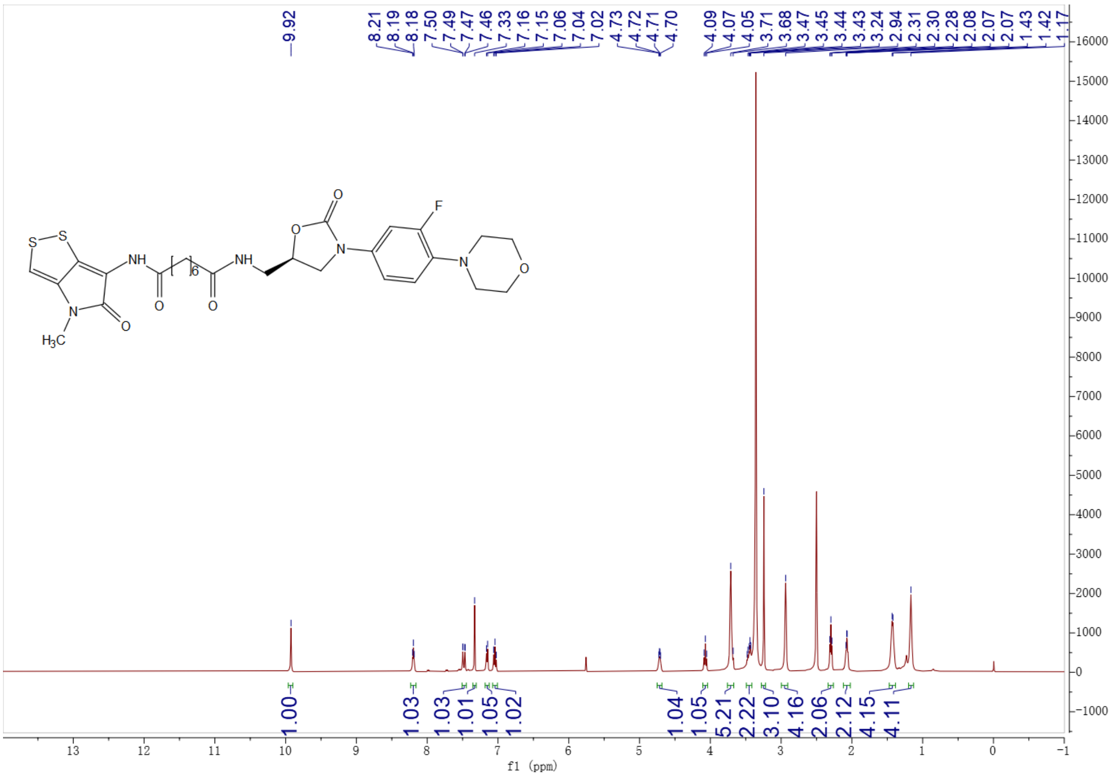

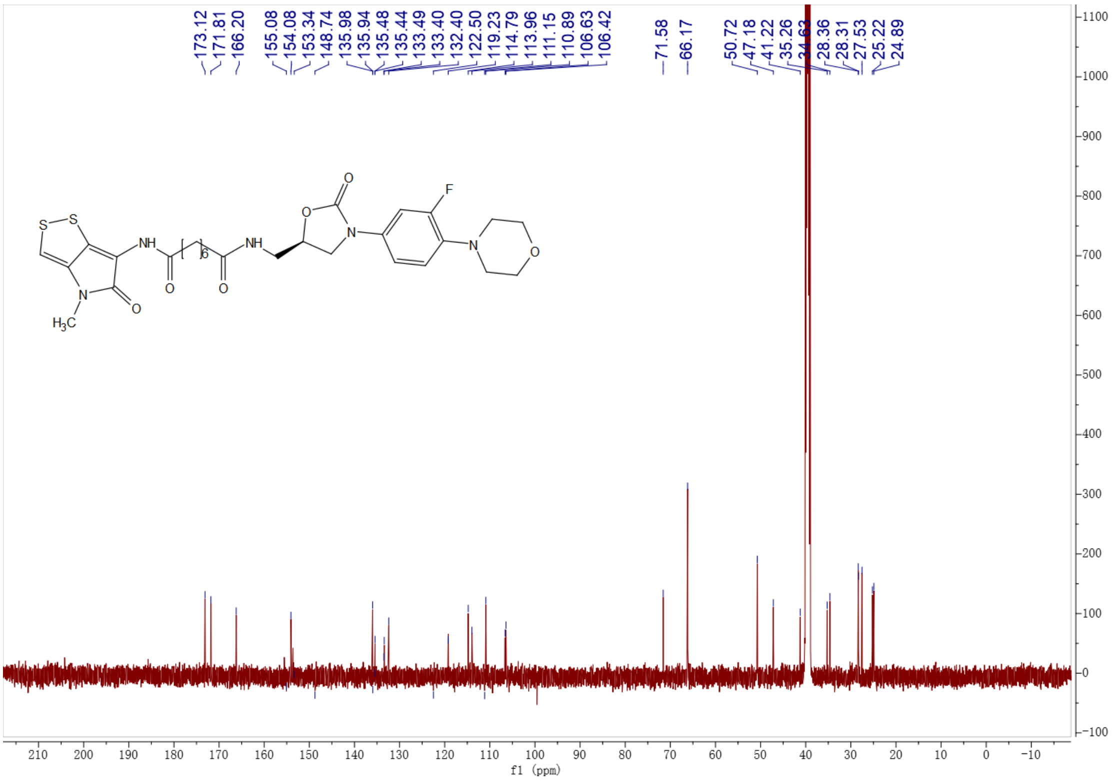

2.7 Compound **T_3-LZD**

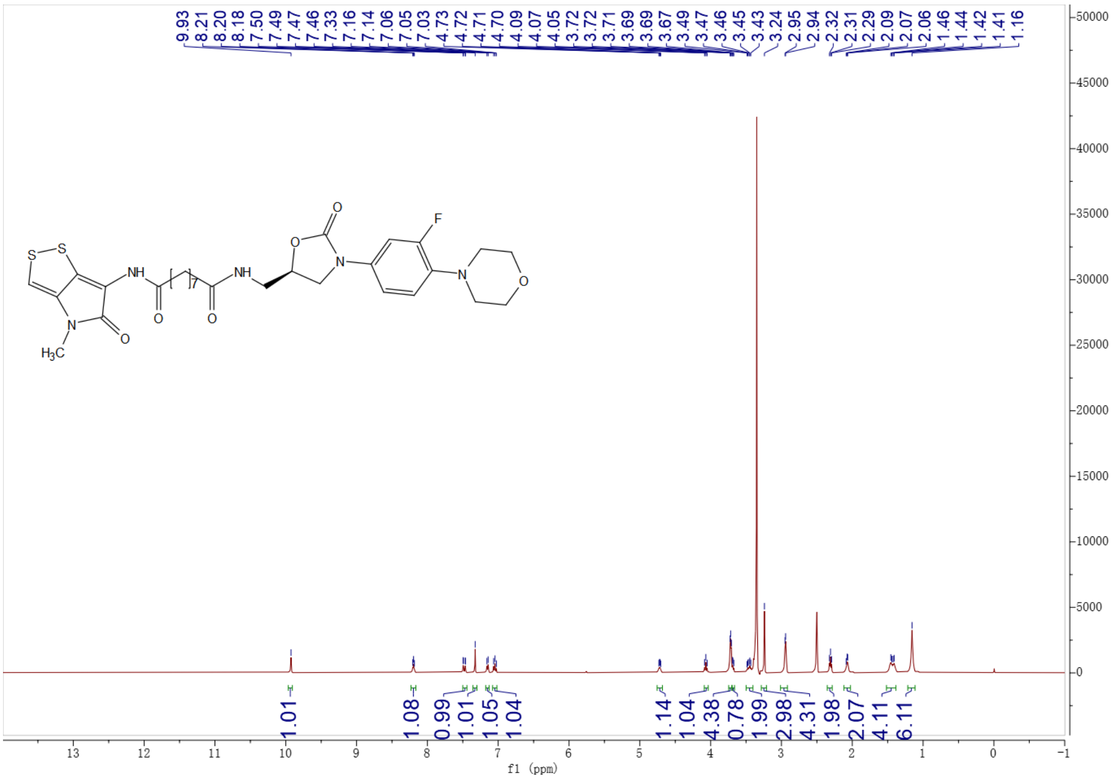

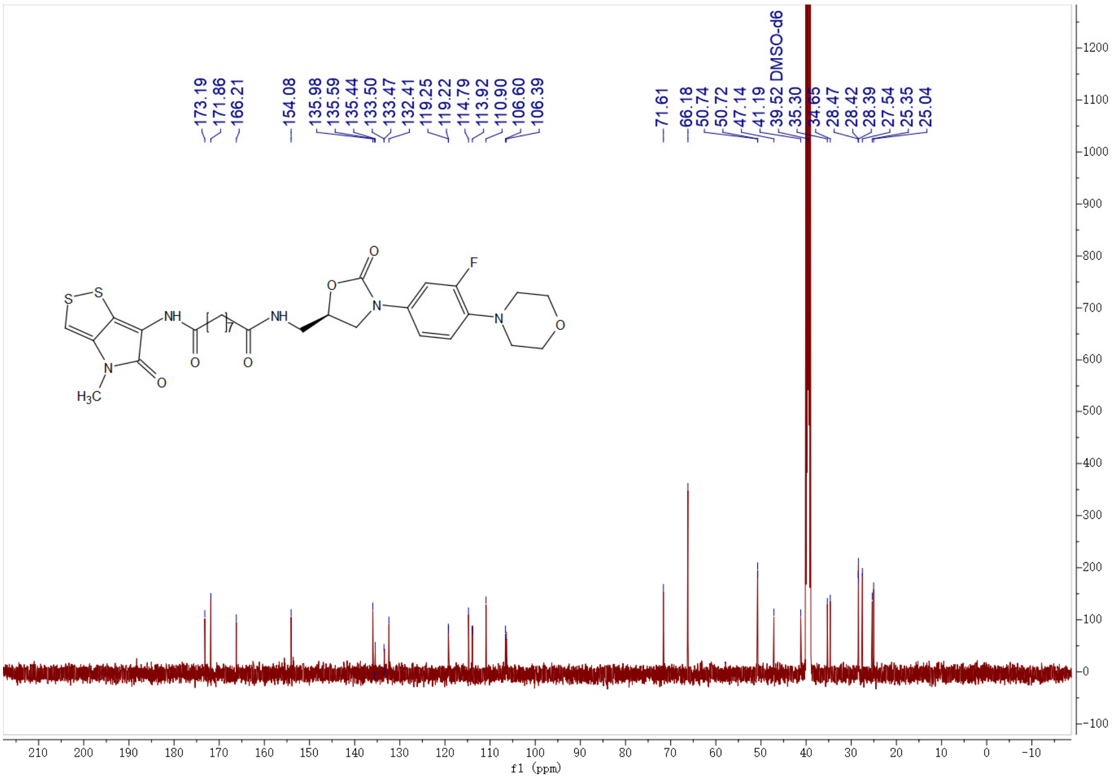

2.8 Compound **T_4-LZD**

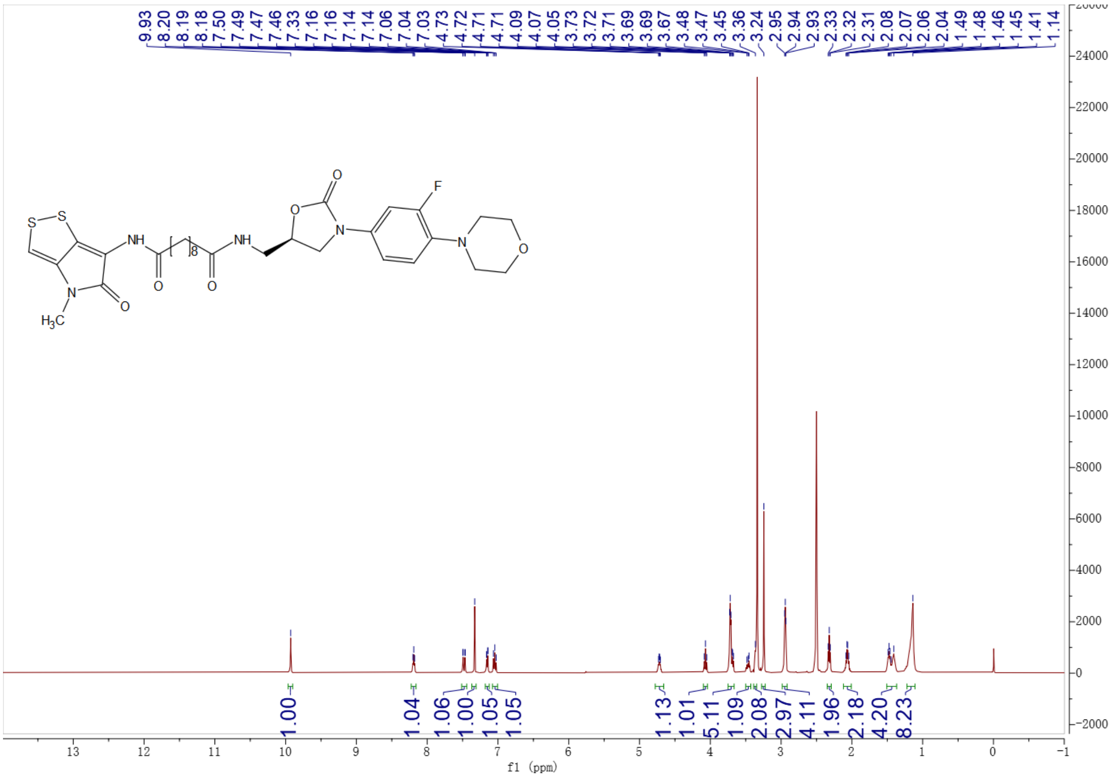

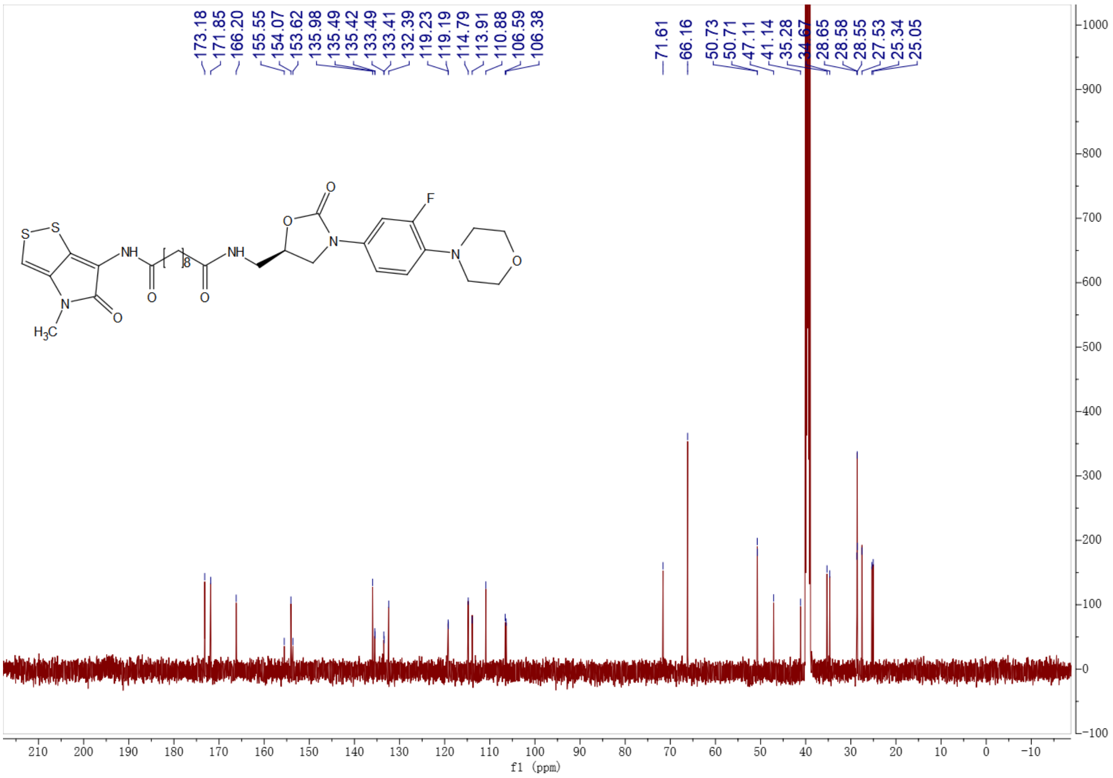

2.9 Compound **T₁-INH**

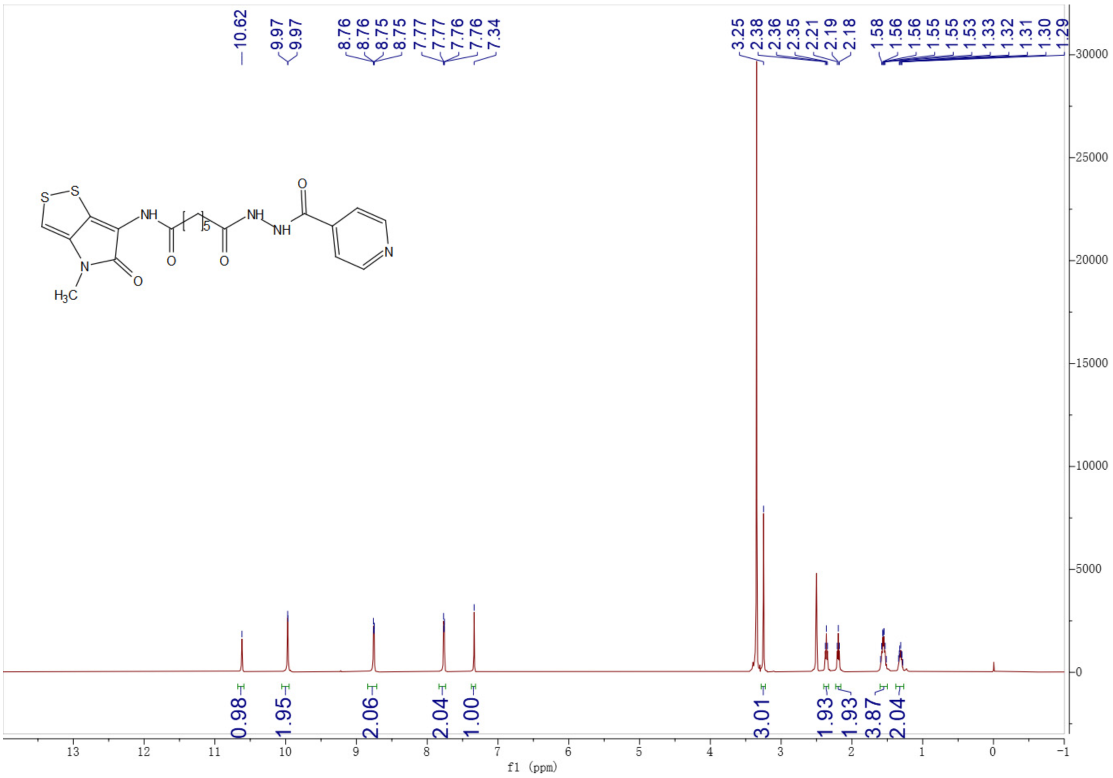

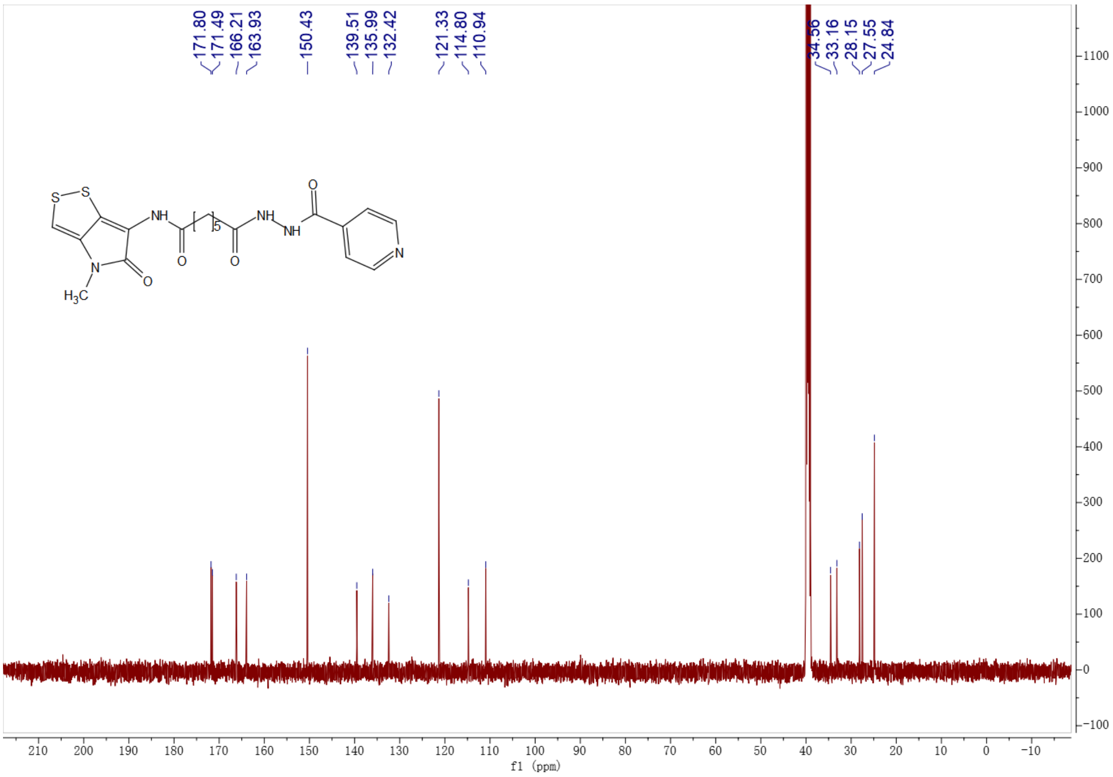

2.10 Compound T_2-INH

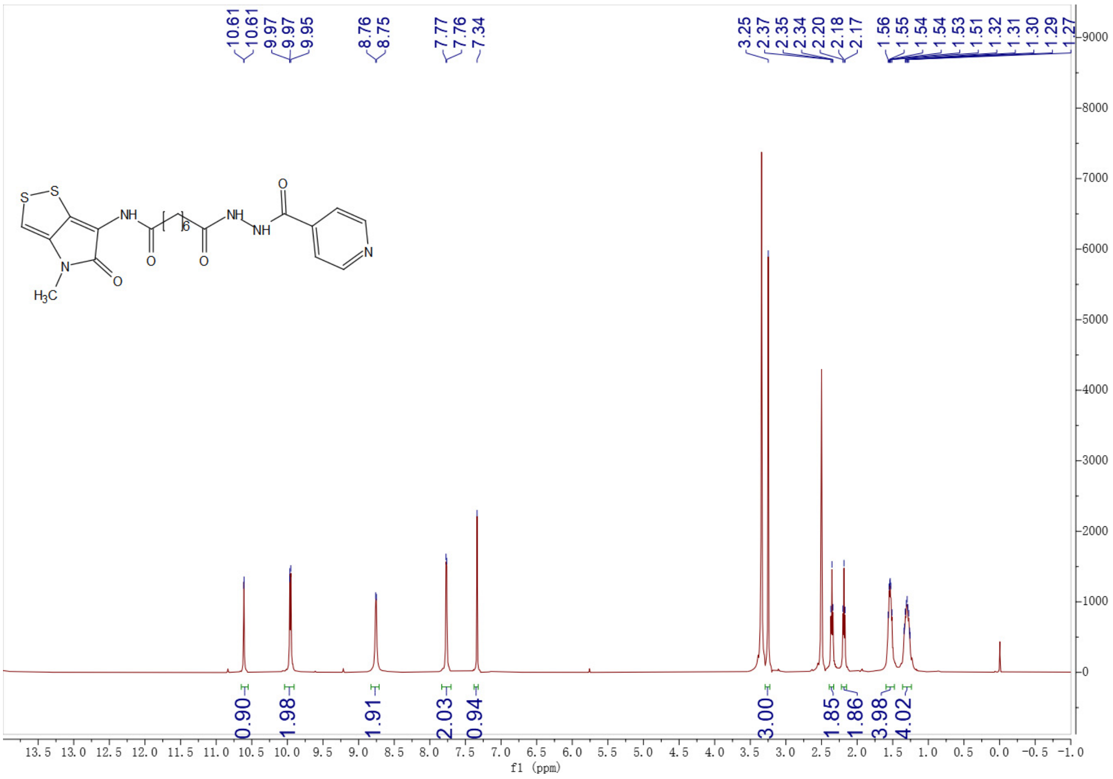

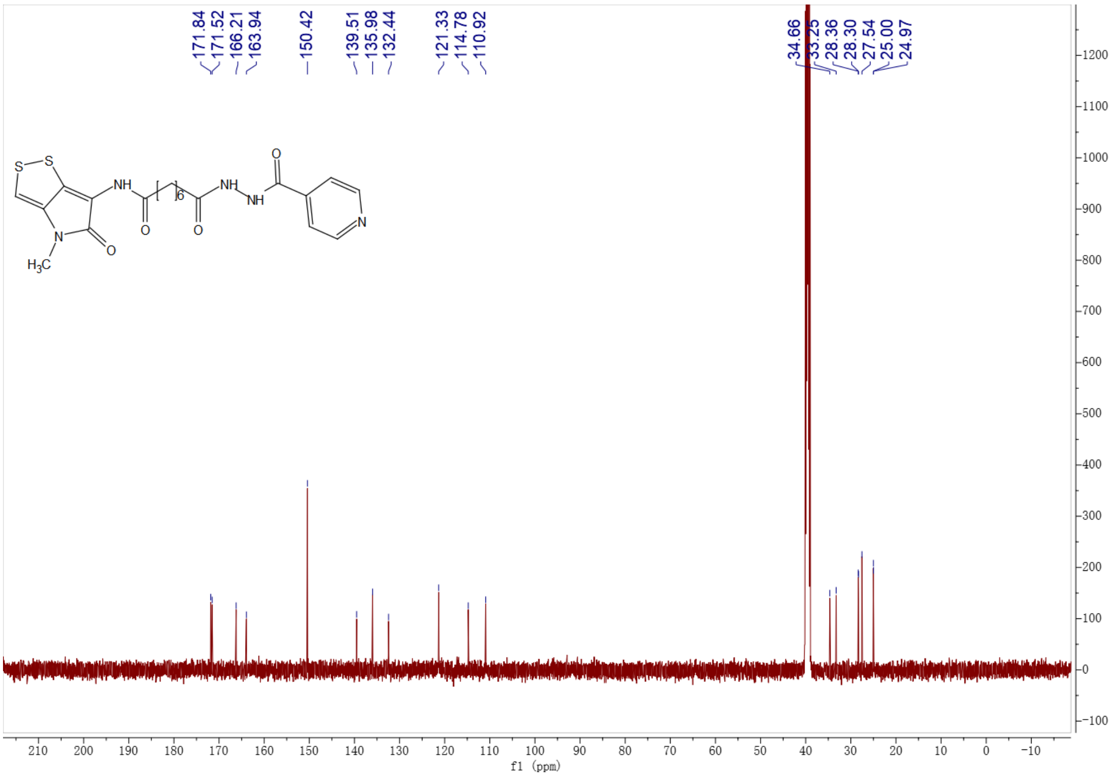

2.11 Compound T_3-INH

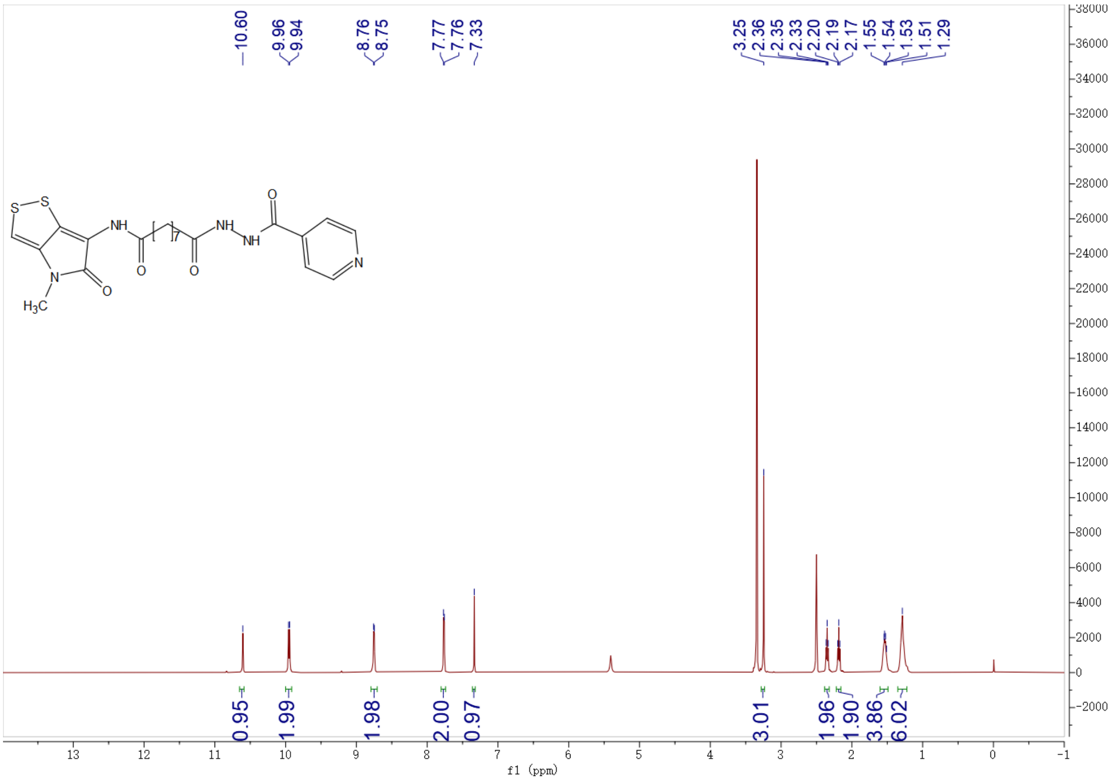

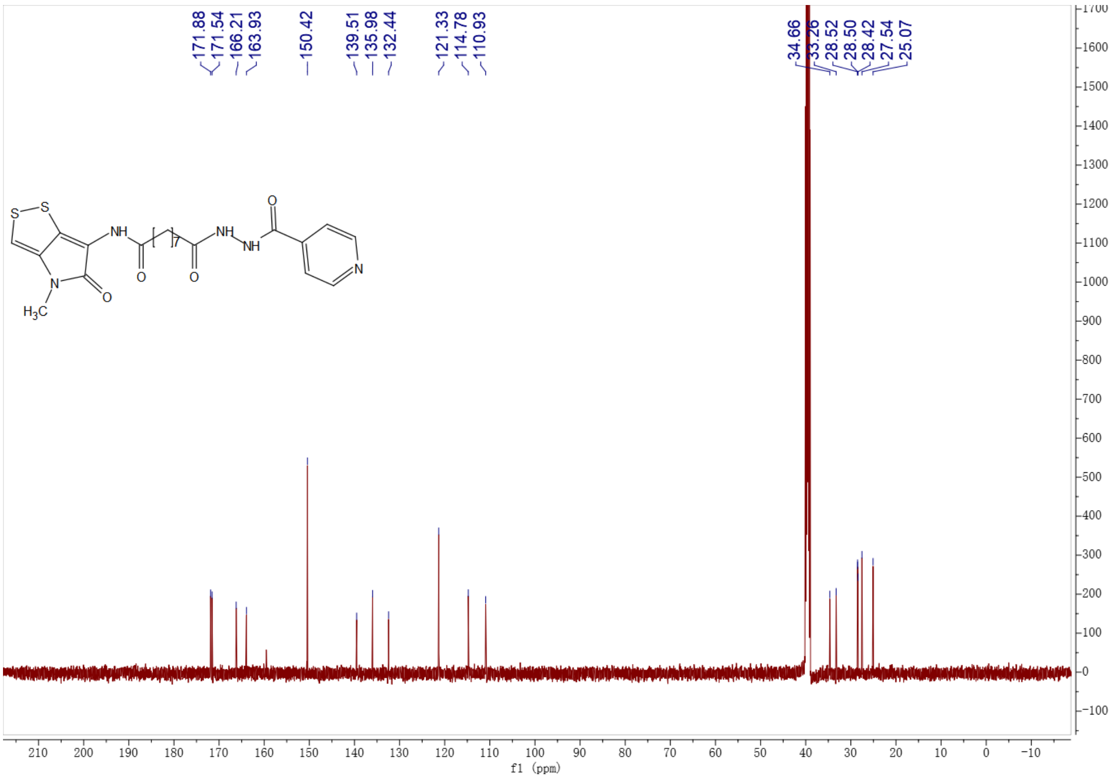

2.12 Compound **T_4-INH**

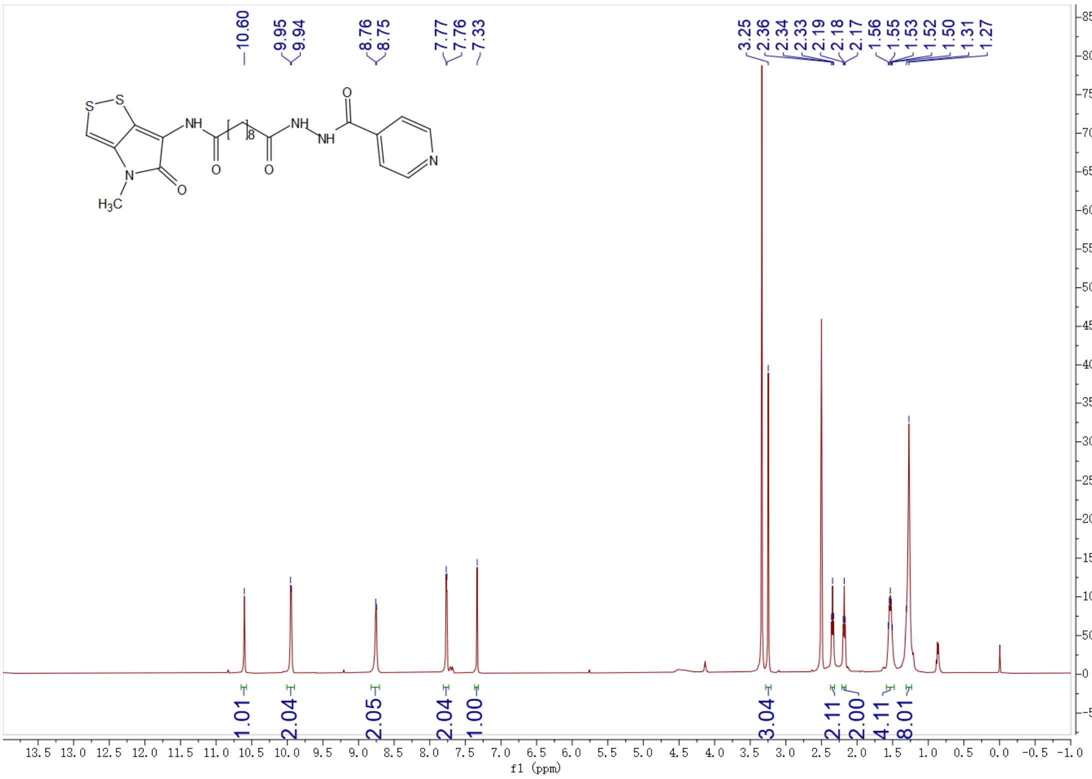

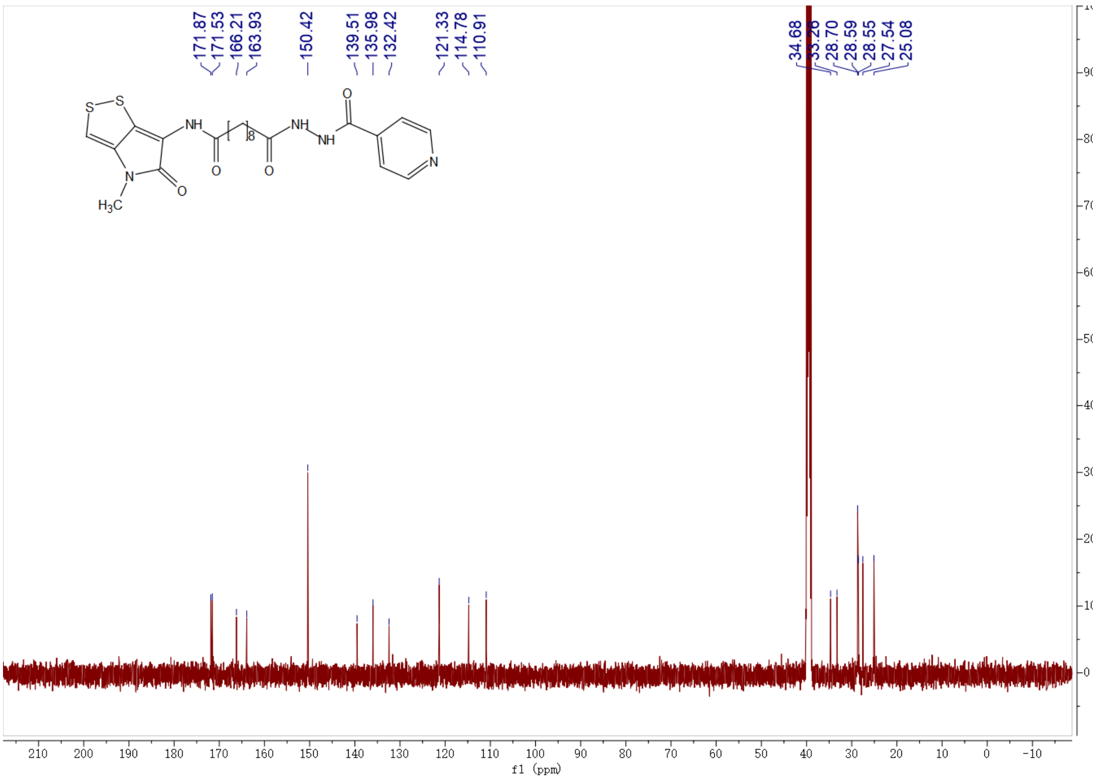

2.13 Compound **T_2-PZA**

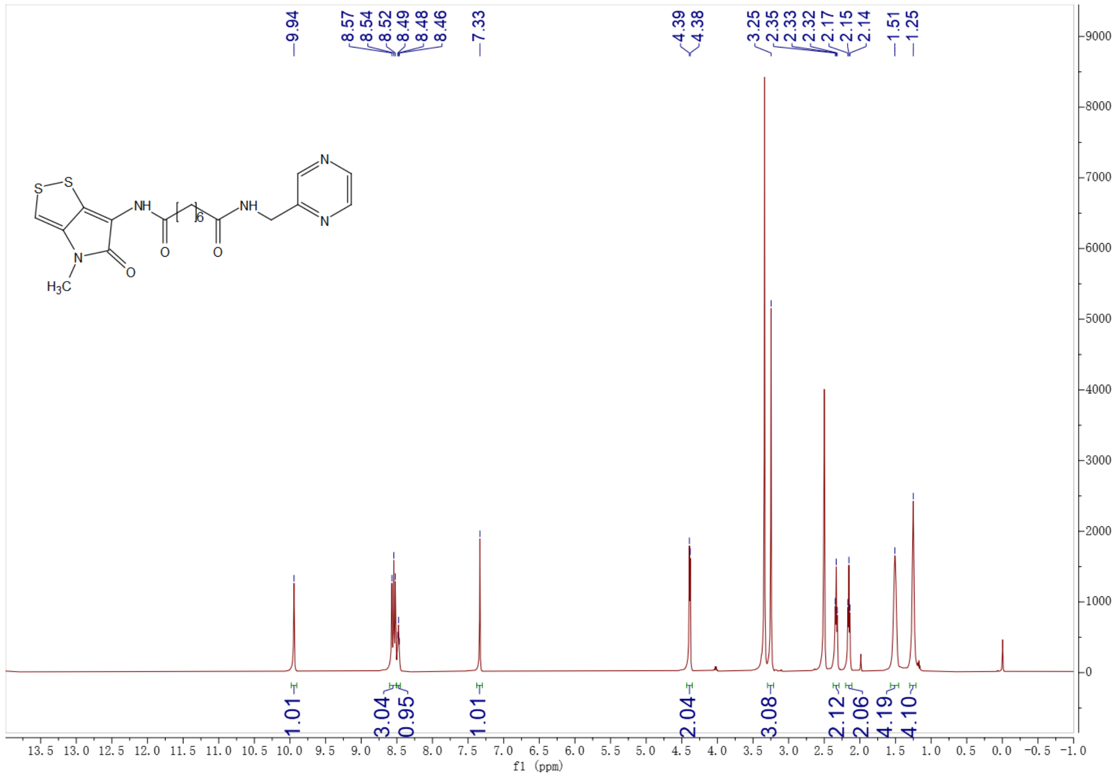

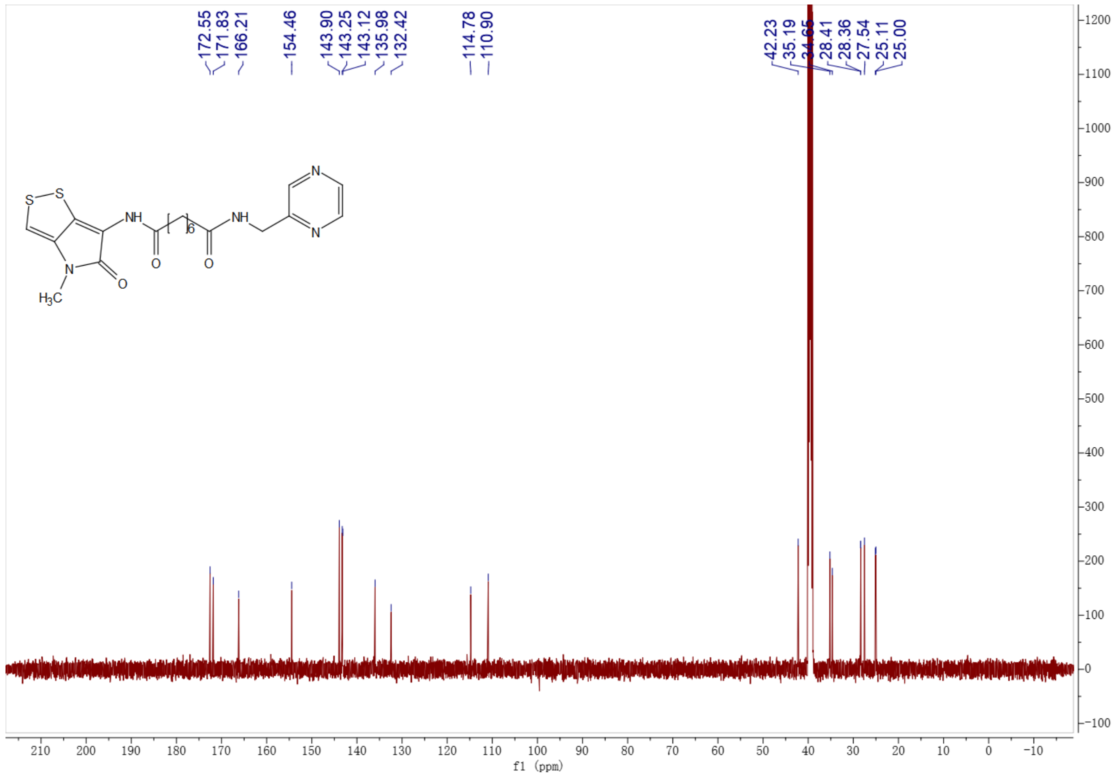

2.14 Compound **T$_3$-PZA**

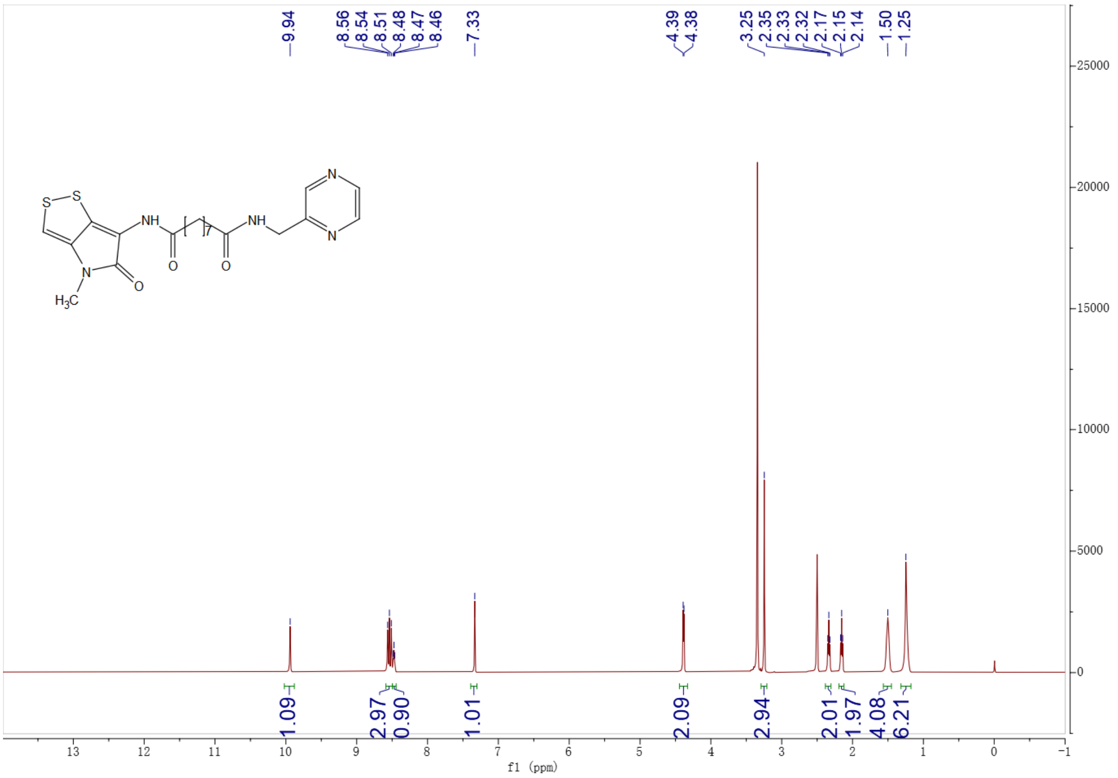

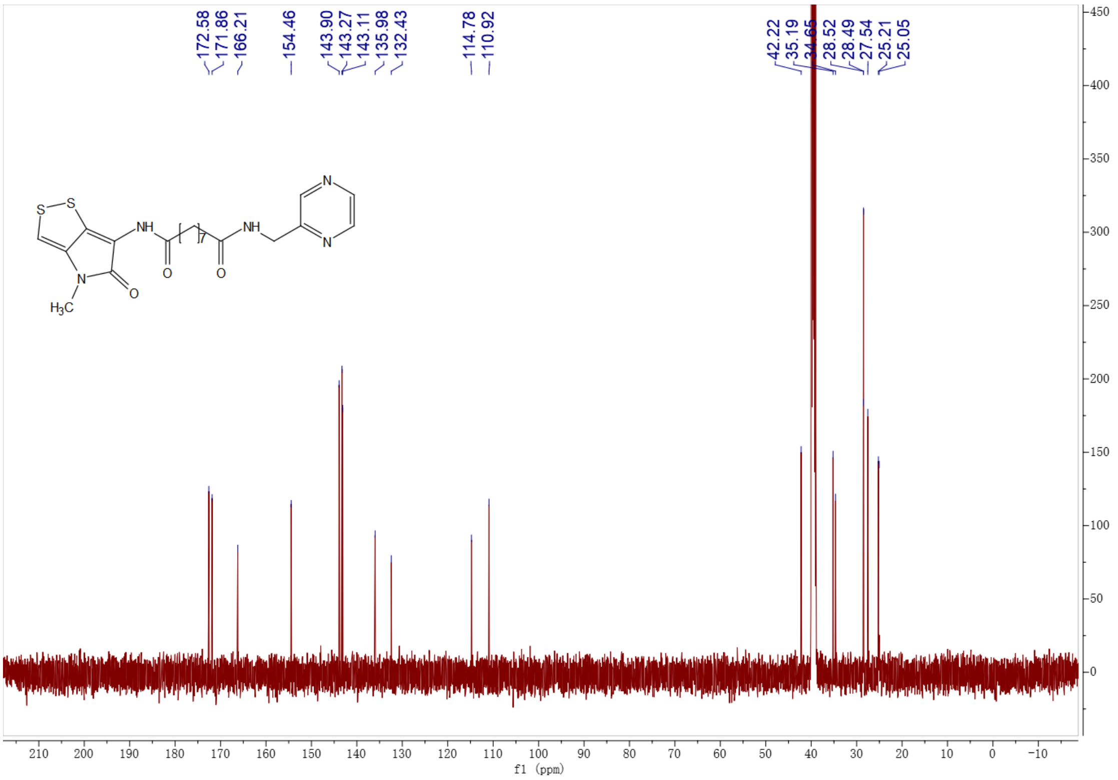

2.15 Compound **T_4-PZA**

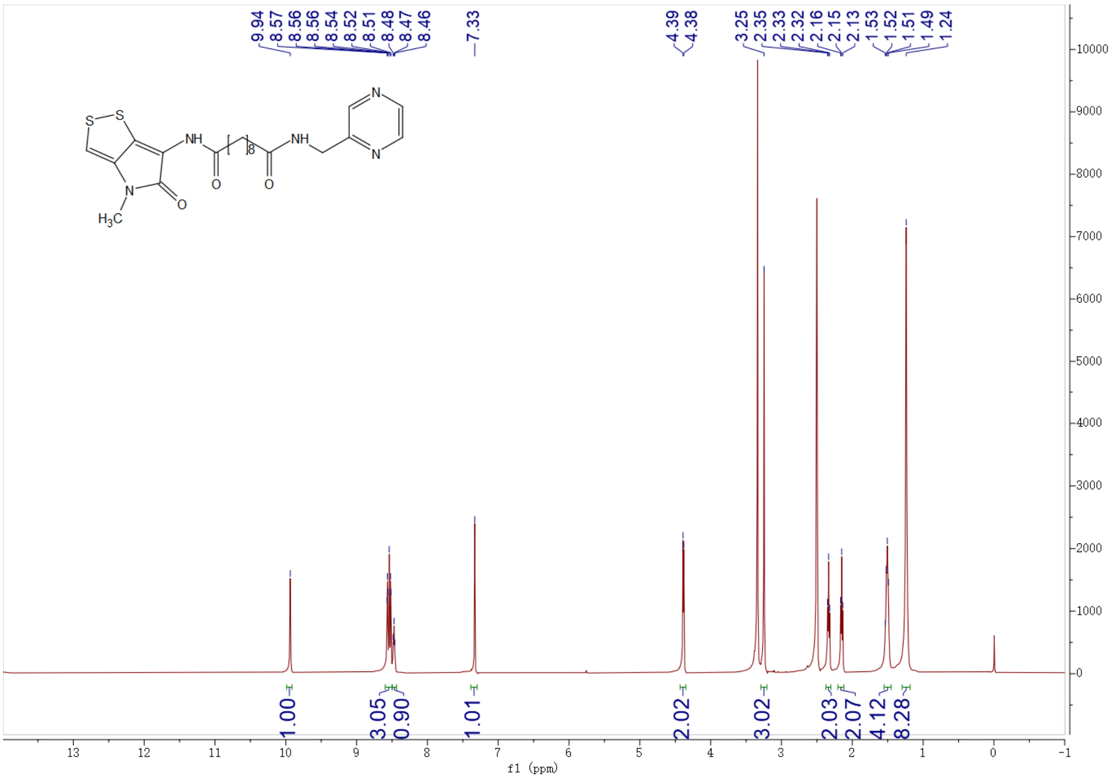
9.94 8.57 8.56 8.54 8.52 8.51 8.48 8.47 8.46
7.33
4.39 4.38
3.25 2.35 2.33 2.32 2.16 2.15 2.13 1.53 1.52 1.51 1.49 1.24
1.00 3.05 0.90 1.01 2.02 3.02 2.03 2.07 4.12 8.28
f1 (ppm)

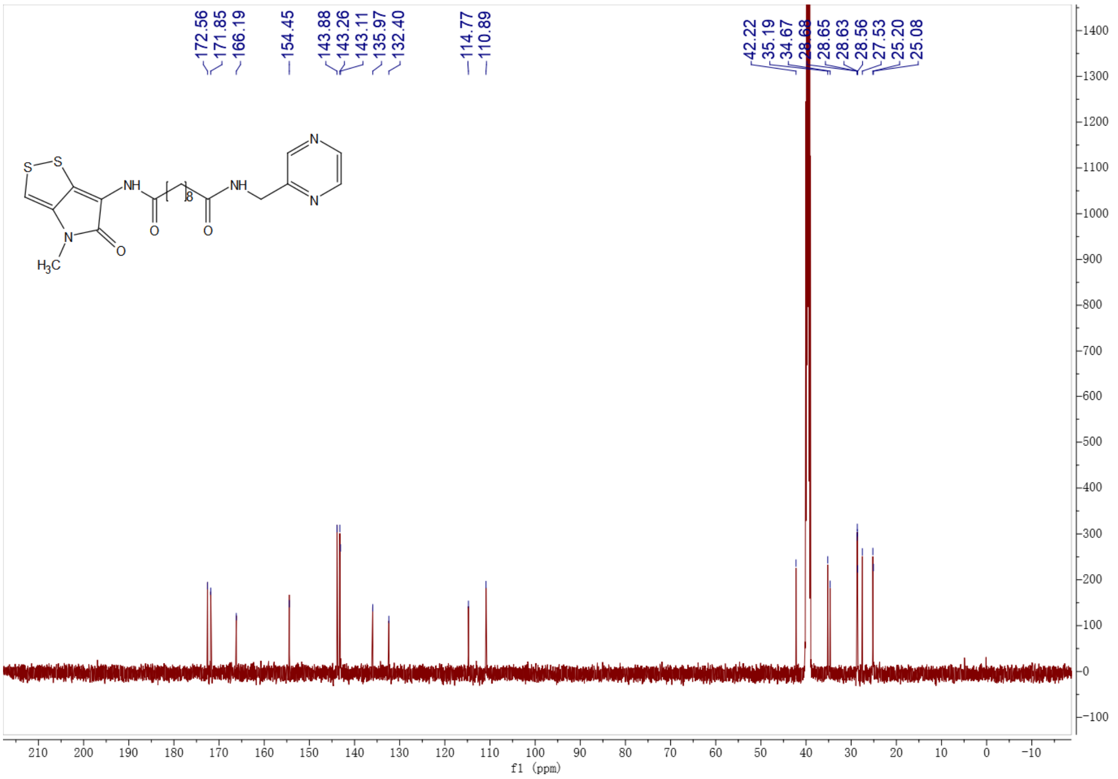
172.56 171.85 166.19
154.45
143.88 143.26 143.11 135.97 132.40
114.77 110.89
42.22 35.19 34.67 28.68 28.65 28.63 28.56 27.53 25.20 25.08
f1 (ppm)

3. ESI mass spectra and HPLC chromatograms of all compounds

3.1 Compound $\mathbf{T_1}$**-CS**

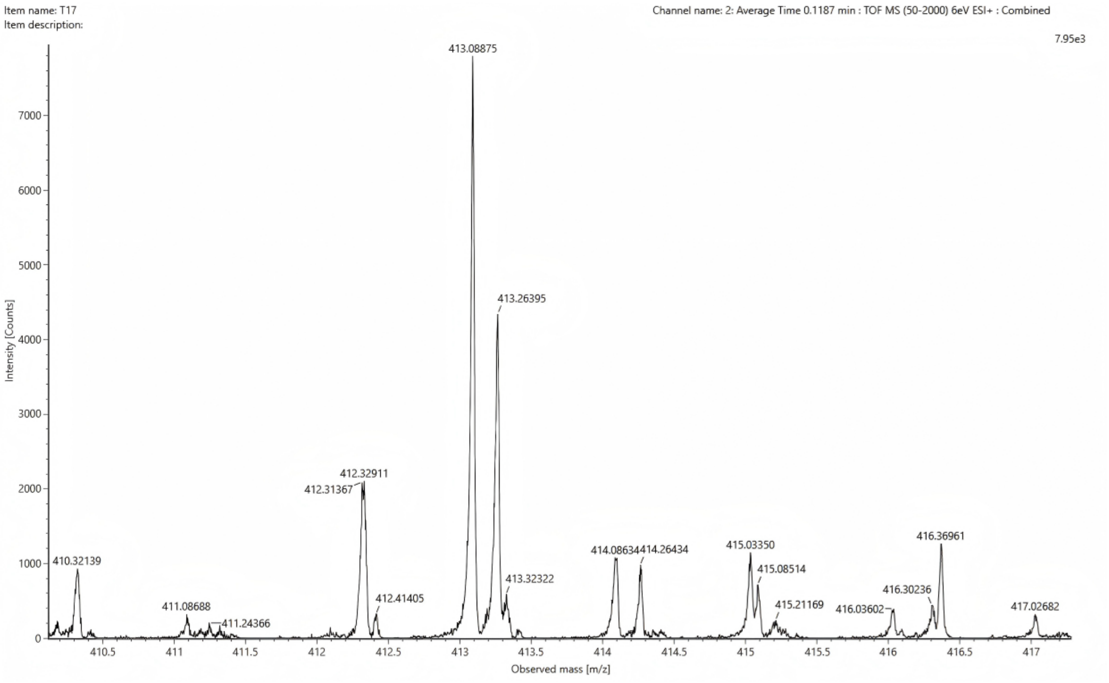

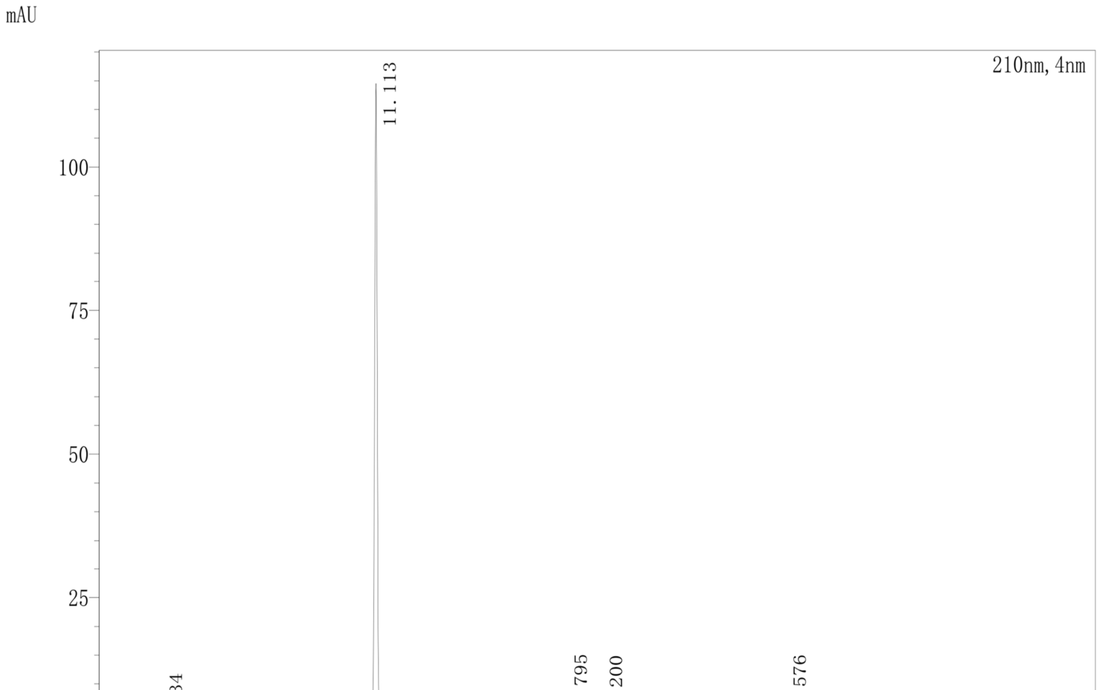

3.2 Compound $\mathbf{T_2}$**-CS**

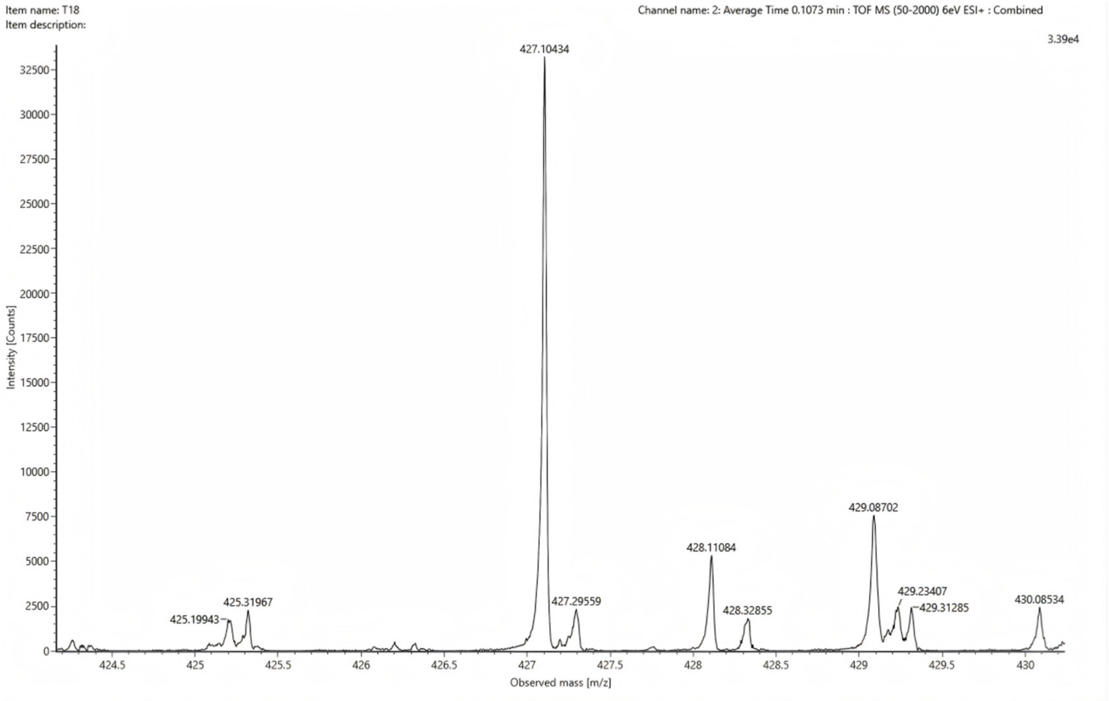

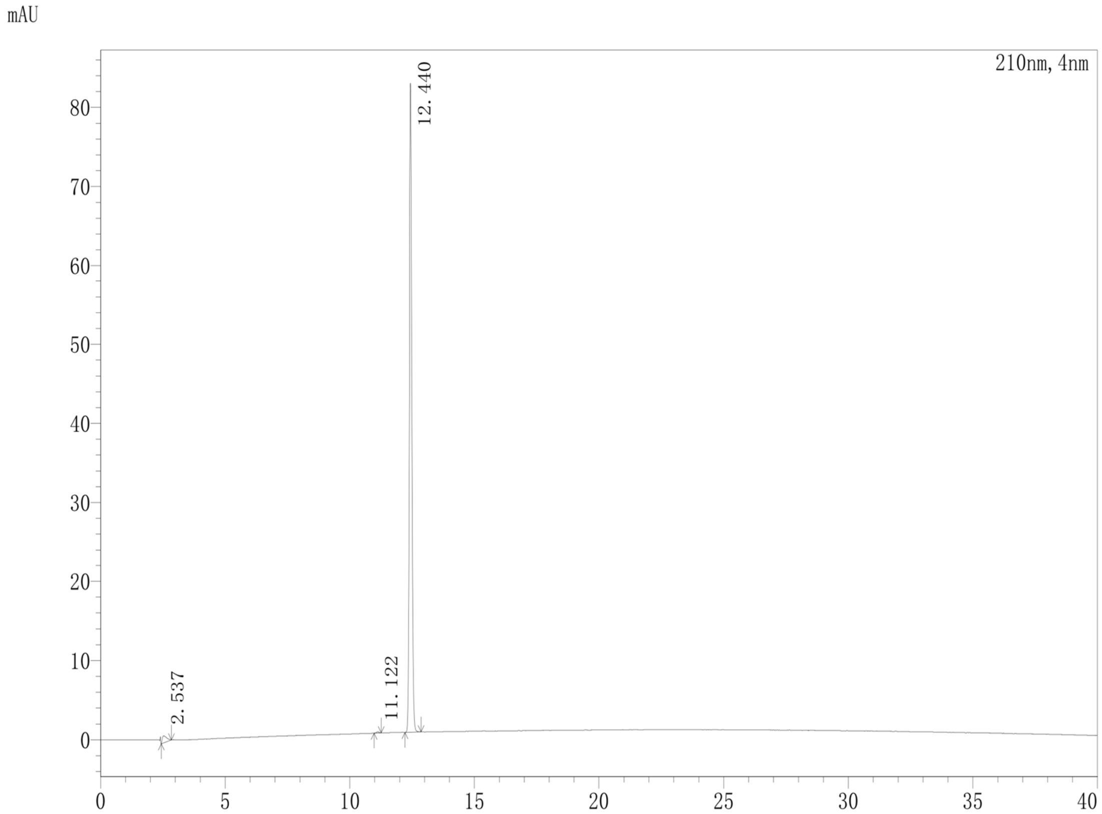

3.3 Compound **T_3-CS**

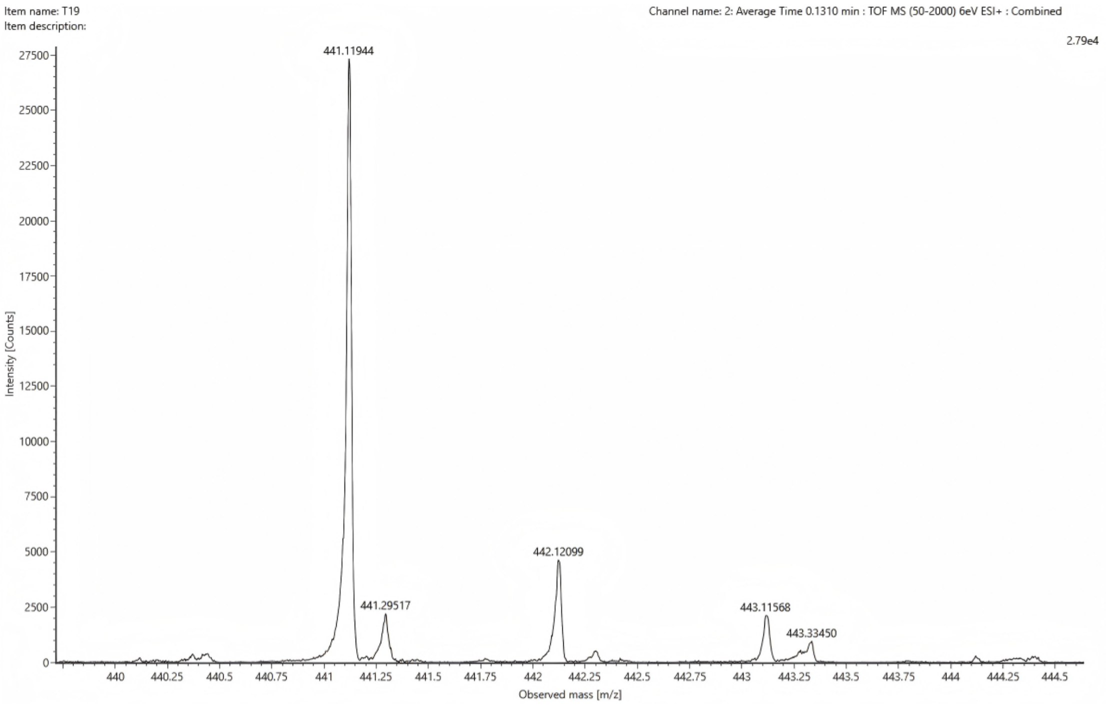

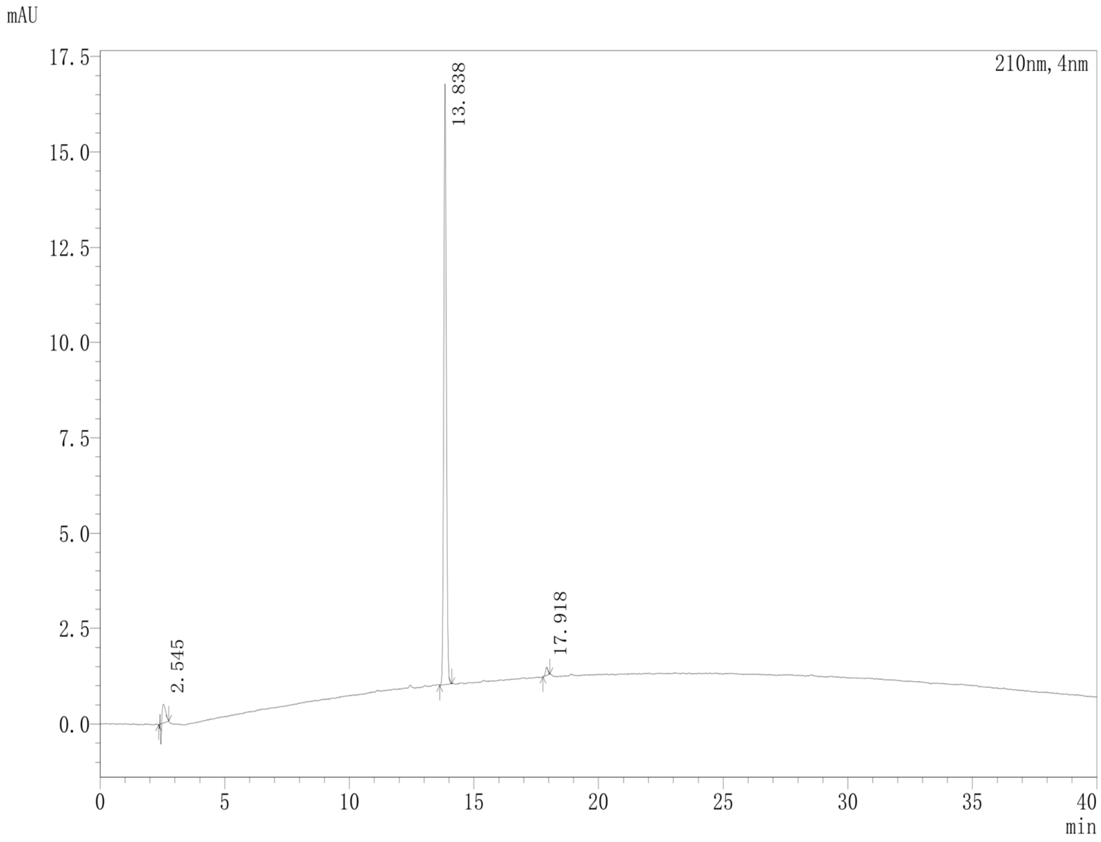

3.4 Compound $\mathbf{T_4}$-**CS**

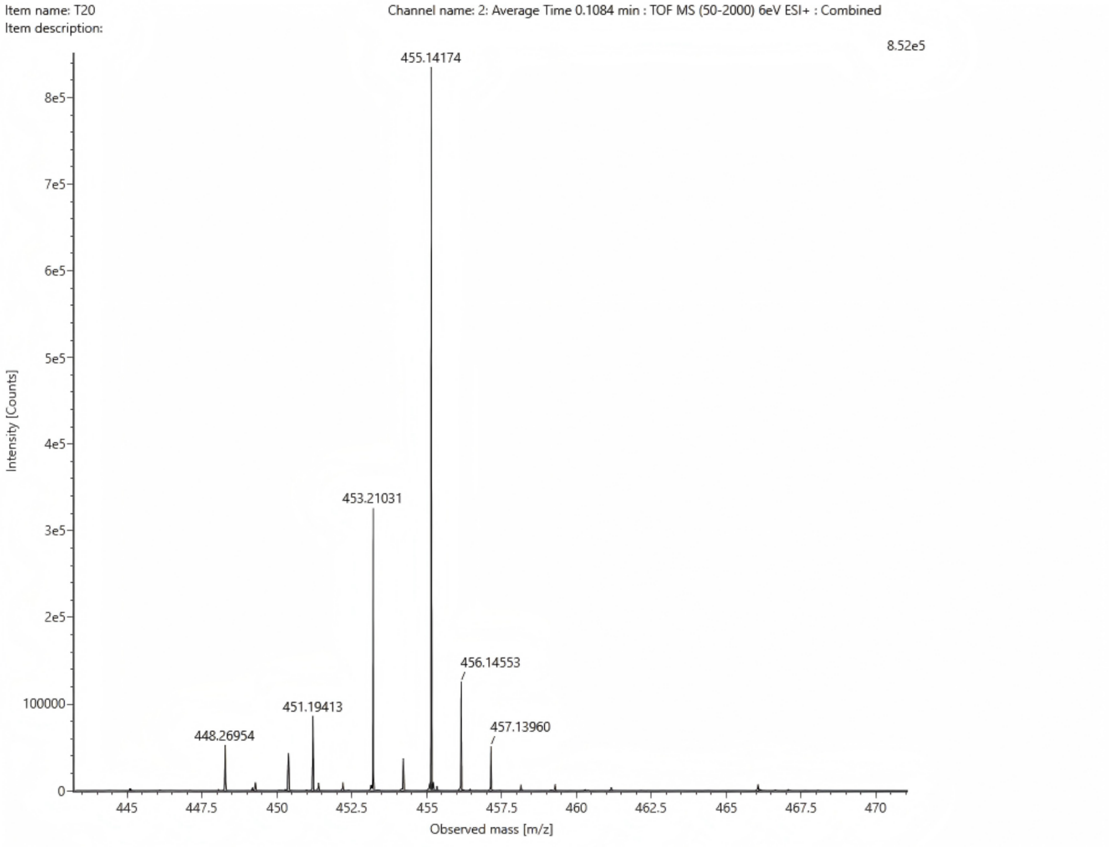

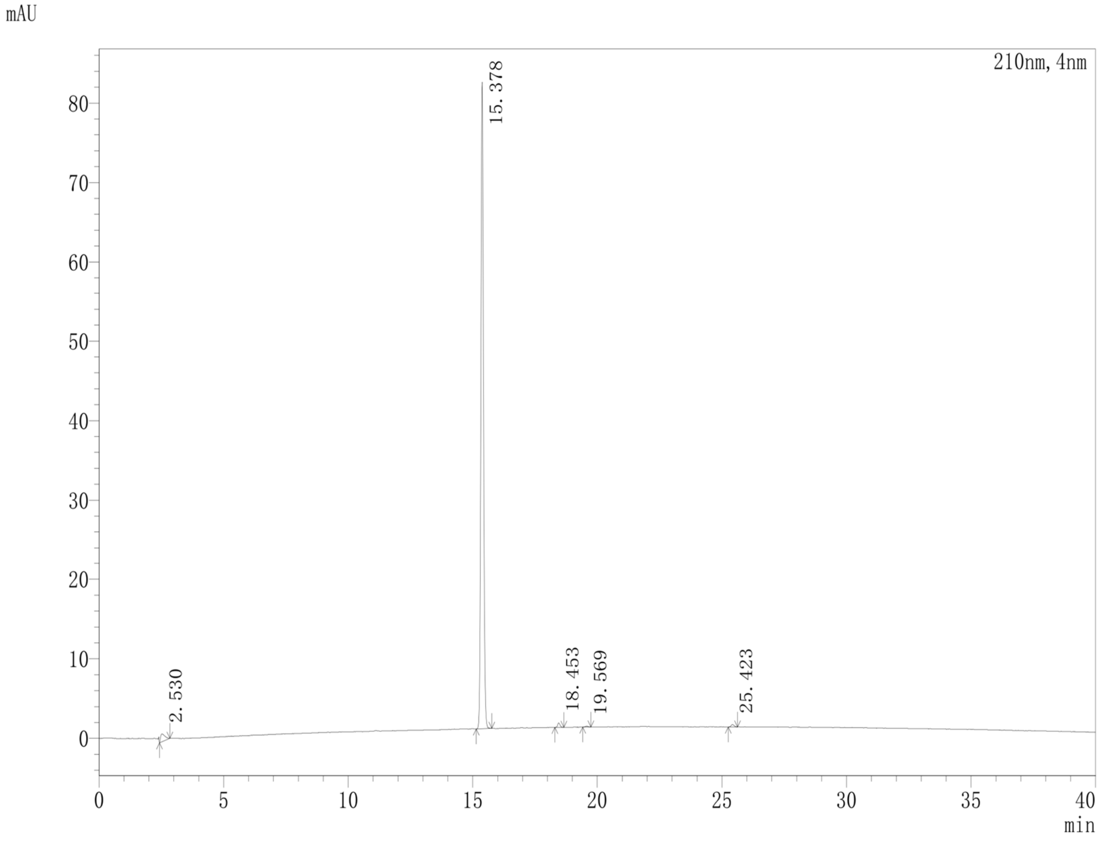

3.5 Compound **T_1-LZD**

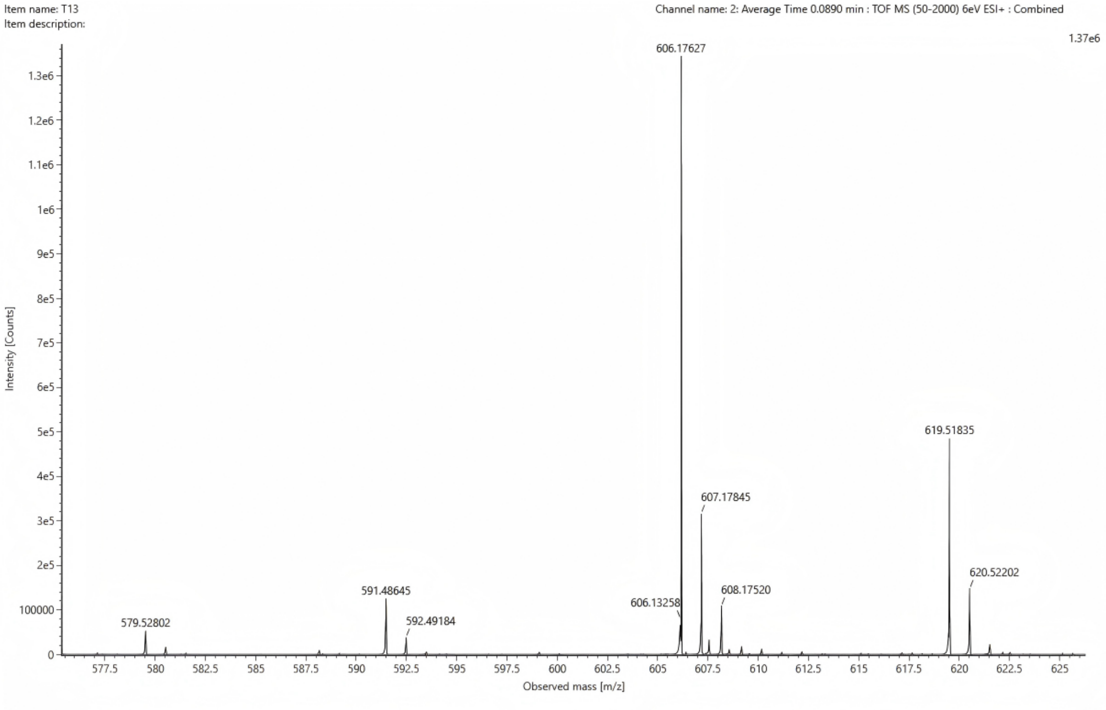

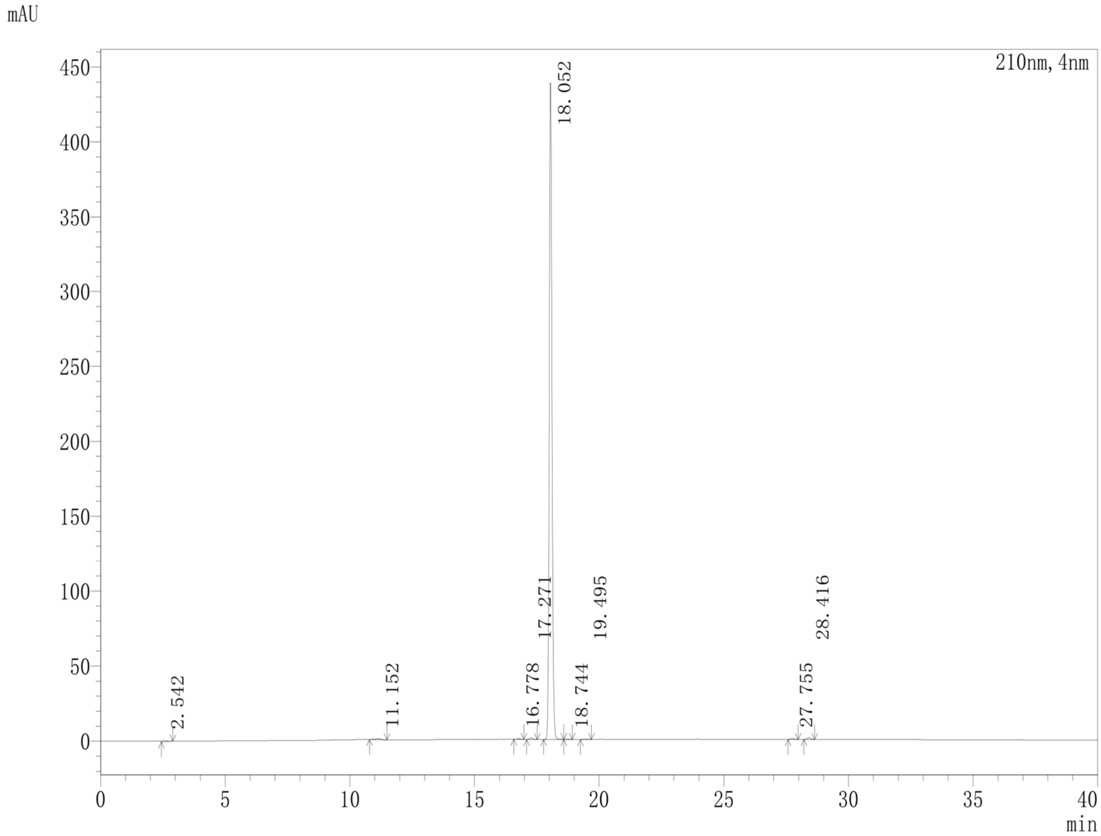

3.6 Compound **T_2-LZD**

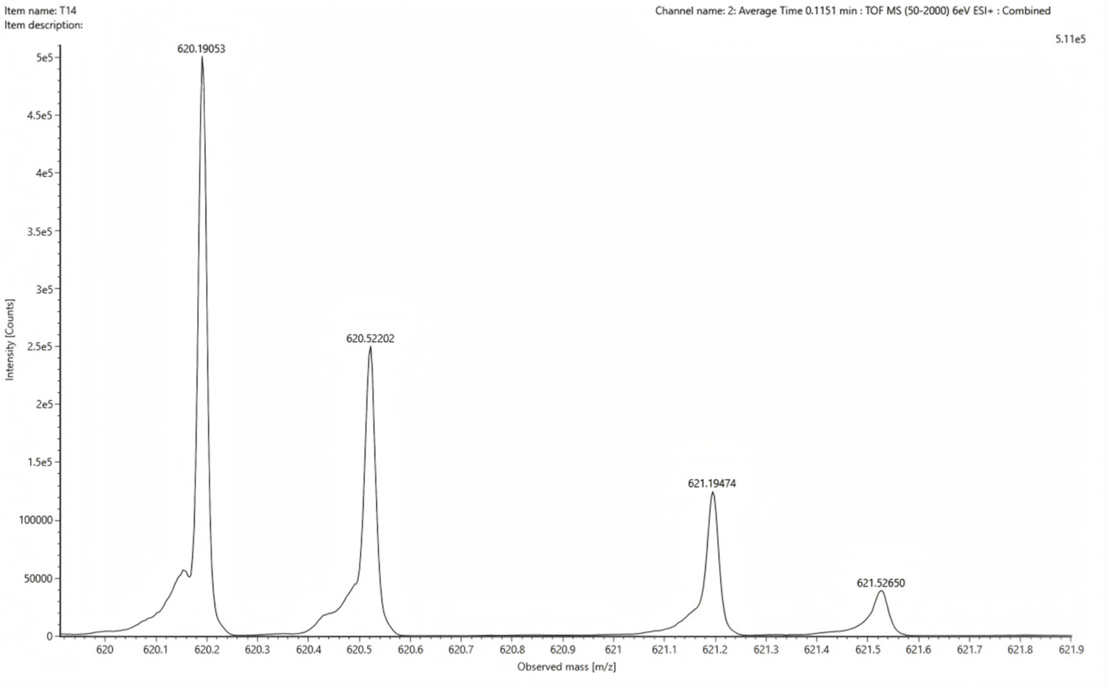

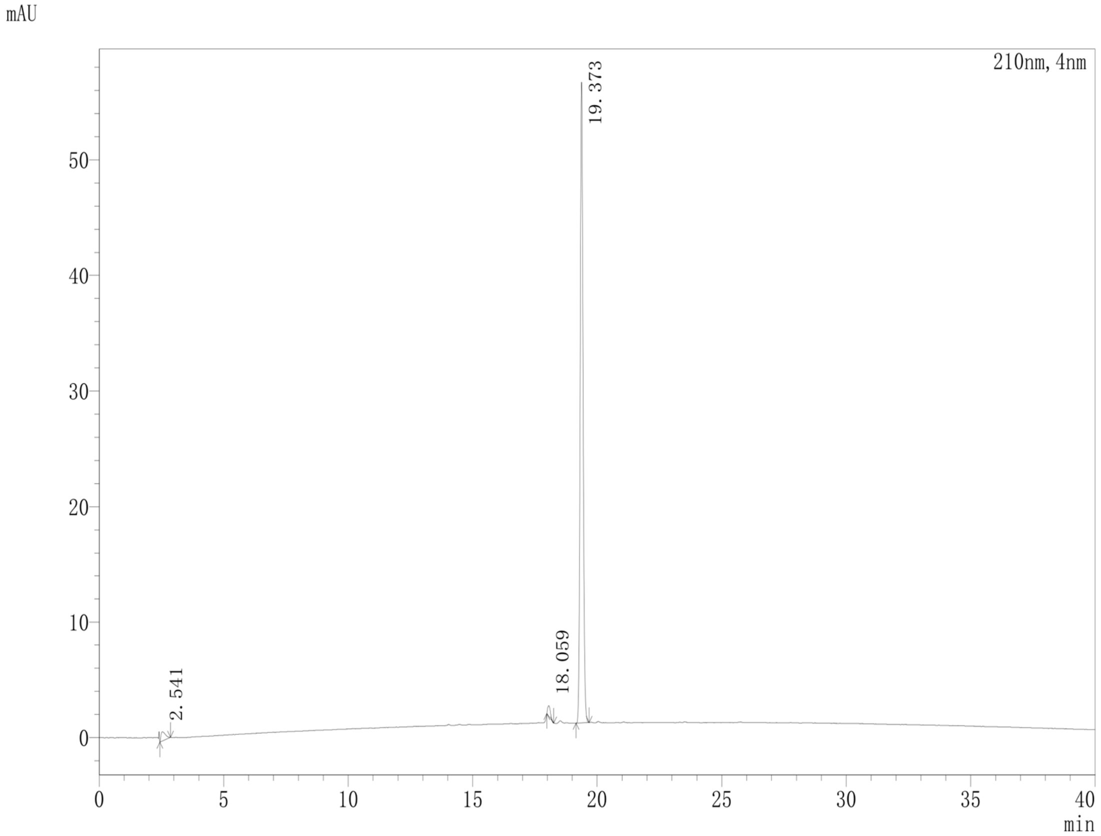

3.7 Compound **T_3-LZD**

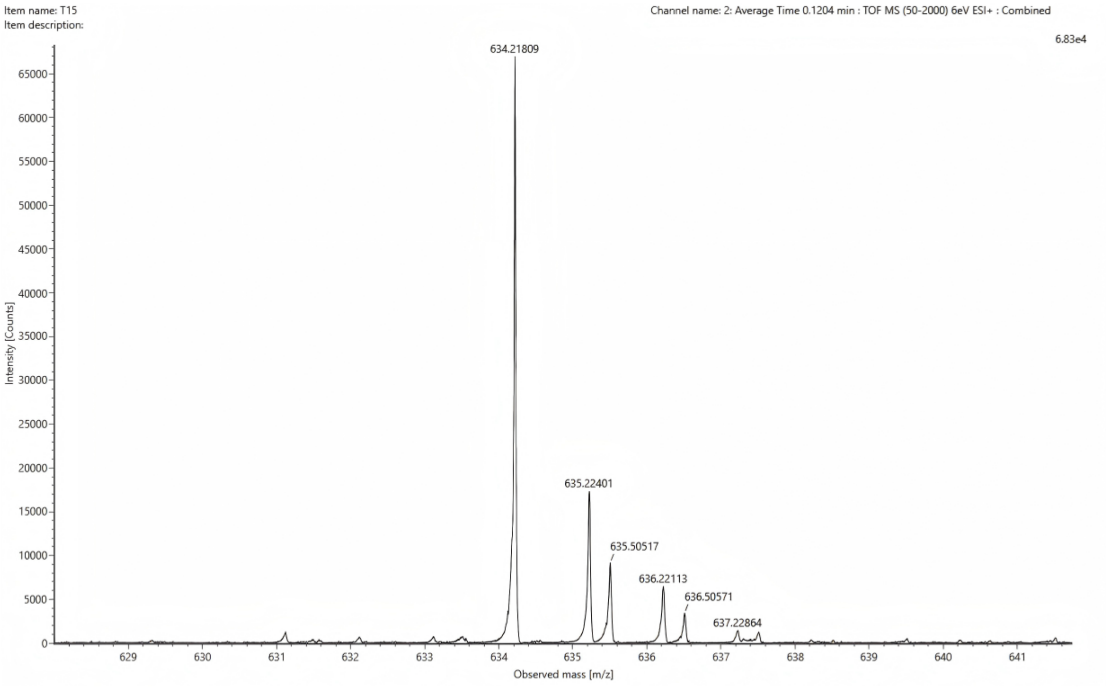

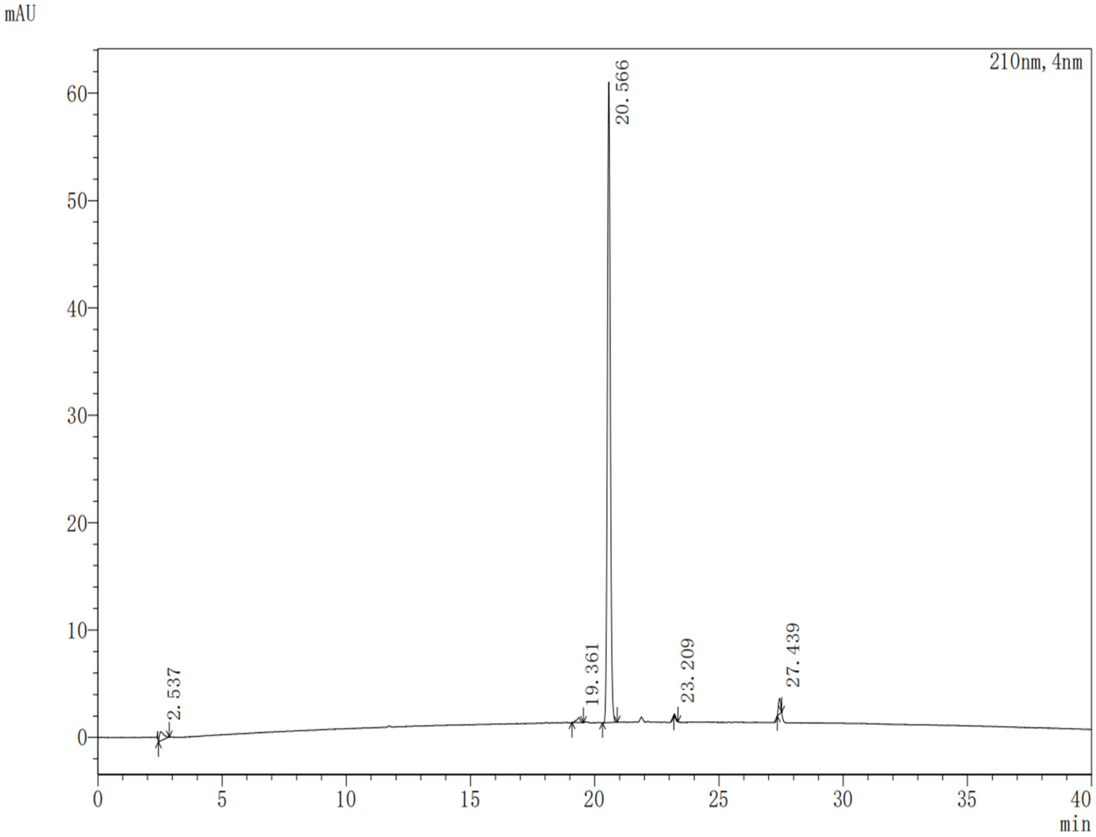

3.8 Compound **T_4-LZD**

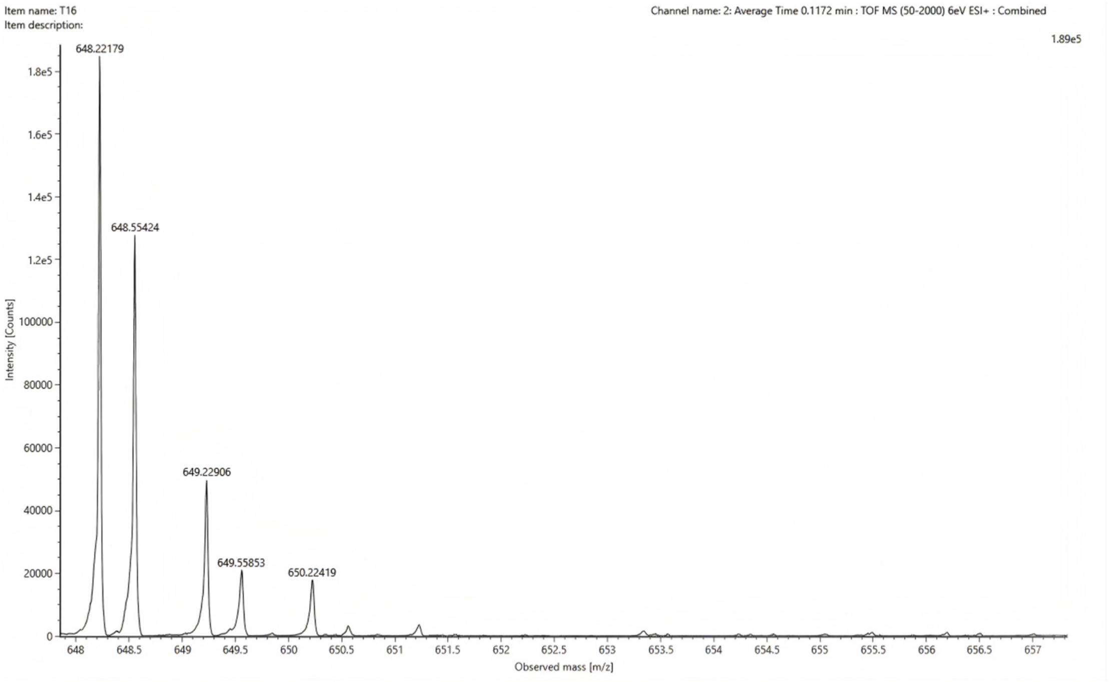

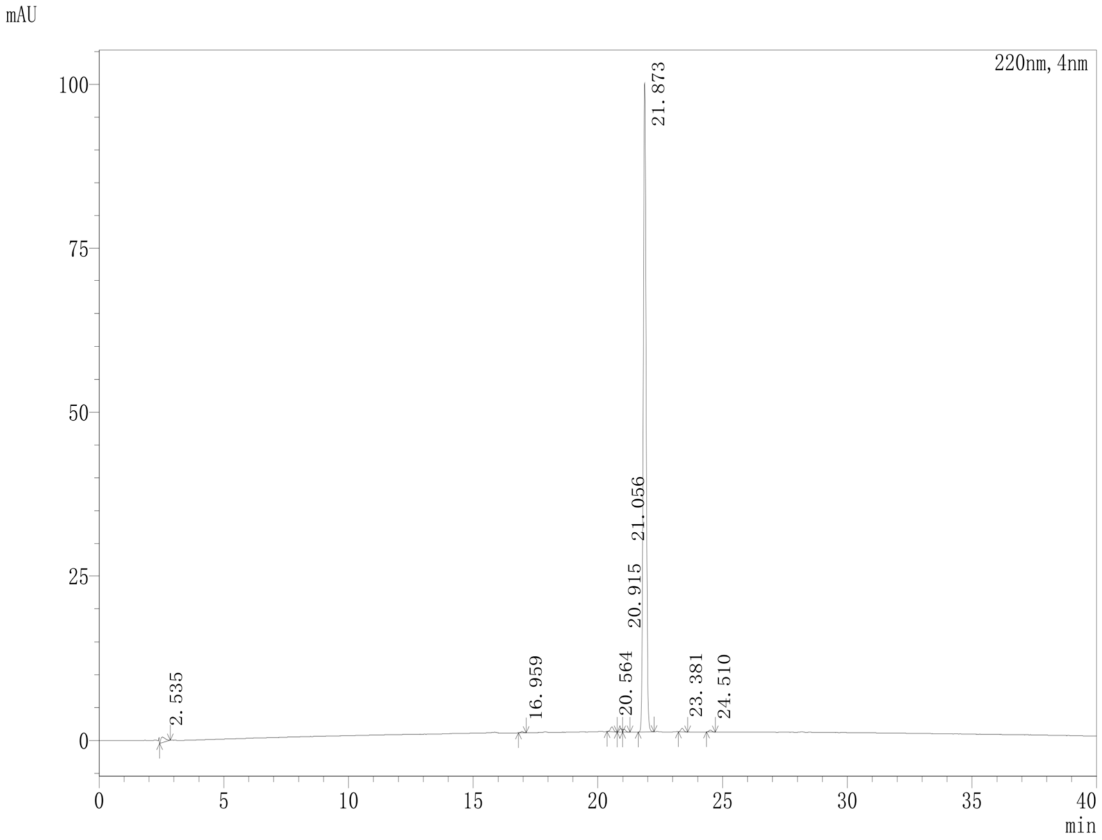

3.9 Compound $\mathbf{T_1}$**-INH**

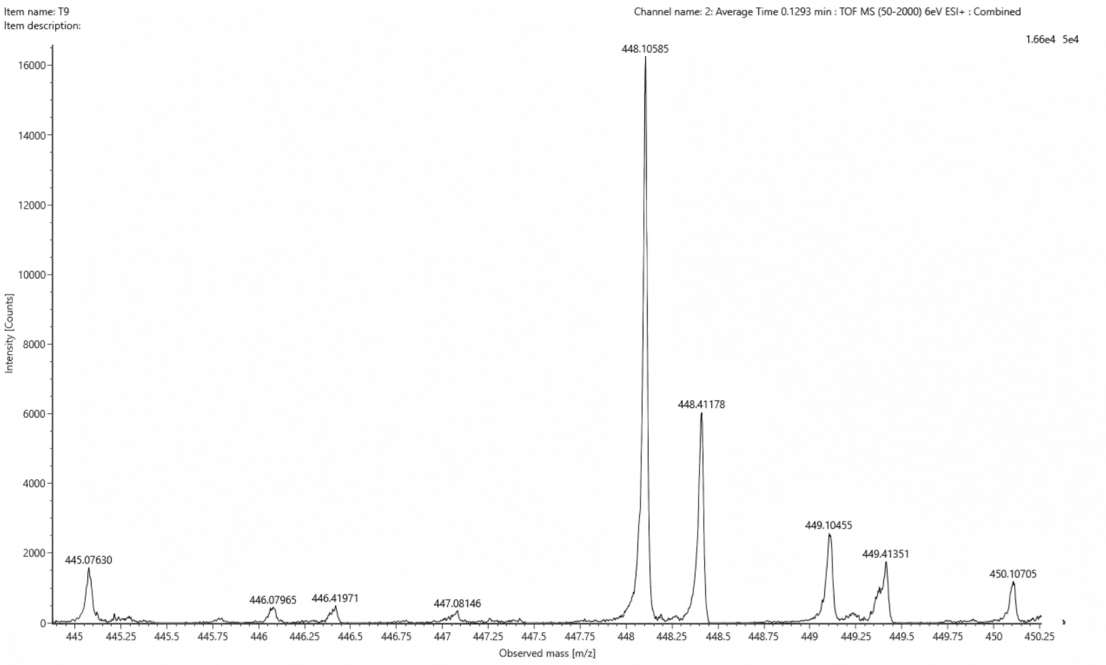

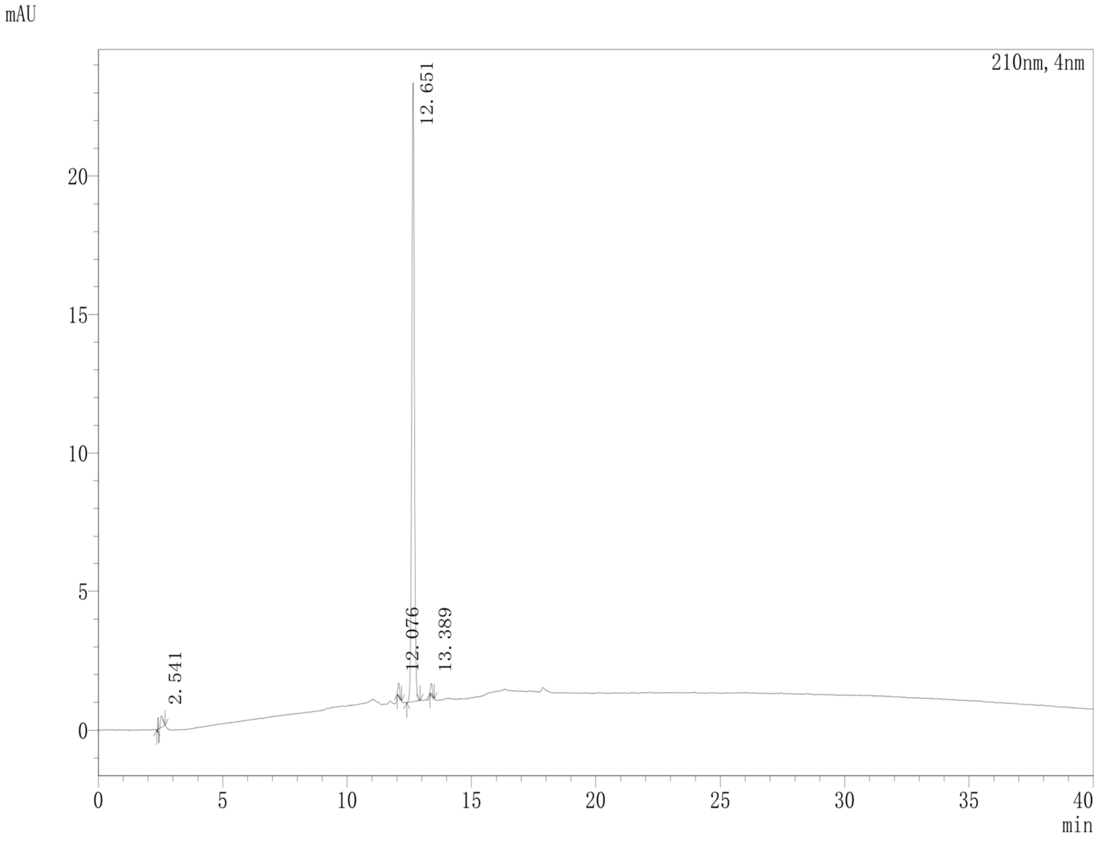

3.10 Compound **T_2-INH**

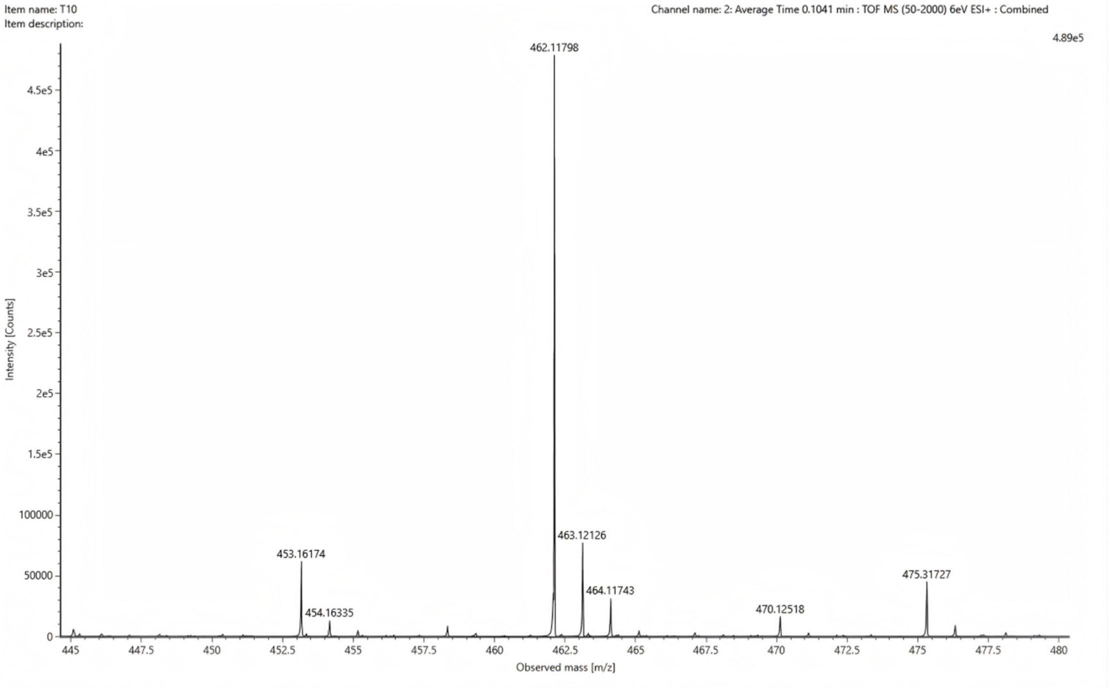

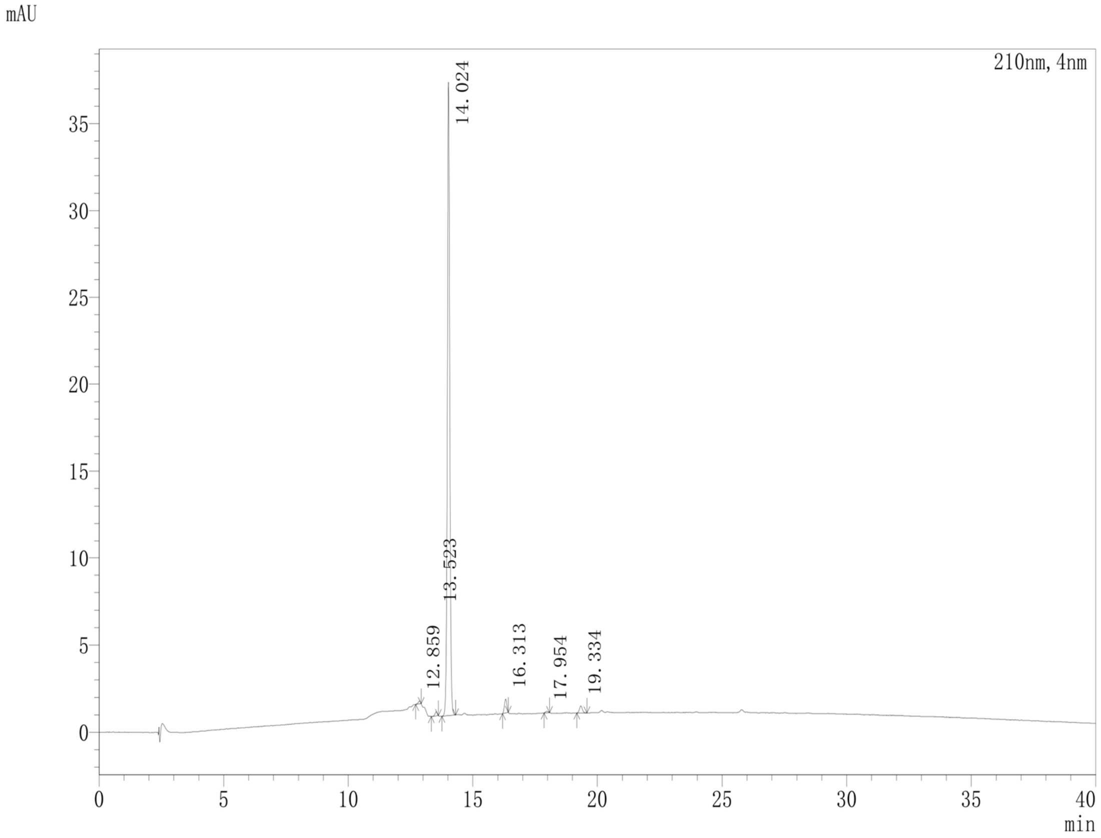

3.11 Compound **T_3-INH**

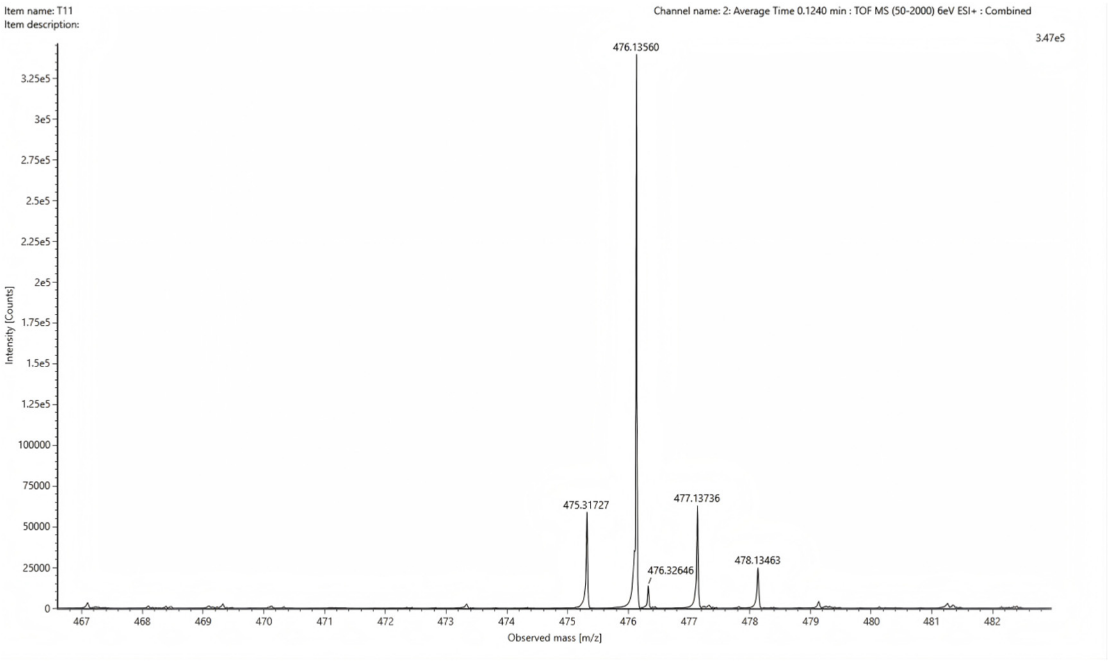

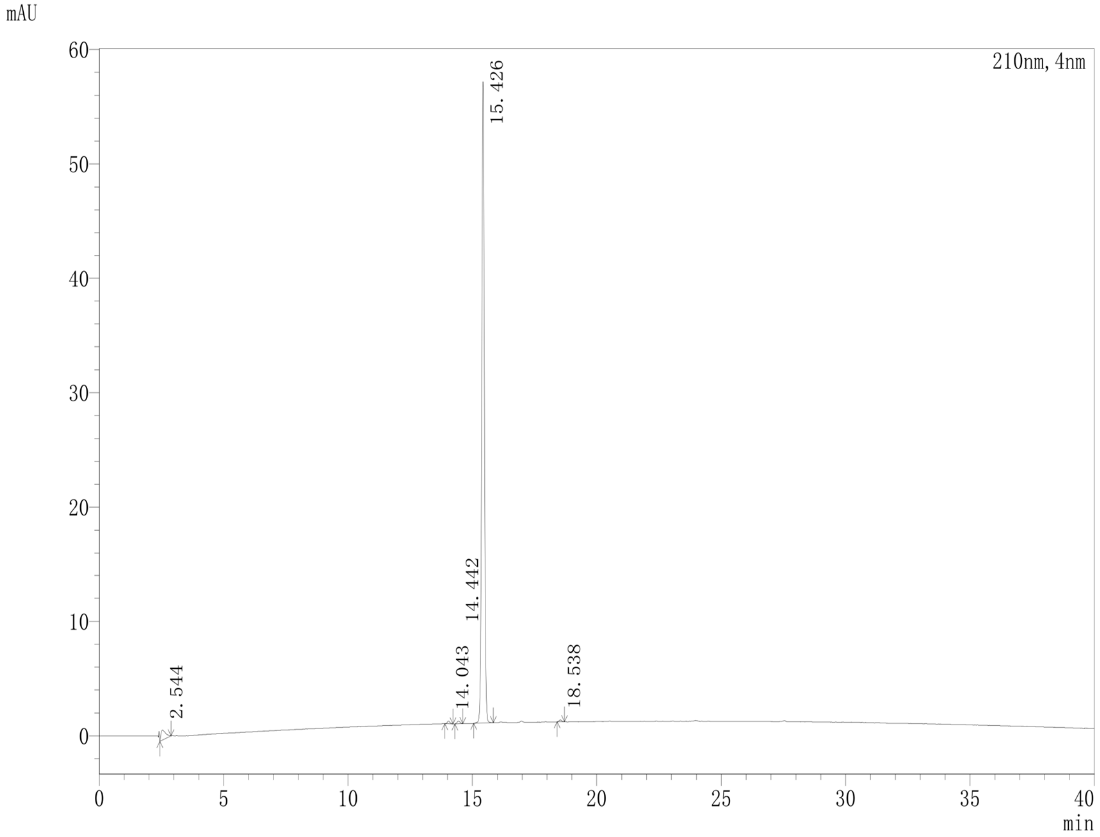

3.12 Compound **T_4-INH**

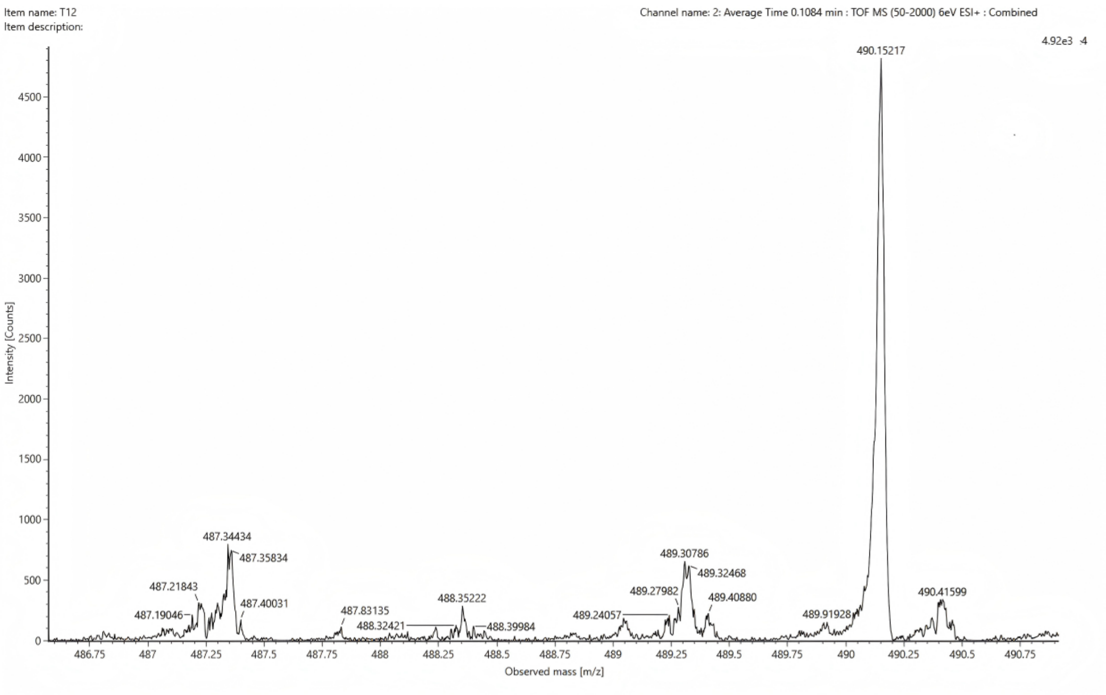

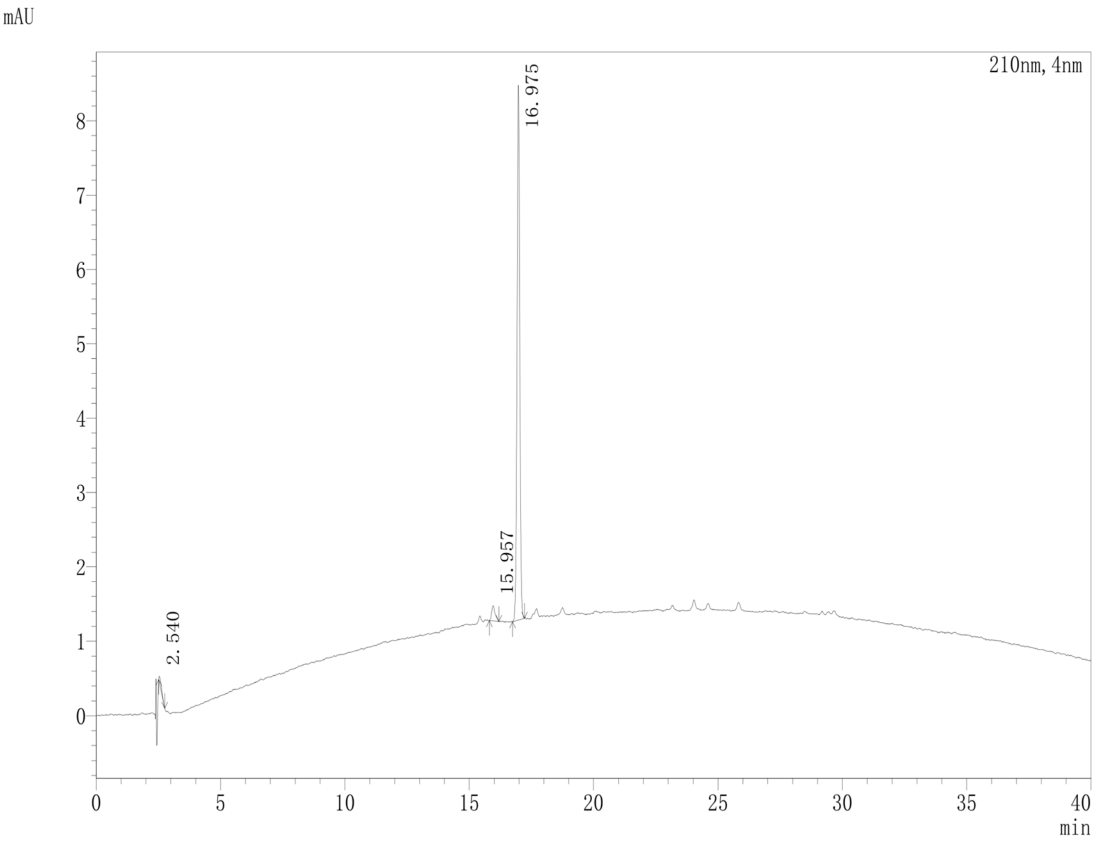

3.13 Compound **T_2-PZA**

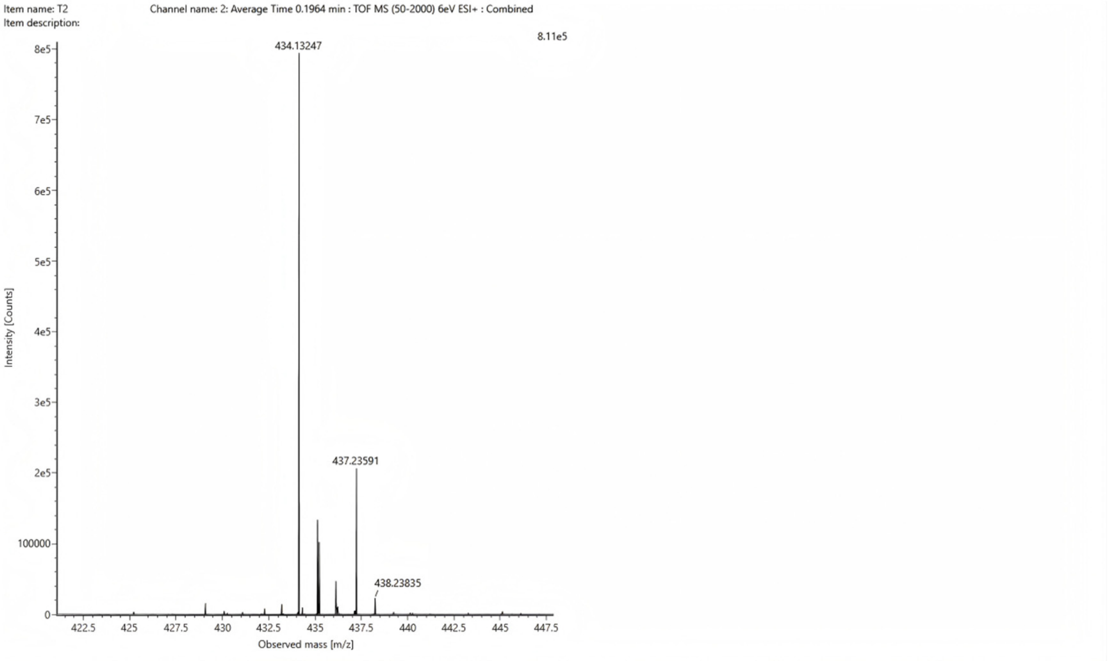

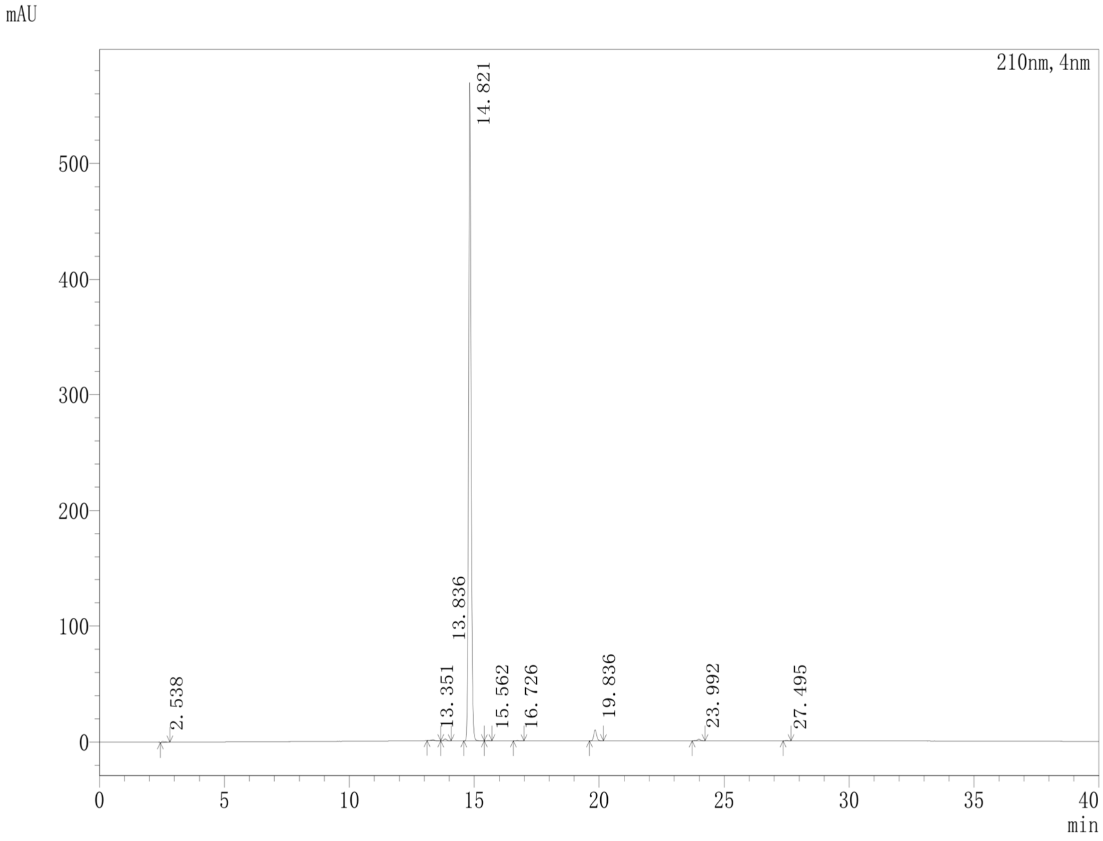

3.14 Compound **T_3-PZA**

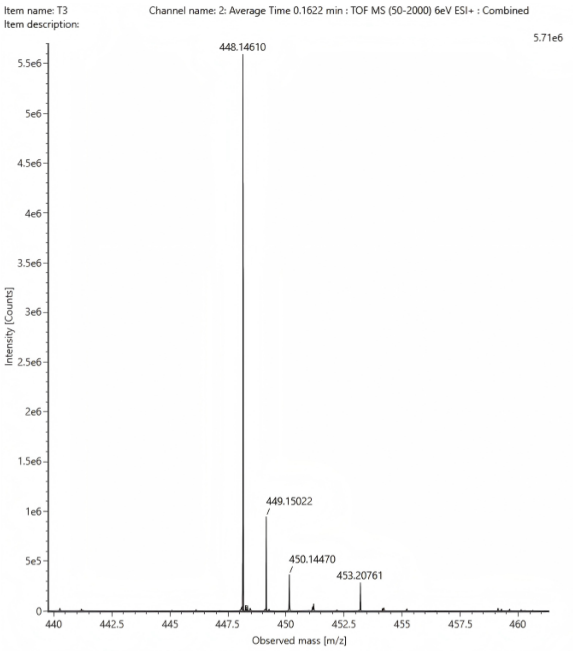

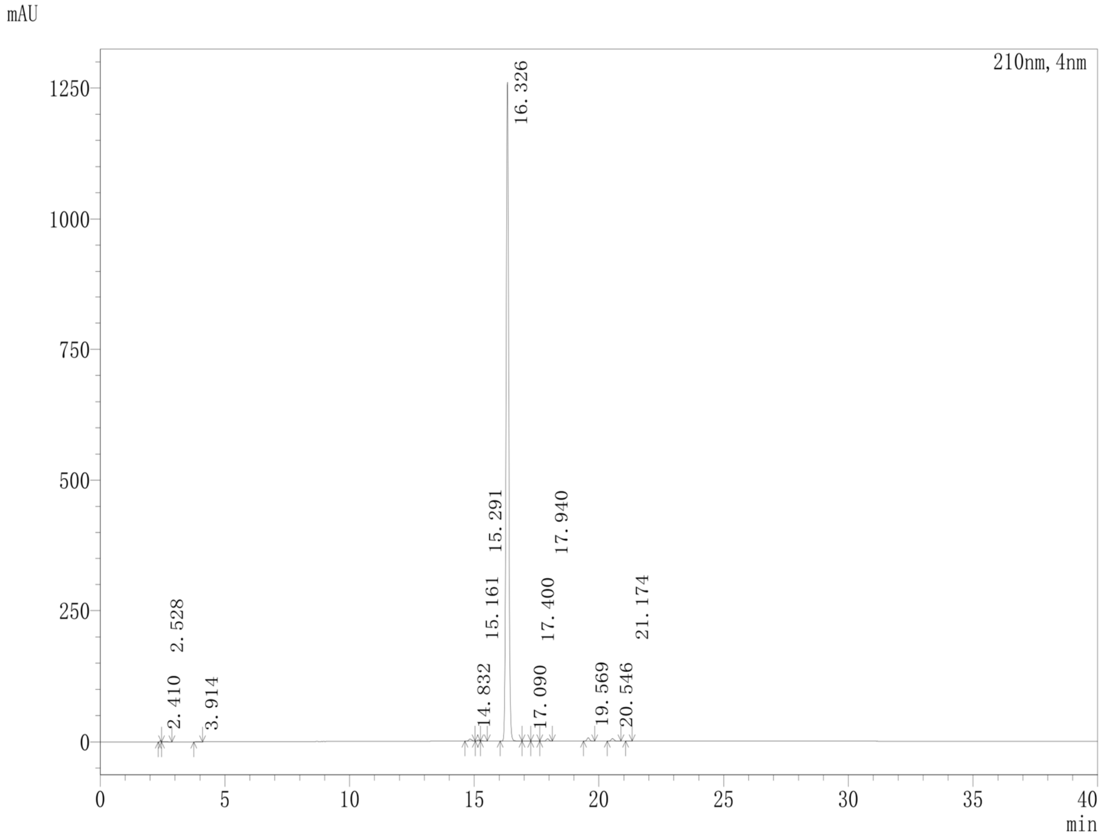

3.15 Compound **T$_4$-PZA**

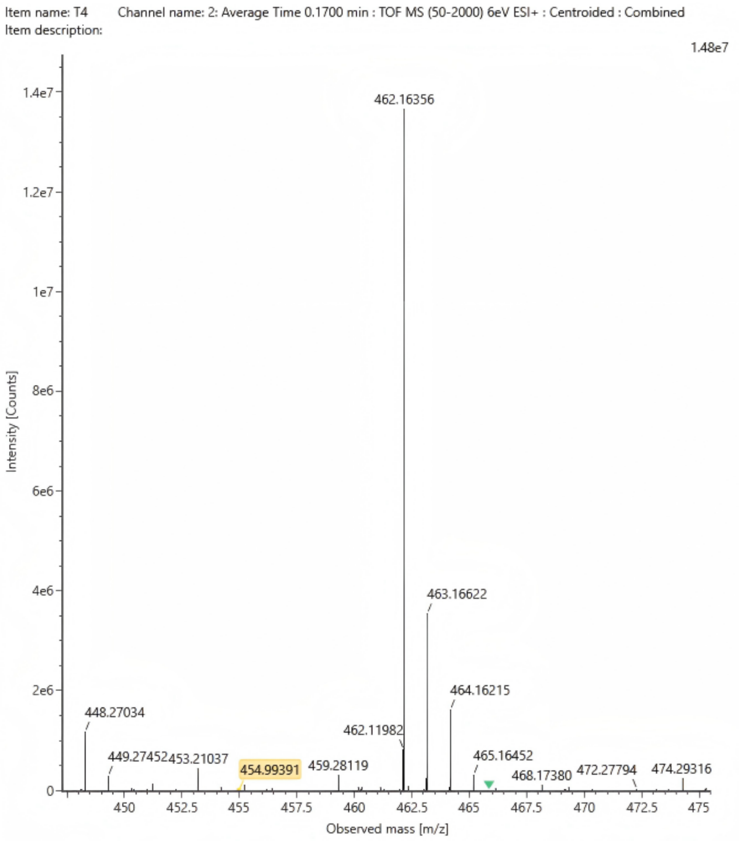

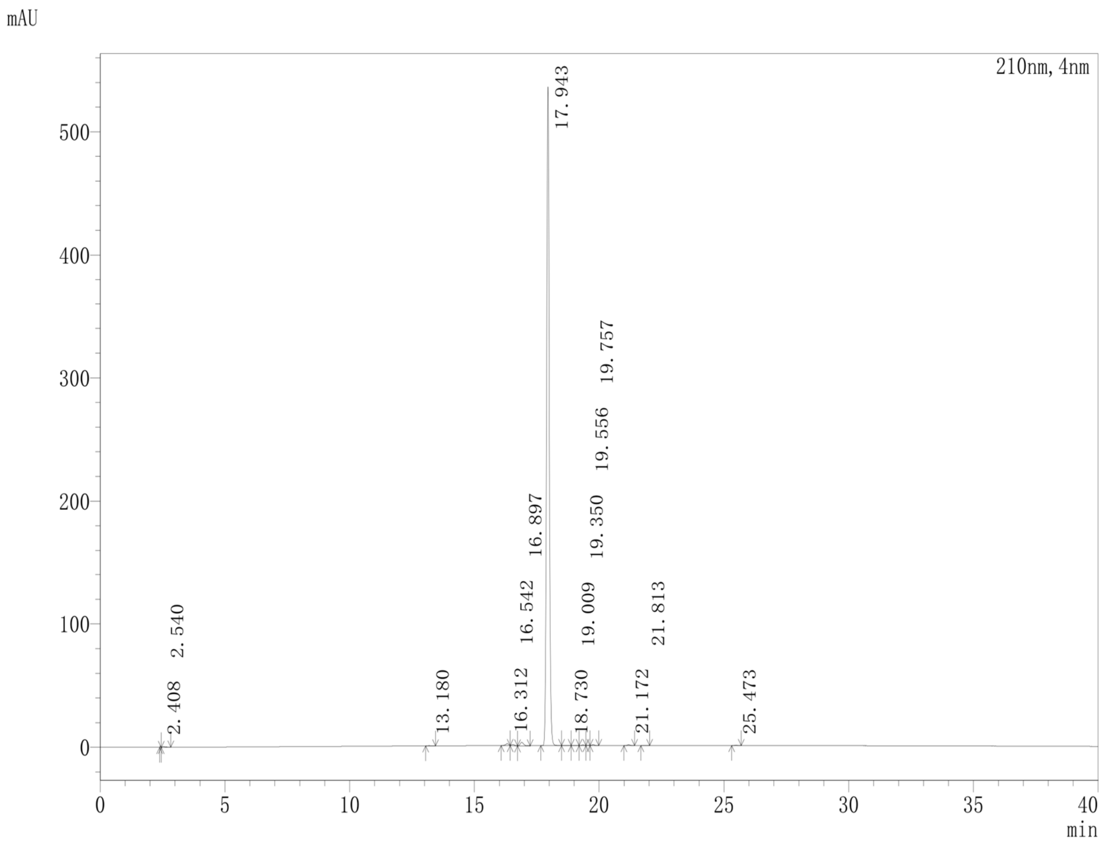
mAU
210nm,4nm
17.943
16.897
16.542
16.312
13.180
2.540
2.408
19.757
19.556
19.350
19.009
18.730
21.813
21.172
25.473
min
